# Understanding Diversity, Evolution, and Structure of Small Heat Shock Proteins in Annelida Through in Silico Analyses

**DOI:** 10.3389/fphys.2022.817272

**Published:** 2022-04-13

**Authors:** Mercedes de la Fuente, Marta Novo

**Affiliations:** ^1^ Departamento de Ciencias y Técnicas Fisicoquímicas, Universidad Nacional de Educación a Distancia (UNED), Las Rozas, Spain; ^2^ Faculty of Biology, Biodiversity, Ecology and Evolution Department, Complutense University of Madrid, Madrid, Spain

**Keywords:** stress physiology, small heat shock proteins, molecular evolution, α crystallin domain (ACD), dimeric architecture, earthworms, polychaetes, leeches

## Abstract

Small heat shock proteins (sHsps) are oligomeric stress proteins characterized by an α-crystallin domain (ACD). These proteins are localized in different subcellular compartments and play critical roles in the stress physiology of tissues, organs, and whole multicellular eukaryotes. They are ubiquitous proteins found in all living organisms, from bacteria to mammals, but they have never been studied in annelids. Here, a data set of 23 species spanning the annelid tree of life, including mostly transcriptomes but also two genomes, was interrogated and 228 novel putative sHsps were identified and manually curated. The analysis revealed very high protein diversity and showed that a significant number of sHsps have a particular dimeric architecture consisting of two tandemly repeated ACDs. The phylogenetic analysis distinguished three main clusters, two of them containing both monomeric sHsps, and ACDs located downstream in the dimeric sHsps, and the other one comprising the upstream ACDs from those dimeric forms. Our results support an evolutionary history of these proteins based on duplication events prior to the Spiralia split. Monomeric sHsps 76) were further divided into five subclusters. Physicochemical properties, subcellular location predictions, and sequence conservation analyses provided insights into the differentiating elements of these putative functional groups. Strikingly, three of those subclusters included sHsps with features typical of metazoans, while the other two presented characteristics resembling non-metazoan proteins. This study provides a solid background for further research on the diversity, evolution, and function in the family of the sHsps. The characterized annelid sHsps are disclosed as essential for improving our understanding of this important family of proteins and their pleotropic functions. The features and the great diversity of annelid sHsps position them as potential powerful molecular biomarkers of environmental stress for acting as prognostic tool in a diverse range of environments.

## Introduction

Heat shock proteins (HSPs) are a group of conserved proteins with crucial roles in the cell. They were first discovered because of their up-regulation during heat stress (hence the name), but they are now known to function in both stressed and unstressed cells as molecular chaperones required for protein folding during *de novo* protein synthesis and for the maintenance of proteome integrity and protein homeostasis (Feder and Hofmann, 1999; Sørensen et al., 2003). The HSP superfamily can be divided into several classes or families, each with a distinct evolutionary history (Kim et al., 2013; Waters, 2014; King and MacRae, 2015; Wu et al., 2016). In this study, we focus on one of these families: the small heat shock proteins (sHsps). sHsps are ATP-independent chaperones and range in size from 12 to 43 kDa (de Jong et al., 1998; Fu, 2014). Many sHsps have been shown to be developmentally regulated, and they can also be stress-induced and/or constitutively expressed (Kappé et al., 2002). sHsps are a critical part of the cellular chaperone network. They play an important supporting role in maintaining unfolded or misfolded proteins in a soluble and folding competent state by temporarily storing them through the formation of reversible sHsp/substrate aggregates. The release of substrate proteins from these transient reservoirs and the subsequent refolding require the cooperation of ATP-dependent chaperones (Nakamoto and Vígh, 2007; Basha et al., 2012; Carra et al., 2017). In addition, the sHsp family is involved in cellular stress management by controlling membrane stability via specific lipid interactions and regulating other aggregation processes by modulating the interaction spectra and functions of some conserved regulatory molecules, such as the 14-three to three proteins (Nakamoto and Vígh, 2007; Haslbeck et al., 2019).

sHsps are ubiquitous proteins but highly variable in number and diversity across organisms (Waters, 2014; Bakthisaran et al., 2015). Moreover, sHsps exhibit a variety of subcellular localizations and/or tissue distributions, bind a wide range of cellular substrates, and are involved in diverse cellular functions and defense mechanisms against many different stressors (Nakamoto and Vígh, 2007; Jaspard and Hunault, 2016), all suggesting diversified functions. Thus, the evolutionary mechanisms that led to the diversification of sHsps and their function in multichaperone networks are a subject of great interest (Kappé et al., 2002; Kriehuber et al., 2010; Waters, 2014; Obuchowski et al., 2019). Little is known about the evolution of sHsps. They evolve rapidly at the amino acid level and are more divergent than other HSPs (Kriehuber et al., 2010). Their relative lack of primary sequence conservation complicates amino acid alignments (and consequently comparison and tracking of evolutionary relationships) across sequences of proteins belonging to distantly related organisms (Waters, 2014). Currently, diverse information is available on sHsps in different groups of organisms. For example, a limited number of sHsp genes (often 1–3) has been reported in prokaryotes, although the sequence divergence in prokaryotes appears to be even greater than that in plants or animals (Kappé et al., 2002). Most prokaryotic sHsps function as chaperone-like proteins in the cytoplasm, but some are part of the spore coat or are associated with membranes (Tsvetkova et al., 2002; Nakamoto and Vígh, 2007; Obuchowski et al., 2021). A study of 113 sHsps from filamentous fungi led to the definition of eleven orthologous groups. The number of sHsps ranged from one to five in the species investigated. The phylogenetic analysis revealed gene duplication as an important mechanism of sHsp evolution and allowed clustering 102 of the 113 sequences into eleven groups (Wu et al., 2016). sHsps are well defined and characterized in higher plants, in which up to more than 30 individual sHsps per species can be found, classified into twelve conserved sHsp subfamilies based on their cellular localization (cytoplasm/nucleus or different organelles, such as the endoplasmic reticulum, peroxisome, chloroplast, or mitochondrion) (Waters, 2013; Jaspard and Hunault, 2016; Krsticevic et al., 2016; Yu et al., 2016; Cui et al., 2021).

Animal sHsps are thought to form a monophyletic group that originated evolutionarily from one unique class of bacterial sHsps (Fu et al., 2006). Ten sHsp subfamilies have been described in humans and other mammals (Fontaine et al., 2003; Kappé et al., 2003, 2010; Kampinga et al., 2009; Mitra et al., 2021). Only seven of these subfamilies appear to have orthologous groups in other vertebrates, but more than five novel sHsps have been identified in birds and fish (Franck et al., 2004; Elicker and Hutson, 2007), defining a total of 15 paralogous vertebrate sHsps resulting from successive gene duplications, all of which occurred before the divergence of teleost fish and tetrapods. sHsps of some invertebrate organisms have also been extensively studied, revealing genus- or even species-specific proteins for which no orthologs have been identified in other organisms, such as those of *Caenorhabditis* (Aevermann and Waters, 2008). sHsp families have also been identified and characterized in insects (Martín-Folgar et al., 2015; Morrow and Tanguay, 2015; Yang et al., 2021). It is important to note that some “unique” sHsps have been described in certain species (examples in (Siddique et al., 2008; Waters et al., 2008; Sarkar et al., 2009; Bondino et al., 2012), which have been postulated to be recent duplicates that will eventually be lost, ancestral genes that gave rise to the observed subfamilies, or potential new sHsp subfamilies in the early stages of evolution (Waters, 2013).

Complex gene families arise through evolutionary processes such as gene duplication, gene recombination, and gene loss (Nei and Rooney, 2005; Flagel and Wendel, 2009; Krsticevic et al., 2016; Yu et al., 2016; Cui et al., 2021). Individual sHsp subfamilies exhibit a diversity of evolutionary histories (for examples in plants, see Waters 2013). sHsp subfamilies that reflect the phylogenetic relationships of organisms in a given group are usually established subfamilies that also tend to conserve core functions (Waters, 2013). However, if orthology is not reflected by the phylogenetic relationships of a given sHsp subfamily, then gene duplication and loss are likely to occur independently across genomes (Waters et al., 2008; Bondino et al., 2012), and much greater diversity in substrate binding and function would be expected.

The diversity, evolution, and structure of sHsps in annelids are largely unknown. The Annelida, commonly referred to as segmented worms, are a highly diverse group comprising animals that live in a variety of habitats, from marine to freshwater to terrestrial environments. These environments potentially provide a wide variety of stressors that could activate sHsps. Annelid transcriptomes and genomes are currently available from previous studies and genome projects (Riesgo et al., 2012; Novo et al., 2013, 2015, 2016; Andrade et al., 2015; Lemer et al., 2015) and provide the opportunity to identify and characterize sHsps within an evolutionary context. In the present study, we aim to shed light on the evolution of sHsps in annelids by 1) capturing the diversity of these proteins in the group, 2) identifying the major evolutionarily stable subfamilies of these proteins, and 3) exploring sequence features and physicochemical properties of these subfamilies, and comparing them to sHsps described in other taxa.

## Materials and Methods

### Taxon Sampling and Sequence Identification and Translation

**FIGURE 1:**
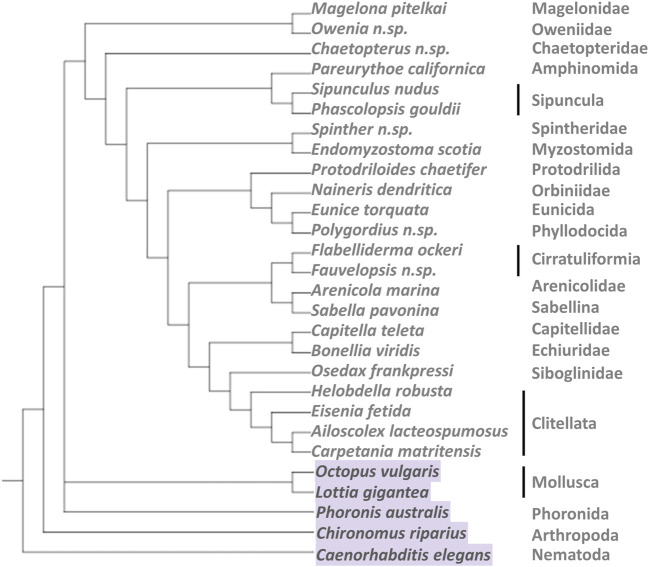
Tree modified from Figure 5 in Andrade et al. (2015) including the species covered in this study. Note that some of the internal relationships of the annelids are not yet well resolved, so this figure only gives an overview of the diversity covered within the group.

We compiled a data set of 23 annelid species that are well distributed in the annelid phylogenetic tree (Andrade et al., 2015) (Figure 1), consisting of 21 transcriptomes generated in previous studies (Riesgo et al., 2012; Krause, 2013; Novo et al., 2013, 2015, 2016; Andrade et al., 2015; Lemer et al., 2015) and two genomes from the JGI Genome Portal. In addition, genomic or transcriptomic information from five outgroups, ranging from molluscs and phoronids to nematodes and arthropods, was also included (Marinković et al., 2012; Riesgo et al., 2012; Krause, 2013; Andrade et al., 2015; Martín-Folgar et al., 2015). Detailed information can be found in Supplementary Table S1.

An initial search for sequences of small heat shock proteins (sHsps) from metazoans in annelids was performed using the NCBI Blast tool. It is generally accepted that the presence of the conserved α-crystallin domain (ACD) is a sufficient criterion for assigning a new sequence to the sHsp family (Caspers et al., 1995; Kappé et al., 2010; Kriehuber et al., 2010; Moutaoufik and Tanguay, 2021). Thus, we blasted the ACDs of all six curated sHsp protein sequences from the midge *Chironomus riparius* (we use these sHsps in this initial step because our previous work and experience with these proteins, see Martín-Folgar et al., 2015) against the GenBank database and retrieved similar annelid protein sequences (mainly from *Helobdella* and *Capitella* genomes). Using all of them, we constructed a database and performed an initial local BlastX against the transcriptomes of *Carpetania matritensis* and *Eisenia fetida* with an e-value cut-off of 1e^−5^. We then generated our own database of retrieved sequences potentially containing ACDs for annelids and performed local BlastX analyses, again with an e-value cut-off of 1e^−5^, against all the selected transcriptomes and genomes, including those from outgroups.

Next, the NCBI tools ORFfinder (https://www.ncbi.nlm.nih.gov/orffinder/), BLASTp, SmartBLAST (https://blast.ncbi.nlm.nih.gov/smartblast/smartBlast.cgi), and CD-Search **(** https://www.ncbi.nlm.nih.gov/Structure/cdd/wrpsb.cgi) (Marchler-Bauer and Bryant, 2004; Marchler-Bauer et al., 2011, 2015, 2017) were used to translate to proteins and to manually detect and ensure that the sequences found contained the conserved sHsp domain (i.e., the ACD); the rest of the sequences were discarded (approximately 80% of the sequences were discarded during this manual filtering). During this exercise, all the sequences from *Helobdella*, *Capitella,* and *Lottia* that were similar during the Blastp were also retrieved and reviewed in a similar manner. Annotated sHsps for *Caenorhabditis elegans* were retrieved from RefSeq by searching each gene locus of the sHsps (Krause, 2013) in the WBcel235 assembly of *C. elegans* genome in Ensembl (assembly accession GCA_000002985.3). The ACD sequences of sHsps *C. elegans* were added to all our analysis since these are one of the invertebrate sHsp family better annotated and described.

### Structure Predictions, Multiple Sequence Alignments and Phylogenetic Analyses

The secondary structure of each protein was predicted using the online services PSSPred v.3 and v.4 (https://zhanglab.ccmb.med.umich.edu/PSSpred/) (Yan et al., 2013). Based on the predicted secondary structure, the ACD of each protein was precisely defined. Initial amino acid alignment of the most conserved region (from the β3-to the β9-strand) of the 393 predicted ACDs was performed using ClustalW, with default parameters, as implemented in MEGA 7.0.14 (Kumar et al., 2016). The alignments were then manually optimized considering the predicted structural information. This alignment was used to reconstruct the phylogenetic relationships. The best model of amino acid substitution was examined using Modeltest-NG (Flouri et al., 2015; Darriba et al., 2020). Maximum likelihood (ML) phylogenetic analyses of protein sequences were conducted using RAxML-HPC BlackBox 8.2.10 (Stamatakis, 2014) and Mr Bayes v.3.2.6 (Ronquist and Huelsenbeck, 2003) as implemented in the CIPRES Science Gateway (Miller et al., 2010). LG + G + F was selected as the best model of amino acid substitution. Best-scoring ML trees were inferred under the selected model, and the support values were estimated with 100 replicates using the rapid bootstrapping algorithm. For Bayesian phylogenetic approach, parameters were set to twenty million generations and trees were samples every 1000th generation, using the default random tree option to initiate the analysis. Two independent runs were performed and all sample points prior to the plateau phase were discarded as burn-in. Trees were combined to build the maximum a posteriori probability estimate of phylogeny. iTOL v.6.1.1. (Letunic and Bork, 2007, 2021). was used for phylogeny visualization and editing.

The evolutionary history of the ACDs studied allows us to define various sHsp clusters that are conserved among the annelids. In this study, we focus on describing and characterizing the sHsps possessing a single ACD. These proteins were uploaded to GenBank and annotated. Their sequences and the accession numbers can be viewed in Supplementary File S1.

### Sequence Analysis: Conservation and Physicochemical Properties

Length, molecular weight, theoretical isoelectric point, and the grand average of hydropathicity (GRAVY) index (Kyte and Doolittle, 1982) were computed by means of the Sequence Manipulation Suite (Stothard, 2000) for those proteins presenting only one ACD. These parameters were calculated for the complete protein sequence and for the fragment of ACD used for phylogenetic analysis. To identify conserved regions, we used the WebLogo three program (Crooks et al., 2004) to create block logos of conserved amino acid residues from the multiple sequence alignment of each cluster of sHsps analyzed.

### Subcellular Location Predictions

Prediction of the subcellular distribution was done using sequence-based predictors, annotation- and homology-based predictors, and hybrid methods. Thus, the calculations were executed in the following web-based system servers: 1) BUSCA (Savojardo et al., 2018) (http://busca.biocomp.unibo.it/), which integrates methods of DeepSig, TPpred3, PredGPI, BetAware, ENSEMBLE3.0, BaCelLo, MemLoci, and SChloro; 2) LocTree3 (Goldberg et al., 2012, 2014), including LocTree2 approaches plus homology-based inference (https://rostlab.org/services/loctree3/); and 3) DeepLoc-1.0 with the “Profiles” option (Almagro Armenteros et al., 2017) (http://www.cbs.dtu.dk/services/DeepLoc/), a purely sequence-based method. The identification of sorting signals embedded in amino acid sequences was achieved by means of BUSCA (through TPpred3 and PredGPI), TargetP-2.0 (Armenteros et al., 2019) (http://www.cbs.dtu.dk/services/TargetP-2.0/),SignalP (Nielsen et al., 1997; Armenteros et al., 2019) (http://www.cbs.dtu.dk/services/SignalP/) and seqNLS (Lin and Hu, 2013) (http://mleg.cse.sc.edu/seqNLS/).

**FIGURE 2:**
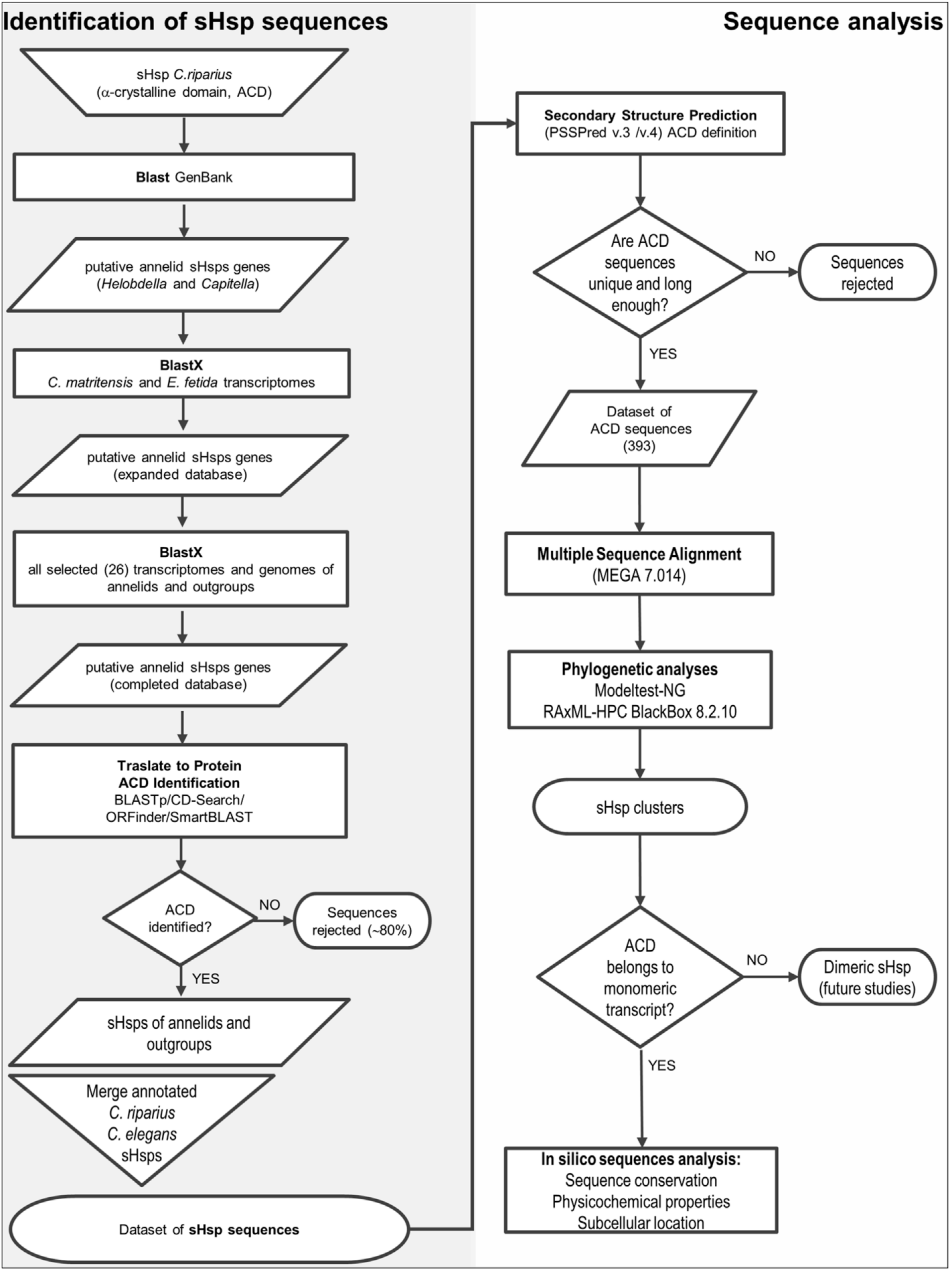
Flow chart of identification and analysis of sHsp.

A general flowchart illustrating, step by step, the identification and sequence analysis process has been included in Figure 2.

## Results

### Novel Protein Sequences

We analyzed 26 transcriptomes and genomes, including diverse taxa within the Annelida phylum (23), and three outgroups from the Mollusca and Phoronida phyla (Figure 1 and Supplementary Table S1). sHsp nucleotide sequences of an insect (*Chironomus riparius*) and of the nematode *Caenorhabditis elegans* were extracted from the literature and the Ensembl database, respectively, and were also incorporated in the study. We obtained 520 annelid nucleotide sequences containing α-crystallin domains (ACDs) (Supplementary Tables S2, S3). The open reading frames (ORFs) present in all these sequences were translated into protein code. The protein data set was manually curated and reviewed to ensure 1) that they contained one or more ACD domains and 2) that they corresponded to different proteins. Thus, a total of 228 new annelid sHsps were identified and classified. Remarkably, the majority of these sHsps contain a duplicate ACD (hereinafter “dimeric sHsps”), and just 76 of them resemble the most typical representatives of the sHsp family, containing just one unique ACD (hereinafter “monomeric sHsps”).

### Secondary Structure and Multiple Sequence Alignment

**FIGURE 3:**
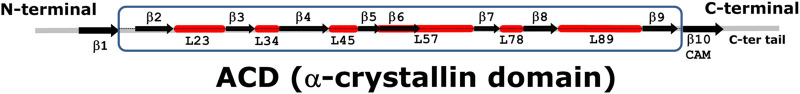
Representation of the structural topology of monomeric sHsps. ACD is the region delimited from the β2-strand to the β9-strand. The β1-strand is localized in the N-terminal region. The β10-strand corresponds to a conserved motif in the C-terminal region. The fragment starting after the ACD and including the β10-strand has been named the C-terminal anchoring module (CAM). Residues that follow the CAM were defined as the C-terminal tail. The β6-strand and the β10-strand are not universally present in all sHsp (Poulain et al., 2010). The black arrows represent the β-strands and the red line, the link between two β-strands.

The ACD domain, a hallmark of all sHsps, consists of a sandwich arrangement of two β-sheets organized in an immunoglobulin-like fold. The secondary structure of this domain consists of seven to eight well-conserved β-strands of various sizes. Flanking the ACD, two intrinsically disordered and variable regions, the N-terminal domain and a C-terminal extension, can be defined (Figure 3). Although the protein sequence is poorly conserved within the group, these structural elements are highly conserved. The protein sequence analysis of annelid sHsps showed that they contain one or two conserved ACDs, whereas the N-terminal and the C-terminal arms, as well as the linking region between the two ACDs in the dimeric proteins, are highly variable regions, as was expected.

Accurately aligning highly variable and distant protein sequences is extremely difficult. To obtain an optimal multiple sequence alignment, it is crucial to consider the most conserved regions and the more conserved elements. Accordingly, we predicted the secondary structure of each sequence and, consequently, were able to 1) define the ACD boundaries and 2) identify the well-conserved secondary structural elements (β-strands), which was valuable information for sequence alignment construction. Thus, 393 ACDs were extracted from the 228 identified annelid sHsps and the 54 sHsps of other species (outgroups). All these ACDs were used to create a multiple sequence alignment, using, simultaneously, ClustalW via MEGA 7.0 and manual editing, considering the available structural information, i.e., the identified well-conserved secondary structural elements (β-strands). The amino acid alignment is available from the authors. Finally, the alignment extends from the β3-to the β9-strand, since the β2-strand is not well conserved among all the sequences.

### Phylogenetic Analyses

**FIGURE 4:**
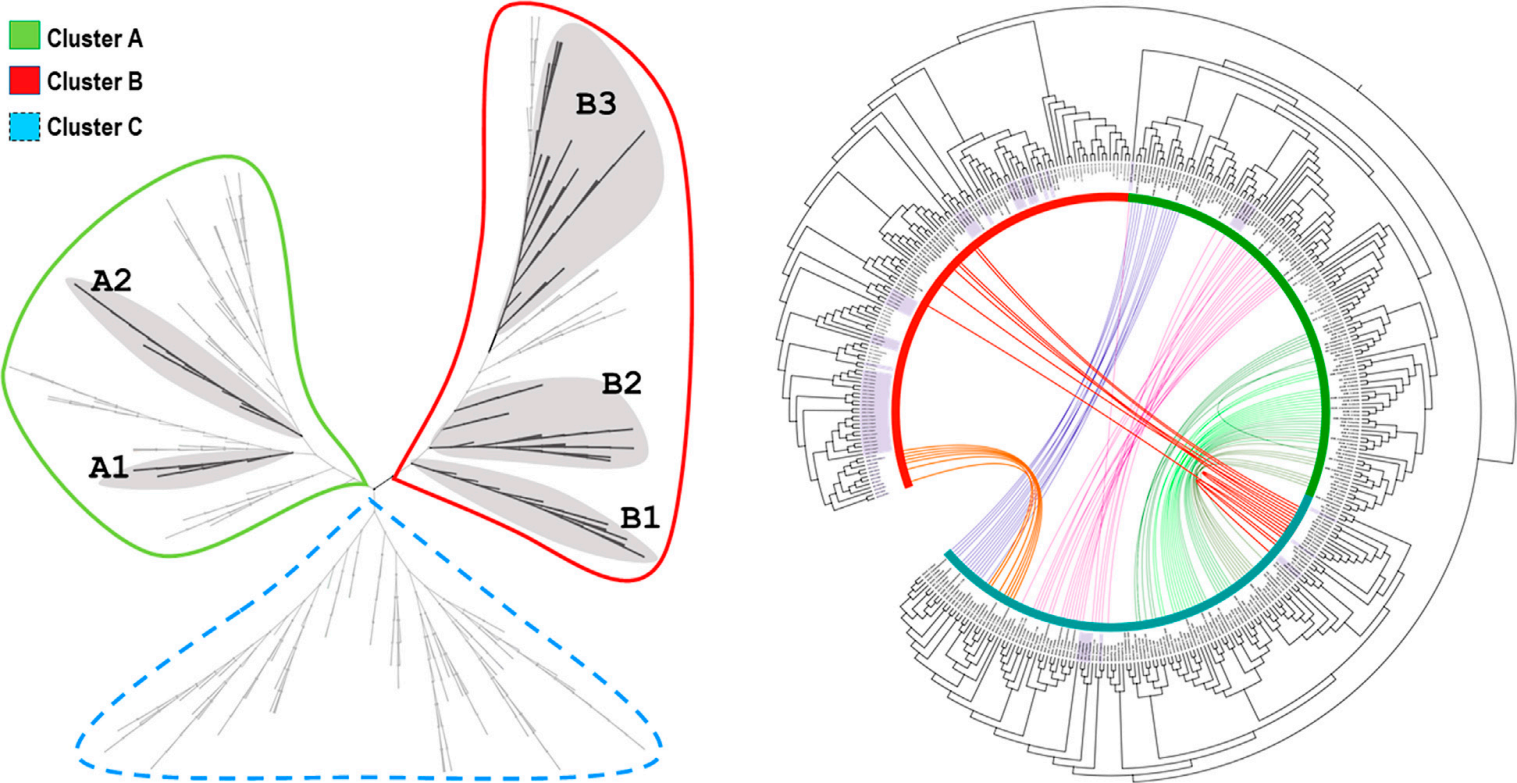
ML tree generated using the multiple sequence alignment of the ACD data set **(A)** Left, unrooted ML phylogenetic tree and clustering branching pattern of monomeric sHsps (shadowed clades) **(B)** Right, circular ML phylogenetic tree showing the location of the outgroups’ ACDs (shadowed labels), the colored connection lines between the ACDs belonging to the same dimeric protein, and the colored stripes (green, red and blue) marking the three big clades: Cluster A, B and C, respectively. Branch lengths are not displayed. More details can be found in File S2, including the results of the Bayesian phylogenetic study, the dendrograms of the analyzed clusters, and the node support information.

The curated data set of ACDs was used to construct maximum likelihood (ML) and Bayesian phylogenetic trees. The sequences for the analysis comprised 96 positions, and the proportion of gaps or indeterminate characters was 23.32%. The resulting global trees are shown in Figure 4 and Supplementary File S2. The unrooted ML tree showed three big clades (Figure 4A). Interestingly, one of them included mostly the ACDs located upstream of the dimeric sHsps (Cluster C). Sequences from the outgroups were identified mainly in Cluster B, with only a few sequences from the Mollusca and Phoronida placed in Clusters A and C. Since the most distant outgroups (Nematoda and Arthropoda) are placed only in Cluster B, the global tree was rooted with this clade, and this rooted version is shown in Figure 4B, which also includes the connections between the ACDs belonging to the same dimeric protein.

**FIGURE 5:**
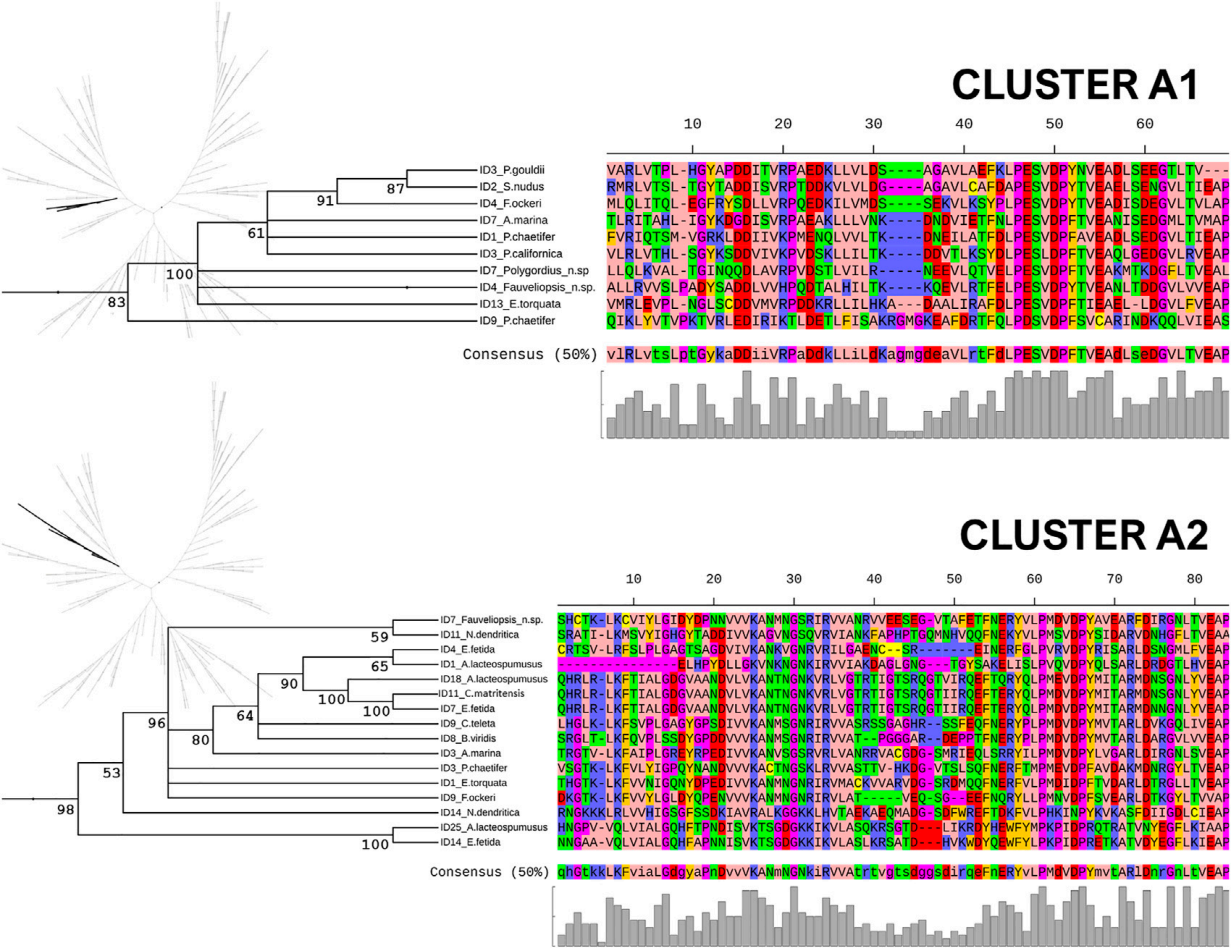
Clusters A1 and A2. Multiple sequence alignment visualized on a dendrogram obtained from the Bayesian tree, using the Zappo coloring scheme. The consensus sequence (at 50% conservation) and residue conservation were calculated by iTOL. On the left, the unrooted radial ML cladograms, in which each cluster is highlighted. The posterior probability values are included as percentages in the nodes.

**FIGURE 6:**
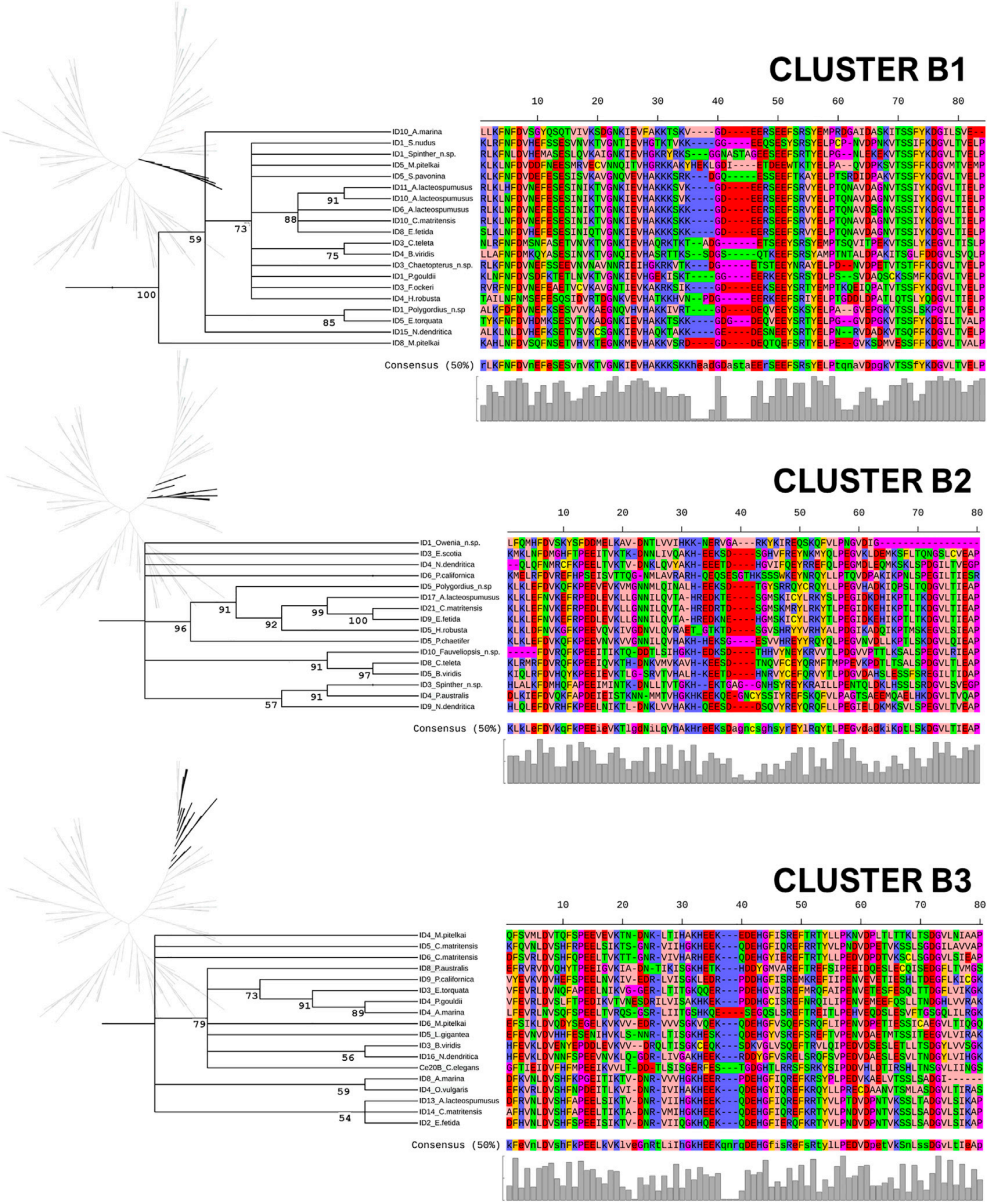
Clusters B1, B2, and B3. Multiple sequence alignment visualized on a dendrogram obtained from the Bayesian tree, using the Zappo coloring scheme. The consensus sequence (at 50% conservation) and residue conservation were calculated by iTOL. On the left, the unrooted radial ML cladograms, in which each cluster is highlighted. The posterior probability values are included as percentages in the nodes.

The Annelida show a great diversity of sHsps, as reflected in these trees, and the ACDs from monomeric and dimeric forms regularly cluster together. In this study, we focus on the identification and analysis of the more typical and widely distributed monomeric sHsps. Thus, five subclusters of monomeric sHsps could be identified: A1 and A2 in Cluster A and B1, B2, and B3 in Cluster B (Figure 4). Clusters were assigned based on ML and Bayesian trees analysis. Bayesian node support were high at the base of the cluster A1, A2 and B1 (see Figures 4, 5, and Supplementary File S2, Supplementary Figures S4, S7, S10). ML node support were also high for clusters A1 and B1 (see Supplementary File S2, Supplementary Figures S3, S9). The rest of monomeric sHSPs were including in cluster B2 and B3. These both clusters involved most of the outgroups sHSPs. Despite the phylogenetic distinction between cluster B2 and B3 it is not well supported (maybe because the existence of those more distant sequences), for our subsequent sequence analysis we differentiate between B2 and B3 clusters based on the ML tree structure (see Supplementary File S2, Supplementary Figures S12, S14). The number of copies per species and sHsp group can be found in Supplementary Table S2. A1, A2, and B1 seem to be unique to the Annelida, at least considering the data analyzed. Within A1, sequences from many Sedentaria (terrestrial annelids and closely related forms) are missing, while A2 is composed of sequences of Errantia and Sedentaria, but ACDs from Magelona, Sipunculus, Spinther and so on (more basal groups, according to Andrade et al., 2015) are missing (Supplementary Table S3). B2 contains the Cri21 sHsp of the insect *C. riparius* and the three isoforms C25a, C25b, and C25c of *C. elegans*, as well as other sHsps from the other three outgroups: *P. australis*, *L. gigantea*, and *O. vulgaris*. B3 contains the rest of the sHsps of *C. riparius* and *C. elegans,* plus one representative of the sHsps *O. vulgaris, L. gigantea*, and *P. australis*. The analyzed ACDs do not seem to contain phylogenetic information within the Annelida probably because of their high variability, and node supports are generally low. However, the sequences of the earthworms appear clustered and are generally well supported within the different groups (Figures 5, 6). Figures 5, 6 show the multiple sequence alignment visualized on the dendrograms obtained from the Bayesian tree, the consensus sequence and residue conservation, and the radial ML cladograms of each cluster.

### Sequence Analysis: Conserved Residues, Physicochemical Properties, and Subcellular Location

The theoretical pI, length, molecular weight, and grand average of hydropathicity (GRAVY) were calculated for each annelid monomeric sHsp and its respective ACD (between β3 and β9). Violin plots of these parameters for the different clusters are included in Supplementary File S2 (Supplementary Figures S15, S16). Interquartile ranges of the main parameters are compiled in Table 1.

**TABLE 1:** Physicochemical properties of monomeric sHsps of annelids. Sizes (MW: molecular weight), isoelectric points (pI), and GRAVY values for the whole protein and the ACD region (between β3 and β9) are indicated. Tentative subcellular localizations for each cluster are also shown.

Cluster	Number of Proteins Analysed^a^	MW^b^	Protein Length (aa)^b^	Protein pI^b^	ACD pI^b^	Protein GRAVY^b^	ACD GRAVY^b^	Tentative Subcellular localisation^c^
**A1**	9	9.7–10.1	89–91	4.1–4.3	3.9–4.1	−0.12 to −0.04	0.01 to 0.15	Nucleus/Cytoplasm
**A2**	10	40.0–41.8	359–381	9.2–10.2	8.6–10.1	−0.69 to −0.54	−0.31 to −0.16	Nucleus/Cytoplasm
**B1**	18	16.3–25.5	142–241	4.9–6.0	4.8–5.2	−0.72 to −0.46	−0.75 to −0.62	Mitochondrion/Nucleus/Cytoplasm
**B2**	13	17.9–23.0	158–204	5.7–7.5	6.0–8.7	−0.66 to −0.58	−0.75 to −0.61	Mitochondrion/Cytoplasm
**B3**	13	21.3–23.9	187–205	6.4–8.0	4.5–6.0	−0.80 to −0.65	−0.59 to −0.44	Mitochondrion

GRAVY (grand average of hydropathy).

a Incomplete sequences were not considered.

b Interquartile range (see Supplementary File S2, Supplementary Figures S15, S16).

c Consensus based on subcellular localization predictions (see Methods section and Supplementary File S3).

It is generally accepted that subcellular location is one of the main aspects defining protein function, since the environment of a protein provides the physiological context for its function (Kumar and Dhanda, 2020). The results of the prediction of subcellular distribution are collected in Supplementary File S3. In recent comparative benchmarks, using an animal and fungi data set (Salvatore et al., 2018; Savojardo et al., 2018), BUSCA seems to perform better for the nucleus, the endomembrane system, and the cytoplasm, whereas LocTree3 tends to perform better for mitochondrial proteins but over-predicts cytoplasmic proteins. DeepLoc outperforms other methods in extracellular compartments, plasma membrane compartments, lysosomes, and peroxisomes. Considering this information and the relative scores of each individual prediction, a tentative location for the different classes of sHsps is proposed in Table 1.

**FIGURE 7:**
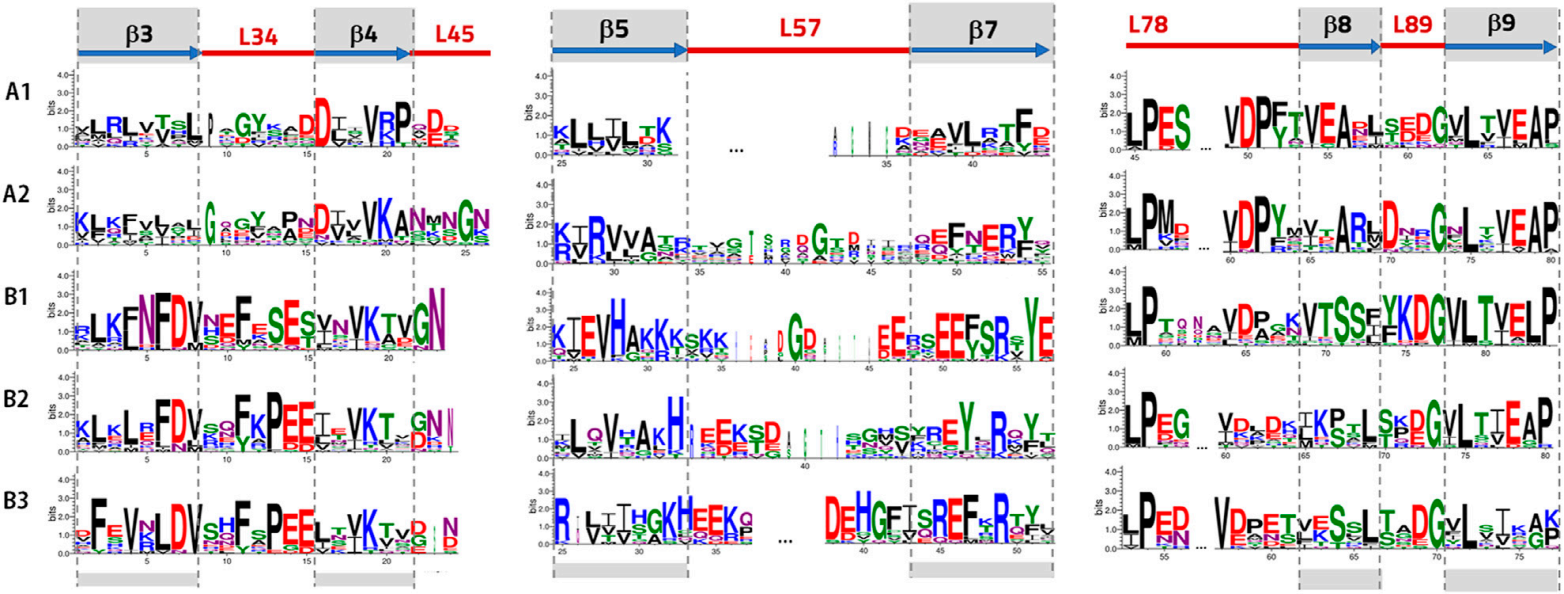
Logo presentation using the WebLogo3 program (Crooks et al., 2004) for Clusters A and B. The height of each letter is proportional to its frequency. Amino acids are colored according to their chemical properties: acidic **(D, E)** in red, basic **(K, R, H)** in blue, polar **(G, S, T, Y, C)** in green, neutral **(N, Q)** in purple, and hydrophobic **(A, V, L, I, *p*, W, F, M)** in black. Logos for Clusters B2 and B3 are calculated without including the sequences from the outgroups (Supplementary File S2, Supplementary Figure S17-S19 include logos for Clusters B2 and B3 calculated including the outgroups, but the differences are not significant.).

To improve the visualization and analysis of the conserved regions in the ACDs for each cluster, blocks of the most conserved residues were represented as logos. They are shown in Figure 7.

## Discussion

We have, for the first time, identified and characterized sHsps in annelids. A great diversity of sHsps was discovered, and the sequences of 393 ACD fragments were included in our analyses (see Supplementary Table S2). Most of these ACDs 272) belong to sHsp transcripts with two consecutive ACDs, which would result in putative proteins with a dimeric architecture. In this study, we focus on the characterization of the typical and widely distributed monomeric sHsps (76 were identified in annelids), but the wide diversity of dimeric sHsps identified in annelids provides an interesting starting point for further studies. We would like to emphasize that the data set used was meticulously curated, with each sequence included in the analyses being manually revised before and after preliminary phylogenetic analysis. The variety found is notable; however, we cannot rule out the existence of additional sHsp sequences that were not detected because they may not be present in the revised data set (e.g., not transcribed under the given conditions or not sequenced). Nevertheless, this study provides a solid background for further research on sHsps in annelids.

It is well known that sHsps evolve very rapidly at the amino acid level, particularly the N- and C-terminal regions (Waters, 2013). This complicates the sequence alignments across distantly related organisms. We have indeed observed this phenomenon and identified highly divergent sHsp sequences within the Annelida. Despite the considerable sequence divergence, the structural features of these proteins are conserved (Waters, 2014) and have been shown to be particularly useful for guiding alignments. The ACD is the most conserved domain and is typically used for evolutionary analyses (Caspers et al., 1995; de Jong et al., 1998; Kriehuber et al., 2010; Martín-Folgar et al., 2015). We confirmed this in our data, and the phylogenetic trees are based on this domain of the protein.

### A Dimeric Architecture With Two Tandemly Repeated ACDs Is Ubiquitous Rather Than Rare in Annelid sHsps

As mentioned, an interesting feature that we uncovered is that annelids present many putative dimeric sHsps, with two consecutive ACDs. The phylogenetic ML tree revealed the presence of three main clusters. Intriguingly, one of them (Cluster C) included mainly ACDs belonging to dimeric forms, and, significantly, all these ACDs are located upstream in the transcripts. The other two (Clusters A and B) included ACDs belonging to both monomeric and downstream ACDs from the dimeric forms, which were shown to be closely related. Within these main clusters, we identified two differentiated subclusters of monomeric sHsps in Cluster A (named A1 and A2) and three subclusters in Cluster B (named B1, B2, and B3). Within Cluster C, there is a coherent phylogenetic arrangement, and the duplicated ACDs of different subgroups are normally clustered together. Some dimeric forms are also found in mollusks and phoronids but not in nematodes and arthropods. As far as we know, among all the sHsps in all domains of life, this dimeric architecture has previously been reported only in some Platyhelminthes (Caspers et al., 1995; Stamler et al., 2005), in which these proteins have been related to the self-protection and pathogenicity of these parasites. Thus, our findings would be in agreement with gene duplication events prior to the Spiralia split, followed by an extensive diversification in annelids, leading to a large dimeric sHsp subfamily. It should be emphasized many members of the sHsp family tend to exist as an ensemble of large oligomers, with dimers, monomers, or a combination of both considered to be the basic building block for oligomer assembly (Stamler et al., 2005; Hochberg and Benesch, 2015). Under certain cellular conditions, the sHsp ensemble breaks into smaller subunits and becomes activated, with the dimers considered to be the main active forms with exposed substrate binding sites (Haslbeck et al., 2019). The X-ray structure of Tsp36, the dimeric sHsp of *Taenia saginata*, revealed relevant information about the mechanism of dimerization in metazoan sHsps and its implications for function (Stamler et al., 2005). Further studies could unravel the sequence conservation, as well as the impact of sequence divergence on the structure, within the abundant and evolutionarily related dimeric sHsps of annelids found in our study. These studies may provide insight into the mechanism of action of this diverse family, either regarding the mode of assembly or regarding substrate interactions.

In this study, we have compiled and curated the sequences belonging to clusters involving only monomeric forms. Thus, 76 new sHsps were characterized in silico from the Annelida. Sequences from some of the outgroups were also new. All these monomeric sHsps, compiled in Supplementary File S1, have been made available to the scientific community to facilitate future studies on the evolution and function of these proteins. The analysis of sequence conservation and physicochemical properties and the prediction of subcellular localization allowed us to support the differentiation and homogeneity of each proposed subcluster. Based on the data analyzed, Clusters A1, A2, and B1 are found exclusively in annelids. These three groups are strongly supported in the Bayesian tree (posterior probability >83; see Figures 4, 5 and Supplementary File S2). Further analyses will reveal whether these are truly “unique” evolutionary novelties for annelids or whether they are shared with other metazoans. Moreover, ACDs from A1 and A2 seem to be present in different clades of the Annelida. A1 is composed of proteins from more basal species in the annelid tree of life (see Andrade et al., 2015; many Sedentaria, including terrestrial forms, are missing). In contrast, A2 comprises ACDs of Errantia and Sedentaria without basal species (see Supplementary Table S3). Whether this is a true pattern and what its biological meaning is will need to be confirmed in the future. Clusters B2 and B3 are somewhat more complex. They include representatives of organisms whose sHsps where previously studied (the nematode *C. elegans* and the midge *C. riparius*), as well as other outgroups included in the analyses (mollusks and phoronids). Although the internal phylogenetic relationships are not well resolved by the ACDs analyzed, the sequences from earthworms do cluster together in the trees with high support values. Habitat differences and soil uniformity may be related to this result, with sHsps of terrestrial forms being more related and uniform.

### Molecular Weight, Isoelectric Point, and the Grand Average of Hydropathy Index: Sequence-Derived Physicochemical Features That Clearly Distinguish sHsps From Clusters A1, A2, and B

Characterization of the physicochemical properties of proteins is essential for identifying the functions and properties of proteins. To assess the common and different features of the defined sHsps clusters for annelids including monomeric forms, we performed a comparative analysis of the size, pI, and total hydrophobicity distribution. We found an ACD length between 71 and 79 residues in the A2, B1, B2, and B3 subclusters (Supplementary Figure S15). Considering that the β2-strand has not been included, the length of the ACDs is in line with the values previously reported for animals (Poulain et al., 2010), with the ACD length distribution centered at 83 residues. Remarkably, Cluster A1 exhibits shorter ACDs (63–69 residues). This is due to the very short L57 loop (see the multiple sequence alignment in Supplementary File S2). The proteins in this cluster are characterized by very small N-terminal and C-terminal domains, making them the smallest sHsps analyzed (MW: 9.7–10.1, Table 1), a distinguishing feature of this group. On the other hand, the sHsps in Cluster A2 are larger proteins (MW = 40.0–41.8, Table 1). A variety of molecular weights is found in Cluster B, ranging from 12.4 to 38.6 (Supplementary Figure S15). These differences are due to the divergent and variable length of the N-terminal and C-terminal domains. Since these regions play important roles in the structure, regulation, and chaperone function of sHsps (Kriehuber et al., 2010; V.; Sudnitsyna et al., 2012), it might be hypothesized that this variable length is associated with functional variability in the sHsps in Cluster B.

The isoelectric point (Supplementary Figure S16) indicates that members of the annelid sHsps in Cluster A1 are very acidic (pI: 4.1–4.3, interquartile range, Table 1), whereas in Cluster A2 they have basic isoelectric points (pI: 9.2–10.2, interquartile range, Table 1). Again, much more variation is found in Cluster B, in which the pI ranges from 4.4 to 10.2 (Supplementary Figure S16). The acid–basic properties of only the ACD regions are similar to those of the whole protein for each cluster. Analyses of a wide range of proteomes (Kiraga et al., 2007) indicated that the isoelectric point of proteins show clear relationships with the length of proteins, their subcellular localization, and the taxonomy and ecology of the organisms in which they are found, concluding, among other things, that acidic proteins are significantly longer than basic ones. The length-pI relationship in Clusters A1 and A2 is different from this general trend, but both parameters are clear features that allow these two groups to be defined and distinguished.

We examined the total hydrophobicity in the defined clusters using the GRAVY index (Supplementary Figure S16). Hydrophobicity is an important property in the sHsp family because the molecular role of sHsps in cellular stress is directly linked to their ability to bind unfolded substrate proteins via interactions with hydrophobic regions (Mymrikov et al., 2017). A positive GRAVY score indicates a globally hydrophobic protein, whereas a negative GRAVY score is related to more hydrophilic proteins (Rehman et al., 2020). Proteins in Cluster B are more hydrophilic, while Cluster A comprises proteins that are more hydrophobic (see Table 1 and Supplementary Figure S16). The proteins in Cluster A1 are the most hydrophobic.

Therefore, the very small and acidic proteins in Cluster A1 may be clearly distinguished from the largest and basic sHsps in Cluster A2. More diverse and in-between sizes and pI values are found in proteins from Cluster B. Cluster A1 includes the most hydrophobic proteins, and Cluster B has the less hydrophobic proteins. These differences in pI, GRAVY index, and size, consistent with the phylogenetic groups defined, could justify the functional properties of these proteins, and this information could help to design additional experiments to unravel their functional diversity. Moreover, the relevance of physicochemical properties for the annotation and classification of the sHsp family has been recognized earlier (Jaspard and Hunault, 2016; Mitra et al., 2021), so our analysis is in good agreement with this. Thus, it appears that the physicochemical properties studied are valuable data for classifying and establishing putative correspondences between sHsps of different organisms.

### Subcellular Distribution: Putative Nuclear and Mitochondrial sHsps in Annelids

The prediction of the subcellular distribution of all the identified sHsps in annelids suggests putative differential functions of these proteins and provides an interesting basis for further research on annelid sHsp genes and gene families. Our bioinformatic analysis reveals that the proteins in Cluster A are putative nuclear/cytoplasmic proteins. Nuclear localisation signals (NLSs) are predicted in some of the proteins in Cluster A1 and in most of the sHsps of Cluster A2 (results in Supplementary File S3). For the proteins in Cluster A1, these NLS sequences are located in the fairly well conserved motif **V-(R/K)-P**, which is located in the β4-L45 zone (see Figure 7 and Supplementary Figure S17), whereas a classical NLSs rich in basic amino acids are predicted in the N-terminal or C-terminal region of the sHsps of Cluster A2. In addition, high-scoring NLS sequences are predicted in some Cluster B proteins. Significantly, putative NLSs with the highest score are predicted in Cluster B1. These NLSs are lysine-rich motifs located in the β5-L57 zone (Figure 7). Similarly, the nuclear localization of sHsps that relies on short basic amino acid motifs located in the β5-and β6-strands of ACD was previously reported in plants (Siddique et al., 2003). Moreover, the relocation of cytosolic sHsps to the nucleus under certain stress conditions has been described in mammals, and conserved arginine-rich NLSs in the N-terminal region of sHsps of a variety of insect species have recently been characterized (Moutaoufik and Tanguay, 2021). Thus, it has been suggested that some sHsps not only play the role of molecular chaperones but are also likely to be involved in various nuclear processes, such as chromatin remodeling and transcription (Moutaoufik and Tanguay, 2021). Further comparative studies on the putative annelid nuclear sHsps reported in this study should certainly provide key insights in this regard.

Interestingly, the subcellular prediction by DeepLoc led us to suggest a mitochondrial location for many sHsps of Cluster B. Furthermore, a mitochondrial transit peptide at the beginning of the N-terminal domain is predicted for six of the 14 annelid sHsps in Cluster B3. Mitochondrial sHsps are widespread in plants but are rarely found in other eukaryotes, with the exception of mitochondrial sHsps in *Drosophila melanogaster*, which accumulate during stress and ageing (Avelange-Macherel et al., 2019). In agreement with our results, mitochondrial sHsps would be expected to be ubiquitous rather than peculiar in annelids.

### Conserved Residues and Motifs in ACDs: Annelid sHsps In-Between Plants and Animals

The logo representation of ACDs in Figure 7 highlights the most conserved residues and shows some interesting sequence and motif features. Consistent with previous findings (Poulain et al., 2010), the highly conserved residues in all the analyzed ACDs are located mainly in the β7-β9 zone. Thus, the two doublets **L-P** and **V-D** in the L78 loop, a **Gly** (**G**) residue in the L89 loop, and a motif in β9 (**L-X-(V/T)-(E/K)-(A/L)-(P/K)**) appear to be highly conserved in all the clusters. Likewise, an arginine residue (**R**) in β7, which has been associated with human pathologies, is clearly conserved in most annelid sHsps (except for those within Cluster A1). Other residues appear to be highly conserved but are not common properties of all annelid sHsps. Significantly, Cluster B contains much more sequence similarity with animal sHsps, while Cluster A presents features found in plants and bacterial sHsps. Thus, residue **Gly** in L34 and residue **Ala** in β8 appear in Cluster A but not in Cluster B. They are both residues that are not prevalent in animals but are well conserved in sHsps of plants and bacteria (Poulain et al., 2010). On the other hand, the **Phe (F)** residues in L34 and the serine-rich motif in β8 **(S-(S/T)-(F/L)**), observed in animals, appear in Cluster B. Moreover, specific motives, such as **L-D-V-X-X-F-X-P-E-E in the** β3-L34 zone and **G-K-H-E-E-(R/K)** in the β5-L57 loop, appear to be particularly well conserved in Cluster B3 (Figure 7), which included most of the outgroups. Interestingly, the fragment L34 has been associated with substrate binding in some mammalian sHsps, and β8-strands have been linked to the oligomerization process (Poulain et al., 2010). Therefore, both fragments appear to be functionally relevant.

Annelids possess well-conserved **V-K** residues in β4. The conservation of these residues is observed in sHsps of plants and animals but not in bacterial sHsps (Poulain et al., 2010). On the other hand, some motifs seem to be representative of our clusters. The most remarkable divergence in our ACD sequences can be observed in the β5-L57-β7 zone. Thus, as mentioned above, Cluster A1 has a very short L57 loop (only 5–6 residues long); Cluster B1 shows a conserved lysine-rich motif (**K-K-K-X-K-K**) in β5-L57, along with an acidic-rich region in L57-β7 (**E-E-X-X-E-E**); while the logo sequence in the β5-L57-β7 zone of Cluster B2 and, especially, Cluster B3 matches with the logo representation of animal ACDs (**G-K-H-E-E-(K/R)**.**D-E**. **H-G-X-X-X-R-E-F**) (Poulain et al., 2010). The well-conserved serine-rich motif **F-X-S-E-S** in the L34 loop of Cluster B1 must also be highlighted, which distinguishes this subfamily from B2 and B3.

To summarize, our results are consistent with the significance of the ACD region, the hallmark of the sHsp family, both from a sequence and a structural point of view (Poulain et al., 2010; Mitra et al., 2021). Significantly, these features allow us to distinguish annelid sHsps that share characteristics with animal sHsps (Cluster B, particularly those in Clusters B2 and B3), while other sHsps could be unique to annelids (Cluster A), with sequences that share characteristics with plant and some bacterial sHsps. Moreover, some specific sequence properties can be identified in each proposed group. The most striking differences are found in structural elements that can be functionally relevant, such as L34 or the β8-strand.

## Conclusion

Our study is the first bioinformatic analysis that reveals the great diversity and evolution of the sHsp family in annelids. Our results indicate that sHsps containing duplicated ACDs are abundant in annelids. Three main clusters were distinguished by phylogenetic analyses, one of them containing mostly the ACDs located upstream in the dimeric sHsps and the other two comprising downstream ACDs from dimeric sHsps and the ACDs from the monomeric forms. Since all the upstream ACDs cluster together, and the dimeric architecture is widespread in the species studied, a duplication prior to the annelid lineage divergence is a possible mechanism for the evolution of these proteins. The analysis of the dimeric forms is deferred to future work. The analyzed monomeric sHsps show that in one cluster the sequences exhibit features similar to those previously described in metazoan sHsps, while in the other one the sequence characteristics resemble plant and bacterial sHsps. Furthermore, five subclusters of monomeric sHsps were described. Homology studies at the sequence level, subcellular location predictions, and physicochemical properties allow us to consolidate and clarify the differences and similarities among these proposed sHsp subfamilies. Consequently, a nuclear/cytoplasmatic location is predicted mainly for those sHsps with non-metazoan sequence features (Cluster A), distinguishing very small and acidic proteins (Subcluster A1) from the largest and basic sHsps in Subcluster A2. On the other hand, a mitochondrial/cytoplasmatic location is predicted for proteins in Cluster B, which exhibit more varied physicochemical properties. Three subclusters have been defined in Cluster B, one of them (B1) involving only annelid proteins, and the other two containing proteins from annelids and the outgroups. These phylogenetic patterns point to sHsps previously described in invertebrates, such as the proteins homologous to the proteins in Cluster B. These findings locate annelid sHSPs in an interesting evolutionary position between animal and plant sHSPs and provide an excellent initial step towards enhancing our understanding of the evolution and functional divergences in this family of proteins.

## Data Availability

The datasets presented in this study can be found in online repositories. The names of the repository/repositories and accession number(s) can be found below: All newly-reported sequences in this study can be found in GenBank (https://www.ncbi.nlm.nih.gov/genbank/). The accession numbers are MZ261736 to MZ261811.
