# Supplementary material for: Understanding Diversity, Evolution, and Structure of Small Heat Shock Proteins in Annelida Through in Silico Analyses
Source: Front Physiol. 2022 Apr 13;13:817272. doi: 10.3389/fphys.2022.817272 (PMC9075518; doi:10.3389/fphys.2022.817272)
Supplement: Supplementary file 1 [file DataSheet1.ZIP › SM_MFuente&MNovo/File_S2.pdf]

Supplementary Material

Content index

This PDF file includes:

1 **Legends for Other Supplementary Materials**
2 **Phylogenetic trees, Predicted Secondary Structures and Multiple Sequence Alignments for Cluster A1, A2, B1, B2 and B3. Includes Figures S1 to S14.**
3 **Violin plots** of theoretical pI, length, molecular weight, and grand average of hydropathicity (GRAVY) of annelid monomeric sHsps and their respective ACDs (between β3 and β9) for each cluster. **Includes Figures S15 and S16**.
4 **Logo presentation** for Clusters A and B. Logos for clusters B2 and B3 calculated including outgroups are also shown. **Includes Figures S17 and S18**
5 **References**

Other Supplementary Materials for this manuscript include the following:

- **File S1**
- **File S3**
- **Table S1 to Table S3**

1 Legends for Other Supplementary Materials

File S1. Sequences, IDs and accession number of monomeric sHsps characterized and annotated.

(File S2. This file)

File S3. Prediction of the sub-cellular distribution. BUSCA, LocTree3, DeepLoc, TargetP-2.0, SignalP and seqNLS results.

Table S1. Species included in the analysis, accession numbers of the data and some features of the assemblies.

Table S2. Number of sequences found. Sequences containing ACDs, number of monomeric and dimeric transcripts and total ACDs and sHsps studied in this work.

Table S3. Number of sequences containing ACDs, and number of monomeric sHsps studied per species.

2 Phylogenetic trees, Predicted Secondary Structures and Multiple Sequence Alignments for Cluster A1, A2, B1, B2 and B3. Includes Supplementary Figures 1 to 14.

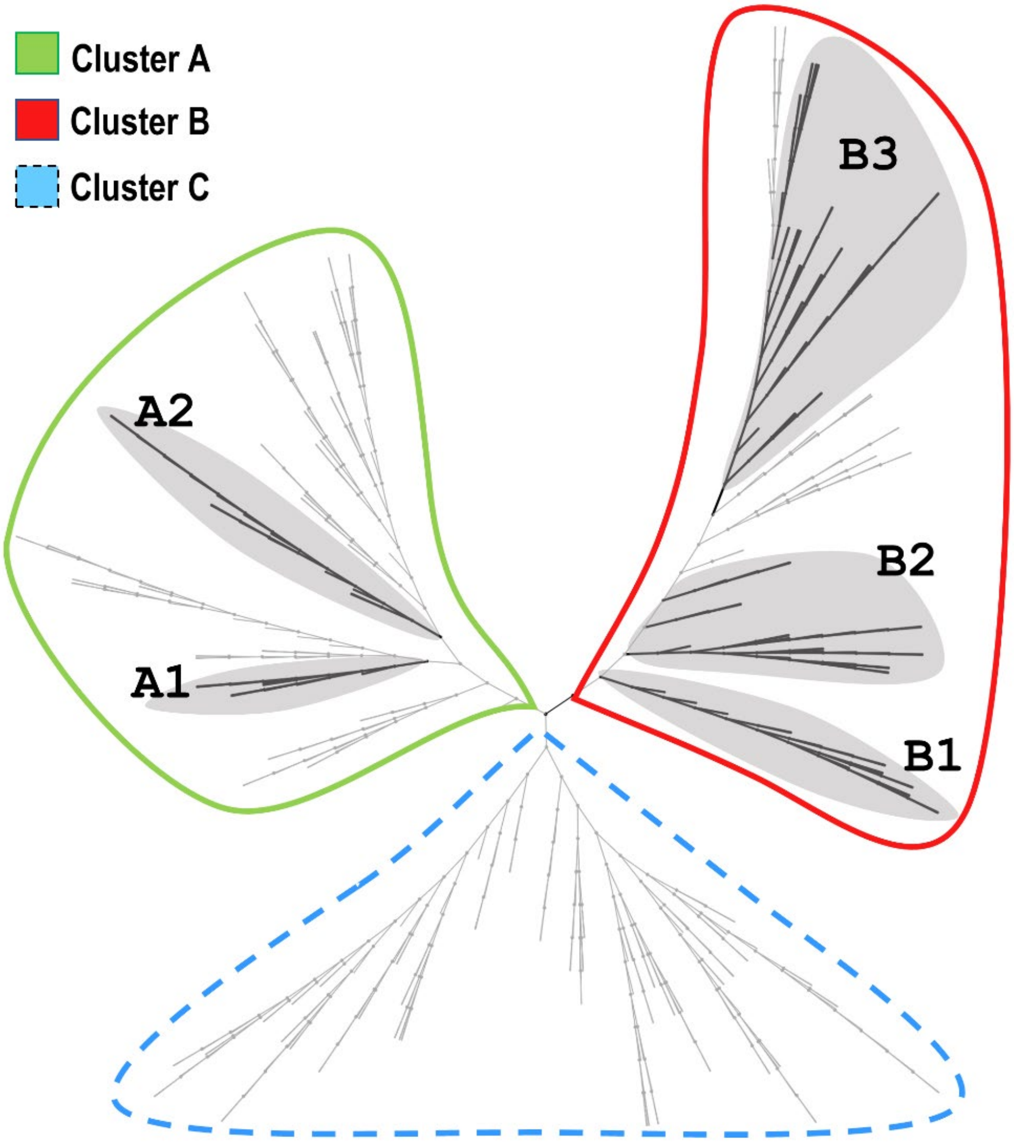

Supplementary Figure 1. Clustering branching pattern of monomeric sHsps of annelids over unrooted ML phylogenetic tree (branch lengths not displayed).

Cluster A1 – Phylogenetic trees

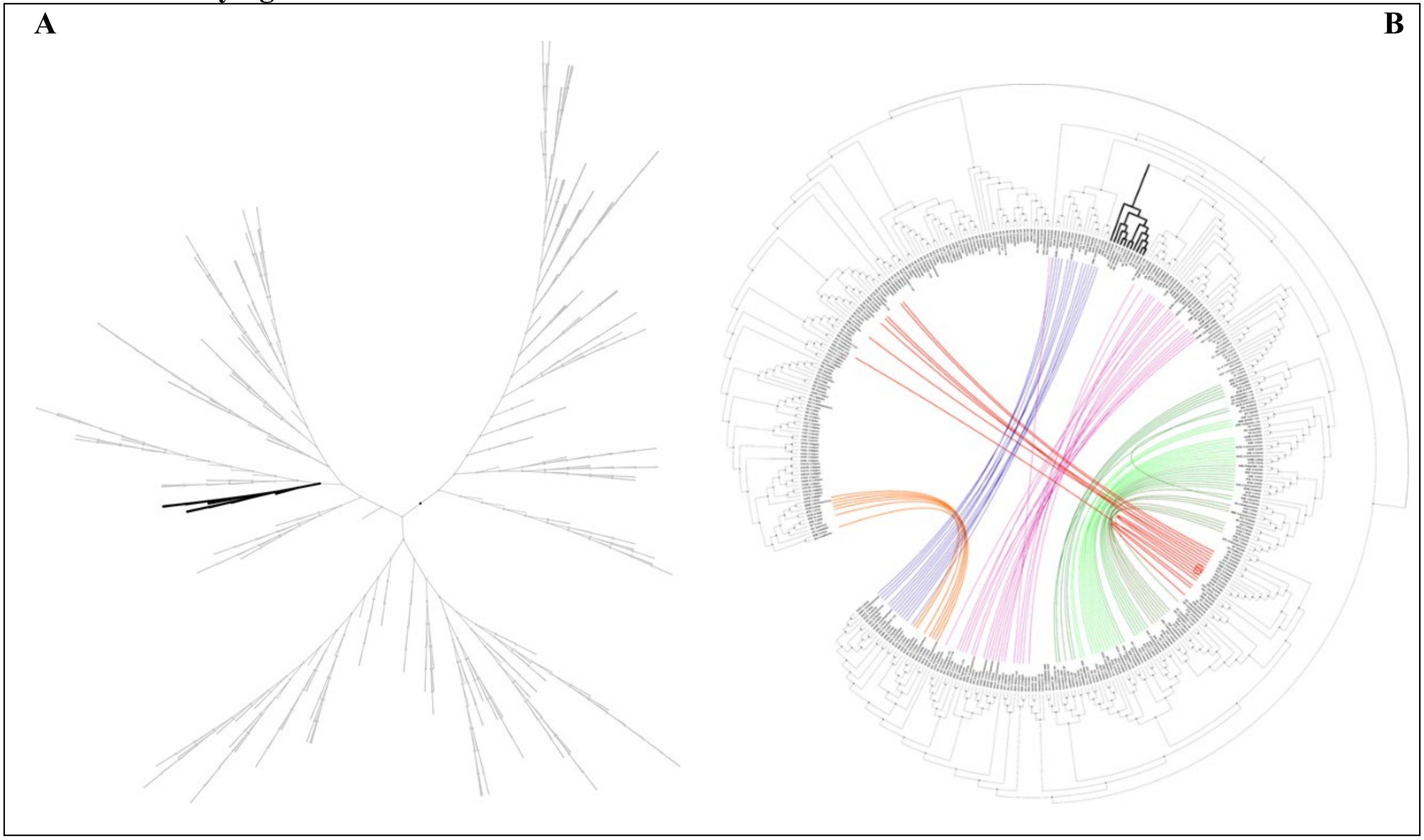

Supplementary Figure 2. A, unrooted ML phylogenetic tree, branch lengths not displayed. Small Hsp from cluster A1 in black. **B**, rerooted ML phylogenetic tree, small Hsp from cluster A1 in black and colored connection lines for two ACDs of dimeric sHsps (not covered in this work).

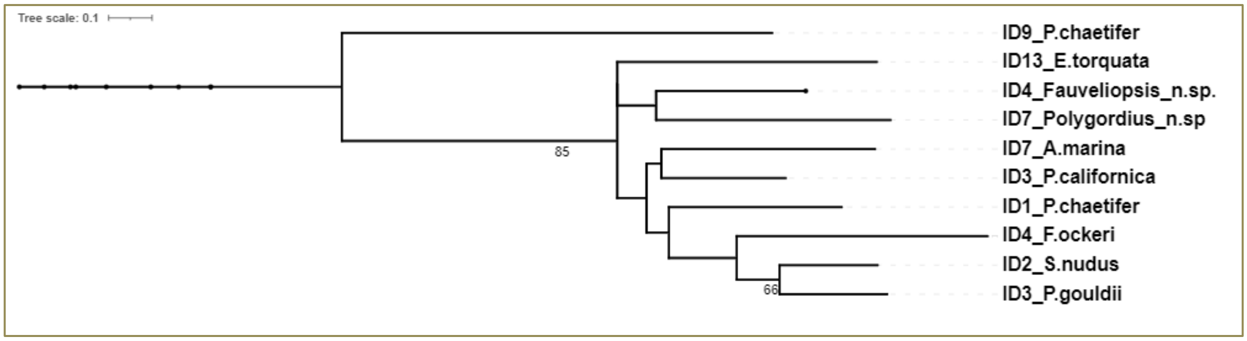

Supplementary Figure 3. Cluster A1, clade recovered by ML analysis displaying branch lengths and bootstrap >50.

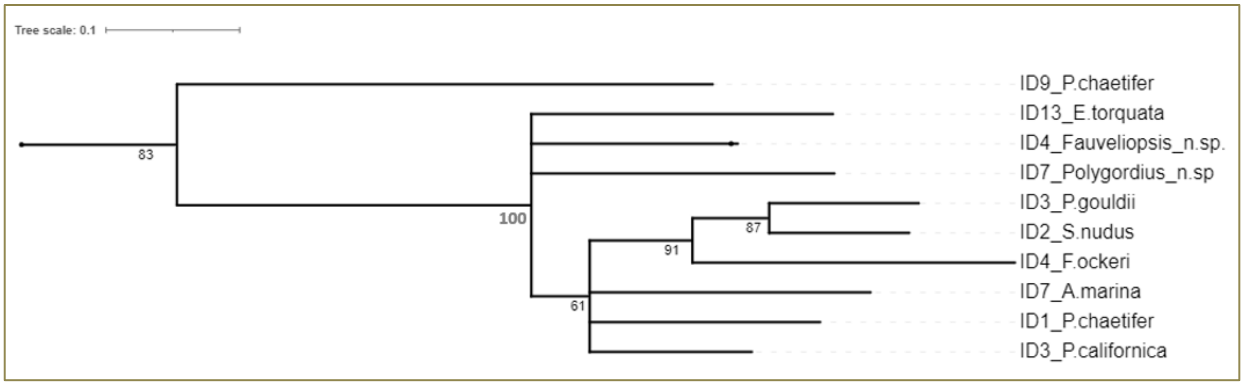

Supplementary Figure 4. Cluster A1, clade recovered by Bayesian Analysis displaying branch lengths and posterior probabilities in percentage.

Cluster A1 – Predicted Secondary Structures

Tool: PSSPRED online service (Yan et al. 2013) https://zhanglab.ccmb.med.umich.edu/PSSpred/

(**Bold black**: ACD domain delimited from the β2-strand to the β9-strand (Poulain et al. 2010); **underlined**: β3-β9 region, used in multiple sequence alignment for phylogenetic study; **H**:helix, **E**:strand, C:coil)

>ID7_A.marina
MSPQTSNVNI**KAGTPEILEEDGVRTLRITAHLIGYKDGDISVRPAEAKLLLVNKDNDVIETFNLPESVDPFTVEANISEDGMLTVMAP**LTC
CCCCCCCCCCCCCCCEEEECCCEEEEEEEEECCCCCCCCEEEEEECCEEEEEEEEEEEEEEEECCCCCCCCCEEEEECCCCEEEEEEECCC
9985346664468522785298269999952689975079999899999999742589999679999901048972699528999832 69

>ID13_E.torquata
MAPQTSD**LKEAAPVVMEKDGAKVMRLEVPLNGLSCDDVMVRPDDKRLLILHKADAALIRAFDLPESVDPFTIEAELLDGVLFVEAP**VKC
CCCCCCCCCCCCCEEEECCCCCEEEEEEECCCCCCCEEEEEEECCEEEEEEECCCCEEEEECCCCCCCCCEEEEEEECCEEEEEECCCC
99876555556643661699827999984589995306899709979999862223146664389988841489999699899982125 9

>ID4_Fauveliopsis_n.sp.
MSEH**KEKTGVEIVERDGYKVALLRVVSLPADYSADDLVVHPQDTALHILTKKQEVLRTFELPESVDPYTVEANLTDDGVLVVEAP**MMC
CCCCCCCCCEEEEEECCEEEEEEEEEECCCCCCCCEEEEEEECCEEEEEEEEEEEEEEEECCCCCCCCCEEEECCCCCEEEEEEECCC
9843357744899839948999999734678996317999979999999986445888858999992203785069952899963059

>ID4_F.ockeri
MASEFIHR**QDSVGPQVIERDGVRMLQLITQLEGFRYSDLLVRPQEDKILVMDSSEKVLKSYPLPESVDPYTVEADISDEGVLTVLAP**LKG
CCCHHHHHCCCCCCEEEECCHHHHHHHHHHHHCCCCCCEEEECCCCEEEEECCHHHHHHHCCCCCCCCCCEEEEECCCCCEEEEEEECCC
951110103566640231121589999998521243314641355528985345789986489999995035540467724899851689

>ID3_P.californica
MAPIFDETTNL**KADEPIICEVDGQRVLRLVTHLSGYKSDDVIVKPVDSKLLILTKDDVTLKSYDLPESLDPFTVEAQLGEDGVLRVEAP**VQH
CCCCCCCCCCCCCCCCCEEEECCCCEEEEEEECCCCCCCEEEEEEECCEEEEEEEEEEEEEEEECCCCCCCCCEEEEECCCCEEEEEEECCC
99864455443467872477449947999997169997328999989999999986267777628999991111898479963999984699

>ID3_P.gouldii
PVEIIEKDGQKVARLVTPLHGYAPDDITVRPAEDKLLVLDSAGAVLAEFKLPESVDPYNVEADLSEEGTLTV
CEEEEEECCCEEEEEEECCCCCCCCCEEEEEECCEEEEEECCCCEEEEECCCCCCCCCEEEECCCCCCEEEC
925786268268988630334688855999857859997157751553238999984025523568860439

>ID7_Polygordius_n.sp
MAPQITTQ**PANNARVVTGRDGTRLLQLKVALTGINQQDLAVRPVDSTLVILRNEEVLQTVELPESVDPFTVEAKMTKDGFLTVEAL**LMG
CCCCCCCCCCCCCEEEECCCCCEEEEEEEEEECCCCCEEEEEECCCEEEEEEHHHHHHHHCCCCCCCCEEEEEEECCCCEEEEEEEECC
99763334676622563578743686765412378440355414743777502777765238998994146764226631478876329

>ID1_P.chaetifer
MAPQTESSTS**NGATPVILEQDGSKFVRIQTSMVGRKLDDIIVKPMENQLVVLTKDNEILATFDLPESVDPFAVEADLSEDGVLTIEAP**LQC
CCCCCCCCCCCCCCCEEEECCCCCEEEEEEECCCCCCCEEEEEEECCEEEEEEEEEEEEEEEECCCCCCCCCEEEEECCCCEEEEEEECCC
9876444334678751674289937999995479996218999989999999974478999958999992303898379963899998526 9

>ID9_P.chaetifer
MPTL**VEIGDPLVIDDDGSQQIKLYVTVPKTVRLEDIRIKTLDETLFISAKRGMGKEAFDRTFQLPDSVDPFSVCARINDKQQLVIEAS**IMTRSRCYTL
CCCCECCCCCEEEECCCCCEEEEEEEECCCCCCCEEEEEEECCEEEEEEECCCCEEEEEEEECCCCCCCHHHHHHHCCCCCCEEEEEEECCCCCEEEE
98520147753770799827999998167778322799996776999962386213455675189999856764421689617999851123321346

>ID2_S.nudus
MAP**HVTNKSEIIEQDGGRRMRLVTSLTGYTADDISVRPTDDKVLVLDGAGAVLCAFDAPESVDPYTVEAELSENGVLTIEAP**LRS
CCCCCCCCCCEEEECCCEEEEEEEECCCCCCCEEEEEEECCEEEEEECCEEEEEEEECCCCCCCCCEEEEECCCCEEEEEEECCC
9987778861687089618999995579997417999979999999835316999985799999011389726995289998458 9

Cluster A1 – Multiple sequence alignments

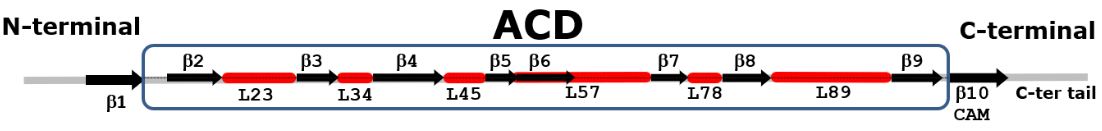

PROMALS3D alignment

PROMALS3D multiple sequence and structure alignment server http://prodata.swmed.edu/promals3d/promals3d.php (Pei et al. 2008)

```
Conservation:               955            5  6 5 99 5    55     5     95 757 6  5 5           5  5 79797996 779 5 5 7 9 6595
ID2_S.nudus              1  MAPHVTNKS--------EIIEQDGGRR--MRLVTSLTG-YTADDISVRPTDDKVLVLDGA----GAVLCAFDAPESVDPYTVEAELSENGVLTIEAPLRS---------   85
ID3_P.gouldii            1  ---------------PVEIIEKDGQKV--ARLVTPLHG-YAPDDITVRPAEDKLLVLDSA----GAVLAEFKLPESVDPYNVEADLSEEGTLTV---------------   72
ID4_F.ockeri             1  MASEFIHR--QDSVGP-QVIERDGVRM--LQLITQLEG-FRYSDLLVRPQEDKILVMDSS----EKVLKSYPLPESVDPYTVEADISDEGVLTVLAPLKG---------   90
ID7_A.marina             1  MSPQTSNV-NIKAGTP-EILEEDGVRT--LRITAHLIG-YKDGDISVRPAEAKLLLVNKD----NDVIETFNLPESVDPFTVEANISEDGMLTVMAPLTC---------   91
ID3_P.californica        1  MAPIFDETTNLKADEP-IICEVDGQRV--LRLVTHLSG-YKSDDVIVKPVDSKLLILTKD----DVTLKSYDLPESLDPFTVEAQLGEDGVLRVEAPVQH---------   92
ID1_P.chaetifer          1  MAPQTESSTSNGA-TP-VILEQDGSKF--VRIQTSMVG-RKLDDIIVKPMENQLVVLTKD----NEILATFDLPESVDPFAVEADLSEDGVLTIEAPLQC---------   91
ID4_Fauveliopsis_n.sp.   1  MSEHKEK------TGV-EIVERDGYKVALLRVVSLPAD-YSADDLVVHPQDTALHILTKK----QEVLRTFELPESVDPYTVEANLTDDGVLVVEAPMMC---------   88
ID7_Polygordius_n.sp     1  MAPQITT---QPANNARVVTGRDGTRL--LQLKVALTG-INQQDLAVRPVDSTLVILRN-----EEVLQTVELPESVDPFTVEAKMTKDGFLTVEALLMG---------   89
ID13_E.torquata          1  MAPQTSD---LKEAAP-VVMEKDGAKV--MRLEVPLNG-LSCDDVMVRPDDKRLLILHKA---DAALIRAFDLPESVDPFTIEAELL-DGVLFVEAPVKC---------   89
ID9_P.chaetifer          1  MP-TLVE-----IGDP-LVIDDDGSQQ--IKLYVTVPKTVRLEDIRIKTLDETLFISAKRGMGKEAFDRTFQLPDSVDPFSVCARINDKQQLVIEASIMTRSRCYTLTL   98
Consensus_aa:               Mtsph.p.......ss..lh-pDG.+...hpl.h.l.s.hp.pDl.V+P.-ppLhlhpp.......hl.s@pLPESVDP@oVEApls-cGhLhlbAslbs.........
Consensus_ss:                                eee     ee  eeeeee         eeeee   eeeeee      hhhheee        eeeeeee    eeeeeee
```

MEGA 7 alignment (Kumar et al. 2016)

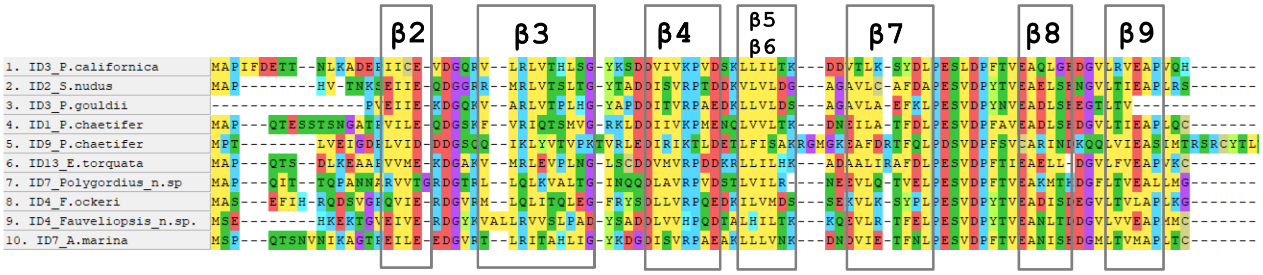

Cluster A2 – Phylogenetic trees

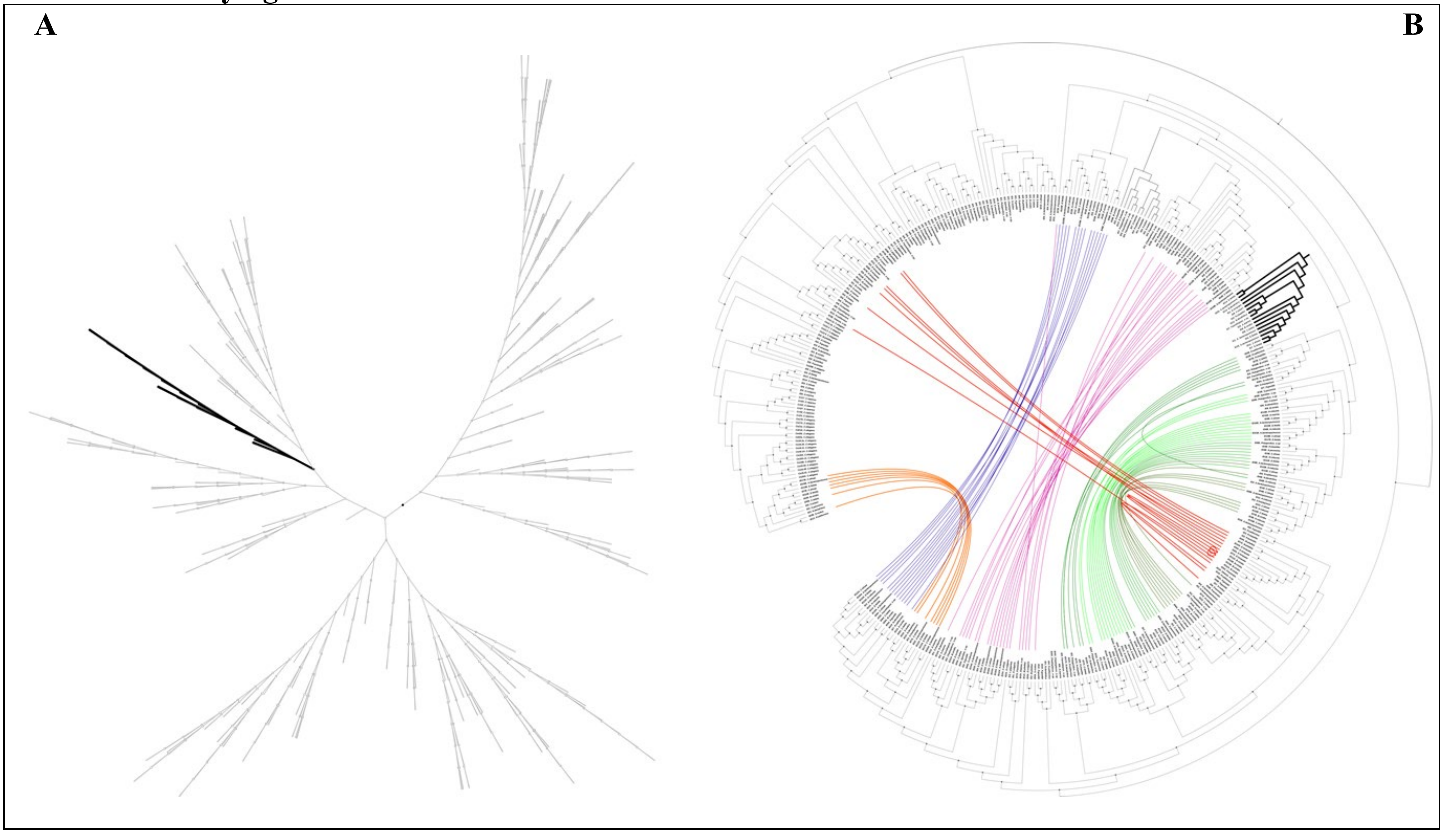

Supplementary Figure 5. A, unrooted ML phylogenetic tree, branch lengths not displayed. Small Hsp from cluster A2 in black. **B**, rerooted ML phylogenetic tree, small Hsp from cluster A2 in black and colored connection lines for two ACDs of dimeric sHsps (not covered in this work).

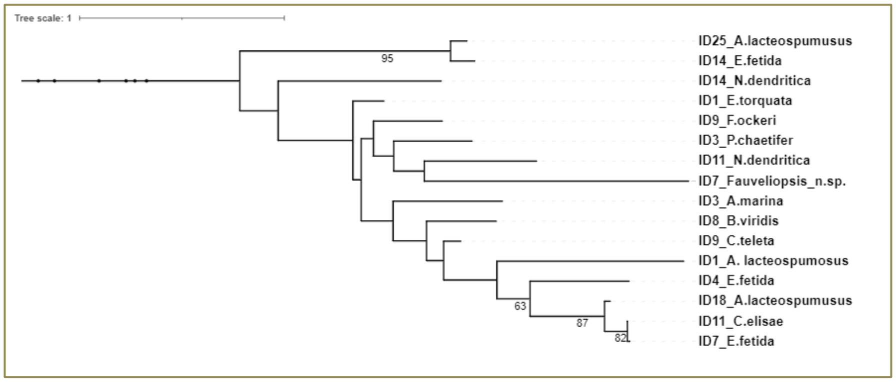

Supplementary Figure 6. Cluster A2, clade recovered by ML analysis displaying branch lengths and bootstrap >50.

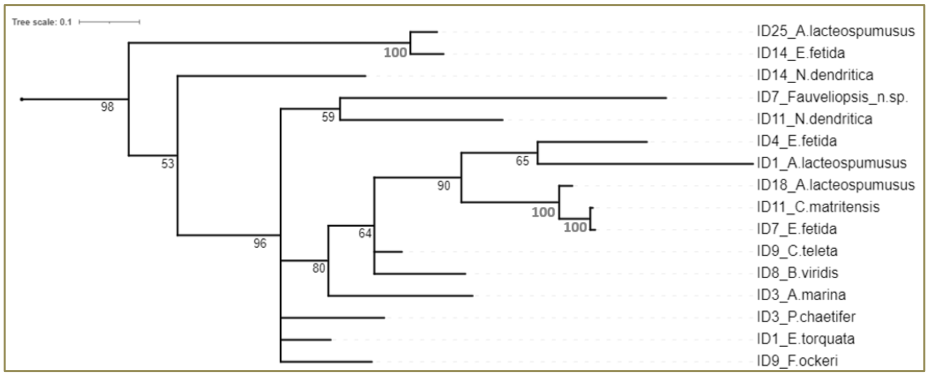

Supplementary Figure 7. Cluster A2, clade recovered by Bayesian Analysis displaying branch lengths and posterior probabilities in percentage.

Cluster A2 – Predicted Secondary Structures

Tool: PSSPRED online service (Yan et al. 2013) https://zhanglab.ccmb.med.umich.edu/PSSpred/

(**Bold black**: ACD domain delimited from the β2-strand to the β9-strand (Poulain et al. 2010); **underlined**: β3-β9 region, used in multiple sequence alignment for phylogenetic study; **H**:helix, **E**:strand, C:coil)

>ID1_A.lacteospumosus

ELHPYDLLGKVNKNGNKIRVVIAKDAGLGNGTGYSAKELISLPVQVDPYQLSARLDRDGTLHVEAPVVSLHCGARWC
CCCHHHHHCHECCCCCEEEEEEEECCCCCCCCCCHHHHHHCCCCCCCCEEEEEECCCCCEEEECCEEEEECCCCCC
9964331100127896699999502356777764334321145668900234543388607876233554214212 9

>ID18_A.lacteospumosus

MGSLMRDQRWFIMDKSSPPSPLTPFHQRQNNRSPLESPPIAKKLPKVRDGNLFFGQMFVDLSTTSSSPVFQDASEGKENRSILKRTGVKSRTLRAAGRRVDFDQDVVVNHFDR
VEGGHVSRESQRLSSSDSKDRKEFVNRTLQGRNRPITPDSHSPRISPFGSREPPIQYAVPLPAVTTQYDEPFPVTSSQYDGGRLPFDQSSFKASLTLERRVQPPAAVQERPDD
DGVMGVASEVDFAFFEDEQHRLRLKFTIALGDGVAANDVLVKANTNGNKVRLVGTRTIGTSRQGTVIRQEFTQRYQLPMEVDPYMITARMDNSGNLYVEAPVMTSDRRRALAL
ERQTISDARIGAISSDFVL
CCCCCCCCCEEEECCCCCCCCCCCHHHCCCCCCCCCCCCHHHHCCCCCCCCEEEEEEEEECCCCCCCCCCCCCCCCCHHHHHHHHHCCCCHHHHHHCCCCCCCCCCEEECCCC
CCCCCCCHHHHHHHHHHHHHHHHHHHHHHCCCCCCCCCCCCCCCCCCCCCCCCCCCCCCCCCCCCCCCCCCCCCCCCCCCCCCCCCCCCHHHHHHCCCCCCCCCCHHHHCCCCC
CCCCCCCCCCCCCCCCCCCEEEEEEEEEEECCCCCCCCEEEEEEEEECCEEEEEEEEEECCCCCCCEEEEEEEEEEECCCCCCCCCEEEEECCCCCEEEEECCCCCCCCCCCEEE
EEEECCCCCCCCCCCCCCCC
97422333024414789999998301023467877787124316755577502333454203678886322432231134666640674032232123002664302211011
23564305566655434568999886654176678888888887667665677642344455311355654445654347888998723566641553225781232026788
98521123443332257742567899998156777884389999969989999998311357874799788889857999991104898479960899980688876631344
4343154124776521369

>ID25_A.lacteospumosus

MKTSNFETVSMAAFRKSISGCLSESGNDVTDSADSTPIVKKKLCRVSDGCANSHRSLLVLKTKNDIMSENSASYTFSEGVARDIGGGDHLVEICVVRSSAVPLKGAAERILPT
GEAQHQEVDDINGNHIADSNDKEIDDRLNTEEEILSTDANSFRKRRVAFSDDVTIHSFENDDEISTDMDCDSIDDLFPDENENPQNENKNCSVSEETHCNFAKYSDLANGESN
VSSIQAVARPLYTKKMPLRRAIINQVTRACSTANLLRTFLRESFRRPNSFPHGTKTRLDRSRSLPESSIRHSFISADDHNGPVVQLVIALGQHFTPNDISVKTSGDGKKIKVL
ASQKRSGTDLIKRDYHEWFYMPKPIDPRQTRATVNYEGFLKIAAPFIA
CCCCCCCEEHHHHHHHHHHHHHCCCCCCCCCCCCCCCHHHHEECCCCCCCCCCEEEEEEECCCCCCCCCCEEECCCCEECCCCCCCEEEEEEEECCCCCCCCHHHHCCCC
CCCCHHHHHCCCCCCCCCCCCHHHHHHHCHHHHHHCCCCHHHHHHCEEECCCCEEEECCCCCCCCCCCCCCHHHCCCCCCCCCCCCCCCCCCCCCCCCCCHHHHCCCCCCCCC
CCEHHHHHCCCCCCCCCCHHHHHHHHHHHCCCHHHHHHHHHCCCCCCCCCCCCCCCCCHHHCCCCCCCCCCCCEEECCCCCCEEEEEEECCCCCCCCEEEEEEECCEEEEEEE
EEEEECCCCCEEEEEEEEEECCCCCCCCCEEEEECCCCEEEEEEECCC
98765310114566765554320368876667788851132100013565444431688740331101256411211341011588850689988523443452125540455
52100111024666445654135676521033332156024444111212661466305776544557764202237888888743345764342112212220132345444
20012321111110253048999987652022168898750124677778887632201220355334555303227998579999963568997427999979989999 96
53221599836857778984899999230489855995389996216 9

>ID3_A.marina

MGSVPDWTNRNSSSKKSSPLATSRAQQIRNPVASFLQGEGSELSRSPKDSPPTMKKKLCRLIGGIVDADFTSVDGALDALVTNGDVNGDGKHPQKWRFQRPARTPSSVQSTHP
DGSDITRTTGEVKTSKQSKPSILKKGLPSGARRRVHFDKDVTVHLLGDDGLVFESINQKLTQEAAAPQVRFRIGYTPYATRTPTEVYDSPPSPPGGGPHQGGAYHSNNNNSHH
HQHQYRYDGDDVIADKPVHVTHDTVDSSQVNFTFVEDATRGTVLKFAIPLGREYRPEDIVVKANVSGSRVRLVANRRVACGDGSMRIEQLSRRYILPMDVDPYLVGARLDIRG
NLSVEAPLLTMGKRESMKRSSHGNTSPQDY
CCCCCCCCCCCCCCCCCCCCHHHHHHHCHHHHHHCCCCCCCCCCCCCCCCHHHHHHHHHHCCCCCCCCCCHHHHHHHHHCCCCCCCCCCCCCCCCCCCCCCCCCCCCCCCC
CCCCCCCCCCCEECCCCCCCHHHHHCCCCCCCEEEEECCCEEEEEECCCCCEEHHHHHHHHHHHCCCCEEEEECCCCCCCCCCCCCCCCCCCCCCCCCCCCCCCCCCCCCCCC
CCCCCCCCCCCCCCCCCCEECCCCCCCCEEEEEEEECCCCCEEEEEEEECCCCCCCCCEEEEEECCCCEEEEEECCEEECCCCCCHHHHHHHHCCCCCCCCCCEEEEEEEEEC
CCEEECCCCCCCCCHHHHHCCCCCCCCCCC
99888754567766667886413566652046776416775445688899851357888876212356544211456666422666788888753333478888643333688
86543223331112456773255415877642145404650699844886110344567665412651345531456544787421368999999988877632357887643
22210136664202576111023456640346775135565156766404666786304775304674378874121322677422576554313567788412333443203
40222120013431112111467889877 9

>ID8_B.viridis

TVHFRDSVTVFRMAGDGSYLCTECHSLQTEPTKSPPRRTLPRNAHLKSRDTPRLPENITDRWSVSTSTYSPPTPHPDHTQCLFPPAPDLSPINFTFVEHESRGLTLKFQVPLS
SDYGPDDVVVKANMSGNRIRVVATPGGGARDEPPTFNERYPLPMDVDPYMVTARLDARGVLVVEAPVMSGSRKEELLAGN
CCCCCCCCEEEEECCCCCEEEECCCCCCCCCCCCCCCCCCCCCCCCCCCCCCCCCCCCHHHHHCCCCCCCCCCCCCCCCCCCCCCCCCCCCCCCCCCCCEEECCCCCEEEEEEEECC
CCCCCCEEEEEECCCCCEEEEEECCCCCCCCCCCCCCCEECCCCCCCCEEEEEEEECCCEEEEEEECCCCCCCCCCCCCC
94555530355423787402201222235677899732367666532356778986212220136666799999986652113688777666513405788617999972 68
7888750688621368759999615887656764334211599988932589997167169998311478642010258 9

>ID11_C.matritensis

MGSLMRDQRWFIVDKSSPPSPLIPFHQRQNNRSPLESPPVAKKLPKVKDGNIFFGQMFVDLPSASSSPVFQDAAEGKENRSILKKTGVKNRTLRTAGRRVDFEQDVVVYHFDR
VEGGHVSLESQKLSSSDSKDRKEFVNRTLQGRNRPITPDLHSPRLSSFGSREPPIQYAVPLPAATTQYTEPFPMTSHYDGCRLPFDQSSFGPNLTIERRVQQPAAVQERSDDD
GGVGVSPEVDFRFFEDEQHRLRLKFTIALGDGVAANDVLVKANTNGNKVRLVGTRTIGTSRQGTIIRQEFTERYQLPMDVDPYMITARMDNSGNLYVEAPVMTPDRKRALAIE
RQTISDARIGAISSDFVL
CCCCCCCCEEEEECCCCCCCCCCCCCCCCCCCCCCCCCCHHHCCCCCCCCCEEEEEEEECCCCCCCCCCHHHHHHCHHHHHHHHHHCCCCCHEHHCCCCEECCCCEEEEEEEE
CCCCCEEEECCCCCCCCHHHHHHHHHHHHCCCCCCCCCCCCCCCCCCCCCCCCCCCCCCCCCCCCCCCCCCCCCCCCCCCCCCCCCCCCCCCCCCCCCCCCCCCCCCCCCCC
CCEEEEECCCCCCCCCCCCCCCCCEEEEECCCCCCCCCEEEEEEECCEEEEEEEEEEECCCCCCEEEEEEEEEEECCCCCCCCEEEEEECCCCCEEEEEECCCCCCCCEEEEE
EECCCCCCCCCCCCCCCC
97423453147750689999876521134567877788213216765677424444432067888885111133110124666641676300011033021102237886432
13640443111244332035899988764256777887778886544567788620035577776555677775445787678877776678865565335654322223578
83247640135556773113333214542245567752389999699899999995312688867998899998579999932048984799608999806899886203346
632565334555555359

>ID9_C.teleta

MGTLIRPPNNSNKRWPEPKALVDSTNRQLNHSPVDSPPTVKKKLCRLAKGLFADSSSAATHRGALASLDAAFVSDAPAVELAQIALGSVPASSLRRLERCNSLTTKGASILKK
GKLLRASIRAKSHSWPPHKSVKFHDDVTVIRINDDGEYVCTECAQLQAAATQPDLLPGHSVRDRQLYMLRSTPQRLMRRSQSPPAVREPHAAQVNAPTTEANAKQQTEKEEPW
SPDHQANPATSASSTSTTT**NGVNFSFVEDPLHGL<u>KLKFSVPLGAGYGPSDIVVKANMSGNRIRVVASRSSGAGHRSSFEQFNERYPLPMDVDPYMVTARLDVKGQLIVEAP</u>**LM
TLARRNSFDNGRQKTAQAAEQAFGAV
CCCCCCCCCCCCCCCCCCCCCCCCHHHCCCCCCCCCCCHHHHHHHHHHHHHHCCCCCCHHHHHHHHHHHHHHCCCCCHHHHHHHHHCCCCHHHHHHHHHHHHHHHHHHHHHHH
HHHHHHHHHHHCCCCCCCCCCCCCCCEEEEEECCHHHHHHHHHHHHHHHCCCCCCCCCCCHHHHHHHHHHHHHHHHHHHCCCCCCCCCCHHHHCCCCCCCCCCCCCCCCCCCC
CCCCCCCCCCCCCCCCCCCCCCCCCEEECCCCCCEEEEEEECCCCCCCCEEEEEEEEECCEEEEEEEEEECCCCCEEEEEEEEEEECCCCCCCCEEEEEECCCCEEEEEECCC
CCCCCCCCCCCCCCCCCHHHHCCCCC
98533789988888888621112201102689899995047899999875314665312322123431022115674246777754047657899999864310001233343
32212221230378998864311244046653230135675688875212465557874145788886535567888641578643330222113654334300023455688
97412477644333332236777642442899727999997588999524899999869899999999851799727867988994899999331699976997699997278
75312455544320011122102589

>ID4_E.fetida

LVFHFDRNEGGHVCLESQVLSVEPEVKNVLRPSNAFSSTTSSTNSTTSVSSPLSRQSDTIKNRLESSNGGTNLHPAMRGTSTPQQRVLATQKHSEVDFQT**QNVQFHFAVNECR**
TS<u>VLRFSLPLGAGTSAGDVIVKANKVGNRVRILGAENCSREINERFGLPVRVDPYRISARLDSNGMLFVEAP</u>ILSGEQKNGLLSGRKSL
CCCCCCCCCCCCEEEECEEEECCCHHHCCCCCCCCCCCCCCCCCCCCCCCCCCCCCHHHHHHHHHCCCCCCCCCCCCCCCCCCCHHHHHHHCCCCCCEEEEEEEEEEEECCCC
CEEEEEEEECCCCCCCCEEEEEEECCCCEEEEEECCCHHHHHHHHHCCCCECCCEEEEEEECCCCEEEEEECCCCCCCCCCCCCCCCCC
93322367775045410133126203203467666555556667642113665431146777763158986544433578984011012212431000121267876420122
11466654136777765279863003633788612212577887518752026314566634885178651013553234422466679

>ID7_E.fetida

FGSREPPIQYAVPLPTMTTLYPEPYAVTSQYDGMRLPFDQSSFGPNATLERRTQPTGPVLERPDDDGGMGVA**PDVDFTFFEDEQHRLRL<u>KFTIALGDGVAANDVLVKANTNGN</u>**
<u>KVRLVGTRTIGTSRQGTIIRQEFTERYQLPMDVDPYMITARMDNNGNLYVEAP</u>VMTPDRKRALAIERQTISDARIGAISNDFVL
CCCCCCCCCCCCCCCCCCCCCCCCCCCCCCCCCCCCCCCCCCCCCCCCCCCCCCCCCHHHCCCCCCCCCCCCCCCCCCCCCCCCCEEEEEEEEECCCCCCCCCEEEEEEEECC
EEEEEEEEEEEECCCCCEEEEEEEEEEECCCCCCCCCEEEEECCCCEEEEEEECCCCCCCCEEEEEEECCCCCCCCCCCCCCCC
98777863112354011146753223333334333467765566654222345664023214677765444554555433687412356899852556678862799998699
899999974420589867999999999689999922127871899628999826899986125667613664113555556679

>ID14_E.fetida

MKTSNIETSNMASFTKSTSGCLPERSNDVKDSDSPPVVKKKLCRVSNGRANTHRSLLVLSTKKDTVSEKRASYTFSEGIAREIGGGDHLVEICVVRGSVPPCQHLAEAGQHTE
DSQQHGVKDRNGNLLENETDGRIRRRPADSDEELSSNDAKSPTKRRVAFSDDVTIHNFETEDDISSDMDCASVDENFPDDNLRNENKNHVLSAETNDDTNAPLTHTIALPFYS
KKMPLRRVILNQVTRACSTANLLRTLLRESFRKPNSNQHGARTPLARSRSLPE**SSVRHSFIGADDNNGA<u>AVQLVIALGQHFAPNNISVKTSGDGKKIKVLASLKRSATDHVKW</u>**
<u>DYQEWFYLPKPIDPRETKATVDYEGFLKIEAP</u>FIVSSPLNSWTQ
CCCCCCCCCCCCCCCCCCCCCCCCCCCCCCCCCCCCCHHHHEEECCCCCCCCCCEEEEEECCCCCCCCCCCCCCCCHHHHHHCCCCCCEEEEEEECCCCCCCHHHHHHCCCCC
CHHHCCCCCCCCCCCCCCCCCCEECCCCCCHHHHCCCCCCCCCCCEEECCCCCEEEECCCCCCCCCCCCCCCCCCCCCCCCCCCCCCCCEEECCCCCCCCCCCEEEEEECCCC
CCCCHHHHHHHHHHHHHHHHHHHHHHHHHHHCCCCCCCCCCCCCCCCCCCCCCCCCCCEECCCCCCCCCEEHHHHHHHHCCCCCCEEEEECCCCCEEEEHHHHHCCCCCCCEE
CHHHHHCCCCCCCCCCCCEEECCCCEEEEECCEEECCCCCCCCC
98655444331112346676666544556667898510210000135765542215775147653101012211210133430576524788764068886167766313442
01220433467862124445531127887323211245678632101113651354213545543345543344468863234345553131135788887521233302344
56642578888776544467999999999732677655665554312356763211100113468886100113554322577633664147872354213431112333100
04442126888984334112211112342033120366543479

>ID1_E.torquata

PGKGKVGVGATVSHSAPGKPGNYAVTVSTSGLASDERKRESSAPSPIPNKTGARQVHGNSSNNMSHNSITSSNDSSSNTNKSATVP**GSVNISFAQDPTHGT<u>KLKFVVNIGQNY</u>**
<u>DPEDIVVKANMNGNRIRVMACKVVARVDGSRDMQQFNERFVLPMDIDPFTVDARLDTRGLLTVEAP</u>LITLDRRPDSNHSPDPPTTTV
CCCCCCCCCCCCCCCCCCCCCCEEEECCCCCCCCCHHHHHCCCCCCCCCCCCCHHHHCCCCCCCCCCCCCCCCCCCCCCCCCCCCCCCCCCCCEEEECCCCEEEEEEECCCCC
CCCEEEEEEEECCCEEEEEEEEEEEECCCCEEEEEEEEEEECCCCCCCCCEEEEECCCCEEEEEEECCCCCCCCCCCCCCCCCCCCC
98765312311234577666530221255543321222211344322100011122111112344444455666666432234554455764112102697389999828999
95138999998199899999998751389559999999995899999111189847997499997068776677766677787899

>ID7_Fauveliopsis_n.sp.

PLNVSTVAPLPKQNQNKQLPESQTSGSLPS**KVASISFVQDDYSHCT<u>KLKCVIYLGIDYDPNNVVVKANMNGSRIRVVANRVVEESEGVTAFETFNERYVLPMDVDPYAVEARF</u>**
<u>DIRGNLTVEAP</u>LMTTSRRESMKAPQHISQSVTV
CCCCCCCCCCCCCCCCCCCCHHHHCCCCCCCCCCEEEEECCCCCCCEEEEEEEEEECCCCCEEEEEEECCCCEEEEEEEEEEECCCCCCEEEEECEEEECCCCCCHHHEEEEC
CCCCCEEEEEECCCCCCCCCCCCCCCCCCCCCC
98733345565323311264133035676542100134125555421468998631368761799985488517999622210235641112221156257999902334410
257526998301235311123563111355569

>ID9_F.ockeri

MGTLIQEMPPTILKVHDAHSRPLTIDTATANLRAPPIGHLSHSPLESPPILKKRLRRLGSSLLAGTLNVRDLPPELMAVLKHQDSSPQTTRSLLSQNGRSKMAGSNTLARASS
CPPRHRVNSMSSSQEKAGSILKGDTRRSLRVPSSPGKRVQFESNVTVHLMDEEGHICTQCEQLQIFPRQSRRDFIARNIRRQRNFPAPSHNSPSPSTPTTDFHTNNIYRRGDG
DQDLSPDMNMSSNVYTGPRPVLNQDNVNP**SNINITFEQDADKGT<u>KLKFVVYLGLDYQPENVVVKANMNGNRIRVLATVEQSGEEFNQRYLLPMNVDPFSVEARLDTKGYLTVV</u>**
<u>AP</u>LMTNARRQELSHKDKPAAS
CCCHHHHCCCCEEEECCCCCCCCCECCCCCCCCCCCCCCCCCCCCCCCHHHHHHHHHHHHHHHHCCCCCCCCCHHHHHHHHHCCCCCHHHHHHHHHC
CCCCCCCCCHHHHCCCCCCCCCCCCCCCCHHHHHHHCCCCCCCCCCCCCCCCCEEEEECCEEEEEECCCCCHHHHHHHHHHCCCCCCCCCHHHHHHH
CCCCCCCCCCCCCCCCCCCCCCCCCCCCCCCCCCCCCCCCCCCCCCCCCCCCCCCCCCCCCCCCCCCEEECCCCCCEEEEEEEECCCCCCCEEEEEEEECCCEEEEEEEEEEC
CCEEEEEEECCCCCCCCEEEEEECCCCEEEEEEECCCCCCCCCCCCCCCCCCC
96213217862223214677763100121224689876667888888647899999985665420234556887999999605898546889986315311234201110137
99731134443102443100147765310278998832776512489985478722445666642111122341233221002589988889999998765567765566777
64456655677664567887655557886555651565168882699999930367873389999953998999999730232267898669999944279996699618999
825787654445546888889

>ID11_N.dendritica

MPPDANLLNSKVGISSTFRDRSNHIAHAAPSTYMLPTFEYPDLLKSDNKNTDSVHRLSPQAASKKRPRSGTLDSPMLGKENLSSASDSSADCDSVFNYPSSSPRGSILKGKRK
KMRGSISPKDSTCARNVQFRPAVTVFQMNDEGDHVCTKCENLKDEPAVSSAPRIRSFRNGHAPLSALCKDSTLLQPSSPRFAGASRTNMNIPIPDYSSASHAHNNYVIRHRGA
ATVAHARSDFADSAAAAAAAAPHSGSKDESCHGQISNSMPVCNGSHQIPAI**GSMSISFSTDPSRATILKMSVYIGHGYTADDIVVKAGVNGSQVRVIANKFAPHPTGQMNHVQ**
QFNEKYVLPMSVDPYSIDARVDNHGFLTVEAALMTLNRKETVLTQG
CCCCCCCCCCCCCCCCCCCCCCCCCCCCCCCCCCCCCCCCHHHHHCCCCCCCCCCCCCCCCCCCCCCCCCCCCCCCCCCCCCCCCCCCCCCCCCCCCCCCCCCCCCCCCCCCCCC
CCCCCCCCCCCCCCCCEEECCCEEEEEECCCCCCHHHHHCCCCCCCCCCCCCCHHHCCCCCCCHHHHCCCCCCCCCCCCCCCCCCCCCCCCCCCCCCCCCCCCCCEEEEECCC
CHHHHHHHHHHHHHHHHHHHCCCCCCCCCCCCCCCCCCCCCCCCCCCCCCCCEEEEEECCCCCCCEEEEEEEEEECCCCCCCEEEEECCCCCEEEEEEECCCCCCCCCHHHHH
HHHHHCCCCCCCCCCEEEEEECCCCEEEEHHHHHHHCCCCEEECCC
99861011244333322135542000257763113554541232036787532101473200136788787788756643444345566653211358899988620135422
11135677766532110102312566414777620221001366765356852111256887434421566434789975455545545678887665544444213420242
01121100012245555530567788765533223566643467654443111356532788741356667774035554404664246773479975113678776314566
5321000234568630112213653033024444303442010379

>ID14_N.dendritica

MKPYKRSFSSLARLTFLGKKSDKGSKKGEDGKTDTDIATDQETNRKPDVKIKVSPSTPTQKQKPYKKSSSISFFSSMLKKGDDNAQVSRDVDREEDVADLIPVPVTRKTSPLM
PDPSQEEREPVTVKTVNGSIPGSSNLMPHTLPRARRTQAGLELNGIERVDSEQSSLPDLVDDYDEDEEDDDDEYDDETASGVTSLLPPILRKRSKKSATRPAPNKEGSSERRA
HFREQVEVLQQDAEGNPLRVEVELKQGISFGGHFGLNKDKAKKA**KSLRHIFPSEDIVVIKQRNGKKKLRLVVHIGSGFSSDKIAVRALKGGKKLHVTAEKAEQMADGSDFWRE**
FTDKFVLPHKINPYKVKASFDIIGDLCIEAPFAESLF
CCCCCCCCHHHHEEEEECCCCCCCCCCCCCCCCCCCCCCCCCCCCCCCEEEEECCCCCCCCCCCCCCCCCCHHHHHHHHCCCCCCCCCCCCCCHHHHHHCCCCCCCCCCCCCC
CCCCCCCCCCCEEEEECCCCCCCCCCCCCCCCCHHHHHHCCECCCCECCCCCCCCCCHHHHHCCCCCCCCCHHHHHHHHHHHHHHHHHHHHHHCCCCCCCCCCCCCCCHHHHH
HHHHHHHHHHHCCCCCCEEEEEEEECCCCCCCCCCCCCHHHHHHHHHHCCCCCCCEEEEEECCCCCEEEEEEEECCCCCCCCEEEEEECCCCEEEEEHHHHHHHHCCHHHHHH
HHCCCCCCCCCCCCEEEEEEECHHHHECCCCCCCCCC
98632330100003441356666544455676532222222246787514661588886556665556530368887521575320112332023332113455456788889
99875457763145513667887777776564122212022002410124444432023210344433441111111010123212155543113456778875567514467
77888999863157985368998513421244334563123334432113577754898624777327899983478877604666413773355315667764032256776
5201124555775302211000000001354420049

>ID3_P.chaetifer

MAATVSSVPLRDRSNVMSDYKTVLSEPQYFDFQSTEKLLNKAIGIPINIERSPRDSPDIFKKKPRTQTPLSPSDRVKAIIKETARAVQEVCPPLSAAAKKTILKPSRCESPAR
TERRKVQFNHNVAVCLIDAHGDRVCMTNENLNTYEGGSDNPMYTRLKRSKSFTPLTYSQKPTTNDSTVVKGQGHTTESLRSSPHVDPPIPTGIIHQSSVSNE**SKINISFASDR**
VSGTKLKFVLYIGPQYNANDVVVKACTNGSKLRVVASTTVHKDGVTSLSQFNERFTMPMEVDPFAVDAKMDNRGYLTVEAPLMTTERRERVRRRLPRGTDV
CCCCCCCCCCCCCCCCCCHHHHCCCCCCCCCCHHHHHHHHHHHCCCCCCCCCCCCCHHHHHCCCCCCCCCCHHHHHHHHHHHHHHHHHHHCCCCCHHHHHCCCCCCCCCCCCH
HHHHEEECCCCEEEEEEECCCCCEEECCCCCCCCCCCCCCCHHHHHHCCCCCCCCCCCCCCCCCCCEEEECCCCCCHHHCCCCCCCCCCCCCCCCCCCCCCCCEEEEEEECCC
CCCCEEEEEEEECCCCCCCCEEEEEECCCCEEEEEEEEEECCCCCCCHHHHHHHCCCCCCCCCCEEEECCCCCCEEEEEECCCCHHHHHHHHHHCCCCCCC
97532345555443211101101379753353047899887526544446899987144313777789998004689999999999987353421123330346445678521
12111220343378986057871342146754336899983022221257766421136777777415513566402110389989988753100134676522566640244
45624789999535678762488751589538998743321466530676542214666567411111025752167651112135778888615787789

Cluster A2 – Multiple sequence alignments

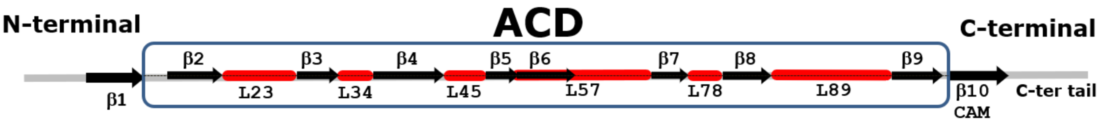

PROMALS3D alignment

PROMALS3D multiple sequence and structure alignment server http://prodata.swmed.edu/promals3d/promals3d.php (Pei et al. 2008)

```
Conservation:                      5  5 8   5      75  5 78          5 665   955566 6                5 5 5 79  7795   9  8  9 9 7698
ID3_P.chaetifer            1   VSNESKI-NISFAS-DRVSGTKLKFVLYIG--PQYNANDVVVKACTNGSKLRVVASTTVHKDG--VTSLSQFNERFTMPMEVDPFAVDAKMDNRGYLTVEAP   96
ID1_E.torquata             1   ATVPGSV-NISFAQ-DPTHGTKLKFVVNIG--QNYDPEDIVVKANMNGNRIRVMACKVVARVD-GSRDMQQFNERFVLPMDIDPFTVDARLDTRGLLTVEAP   97
ID9_F.ockeri               1   NVNPSNI-NITFEQ-DADKGTKLKFVVYLG--LDYQPENVVVKANMNGNRIRVLA--TVEQSG------EEFNQRYLLPMNVDPFSVEARLDTKGYLTVVAP   90
ID7_Fauveliopsis_n.sp.     1   GSLPSKVASISFVQDDYSHCTKLKCVIYLG--IDYDPNNVVVKANMNGSRIRVVANRVVEESE-GVTAFETFNERYVLPMDVDPYAVEARFDIRGNLTVEAP   99
ID11_N.dendritica          1   IPAIGSM-SISFST-DPSRATILKMSVYIG--HGYTADDIVVKAGVNGSQVRVIANKFAPHPTGQMNHVQQFNEKYVLPMSVDPYSIDARVDNHGFLTVEAA   98
ID4_E.fetida               1   DFQTQNV-QFHFAV-NECRTSVLRFSLPLG--AGTSAGDVIVKANKVGNRVRILGAEN---------CSREINERFGLPVRVDPYRISARLDSNGMLFVEAP   89
ID9_C.teleta               1   STTTNGV-NFSFVE-DPLHGLKLKFSVPLG--AGYGPSDIVVKANMSGNRIRVVASRSSGAGH--RSSFEQFNERYPLPMDVDPYMVTARLDVKGQLIVEAP   96
ID8_B.viridis              1   APDLSPI-NFTFVE-HESRGLTLKFQVPLS--SDYGPDDVVVKANMSGNRIRVVA--TPGGGA--RDEPPTFNERYPLPMDVDPYMVTARLDARGVLVVEAP   94
ID3_A.marina               1   TVDSSQV-NFTFVE-DATRGTVLKFAIPLG--REYRPEDIVVKANVSGSRVRLVANRRVACGD-GSMRIEQLSRRYILPMDVDPYLVGARLDIRGNLSVEAP   97
ID11_C.matritensis         1   VGVSPEV-DFRFFE-DEQHRLRLKFTIALGDGVAANDVLVKANTN--GNKVRLVGTRTIGTSRQGTIIRQEFTERYQLPMDVDPYMITARMDNSGNLYVEAP   98
ID7_E.fetida               1   MGVAPDV-DFTFFE-DEQHRLRLKFTIALGDGVAANDVLVKANTN--GNKVRLVGTRTIGTSRQGTIIRQEFTERYQLPMDVDPYMITARMDNNGNLYVEAP   98
ID18_A.lacteospumosus      1   MGVASEV-DFAFFE-DEQHRLRLKFTIALGDGVAANDVLVKANTN--GNKVRLVGTRTIGTSRQGTVIRQEFTQRYQLPMEVDPYMITARMDNSGNLYVEAP   98
ID1_A.lacteospumosus       1   ---------------------------------ELHPYDLLGKVNKNGNKIRVVIAKDAGLGNGTGYSAKE---LISLPVQVDPYQLSARLDRDGTLHVEAP   66
ID14_N.dendritica          1   IFPSE---DIVVIK-QRNGKKKLRLVVHIG--SGFSSDKIAVRALKGGKKLHVTAEKAEQMAD-GSDFWREFTDKFVLPHKINPYKVKASFDIIGDLCIEAP   95
ID14_E.fetida              1   SLPESSV-RHSFIGADDNNGAAVQLVIALG--QHFAPNNISVKTSGDGKKIKVLASLKRSATD---HVKWDYQEWFYLPKPIDPRETKATVDYEGFLKIEAP   96
ID25_A.lacteospumosus      1   SLPESSI-RHSFISADDHNGPVVQLVIALG--QHFTPNDISVKTSGDGKKIKVLASQKRSGTD---LIKRDYHEWFYMPKPIDPRQTRATVNYEGFLKIAAP   96
Consensus_aa:                  ..s..pl.phsFhp.s..p...L+hsl.lG....hps.pl.h+hs.sGp+l+lltsp....sp......pp@s-.@.LPhplDP@.lpAphD.pG.L.lEAP
Consensus_ss:                           eeeee       eeeeeeeee         eeeeeee    eeeeeee   e       ehhh   eee       hhheeeee    eeeee
```

MEGA 7 alignment (Kumar et al. 2016)

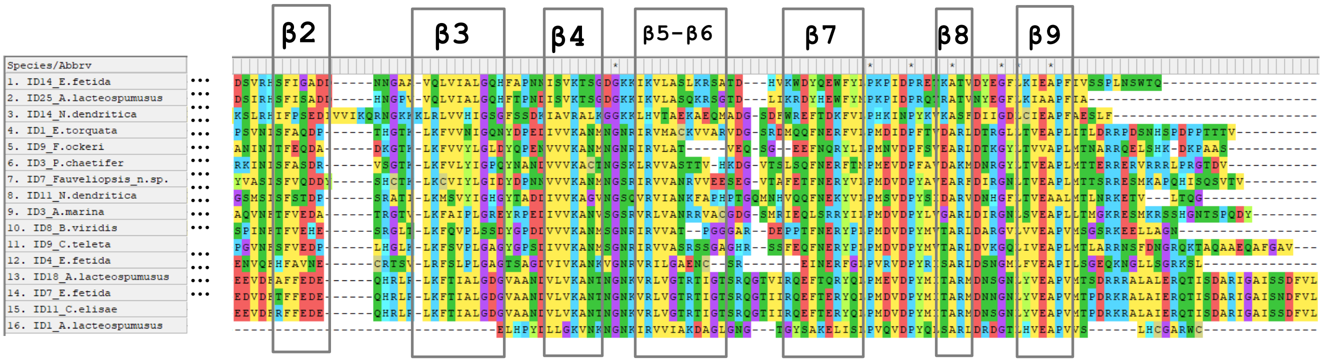

Cluster B1 – Phylogenetic trees

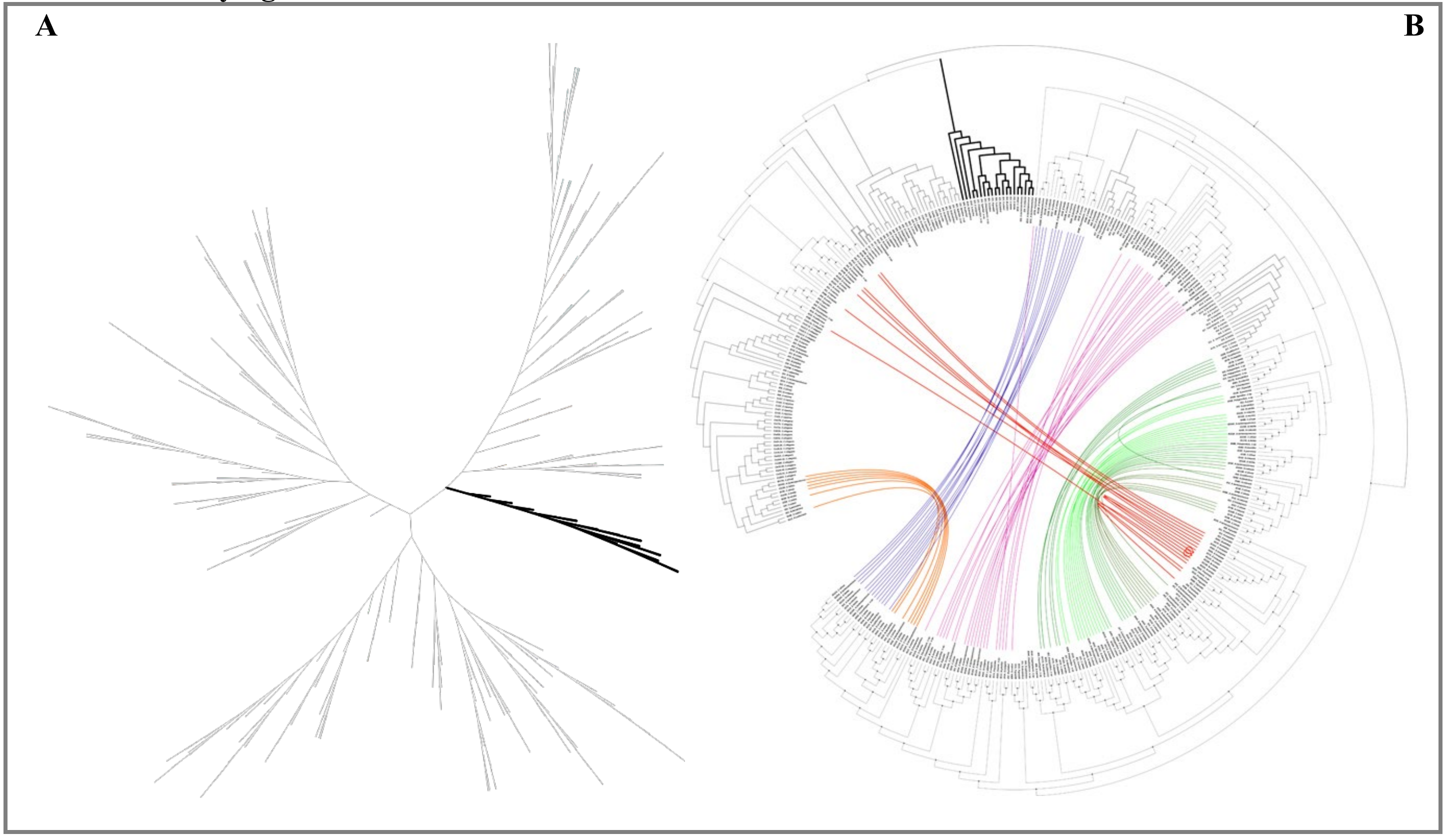

Supplementary Figure 8. A, unrooted ML phylogenetic tree, branch lengths not displayed. Small Hsp from cluster B1 in black. **B**, rerooted ML phylogenetic tree, small Hsp from cluster B1 in black and colored connection lines for the two corresponding ACDs of dimeric sHsps (not covered in this work).

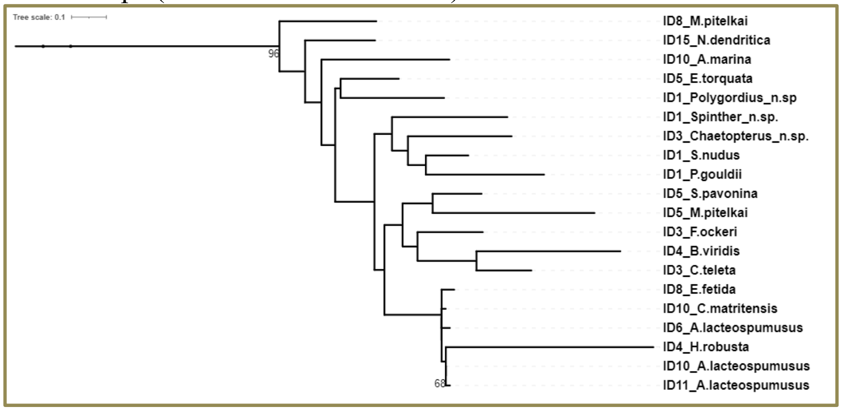

Supplementary Figure 9. Cluster B1, clade recovered by ML analysis displaying branch lengths and bootstrap >50.

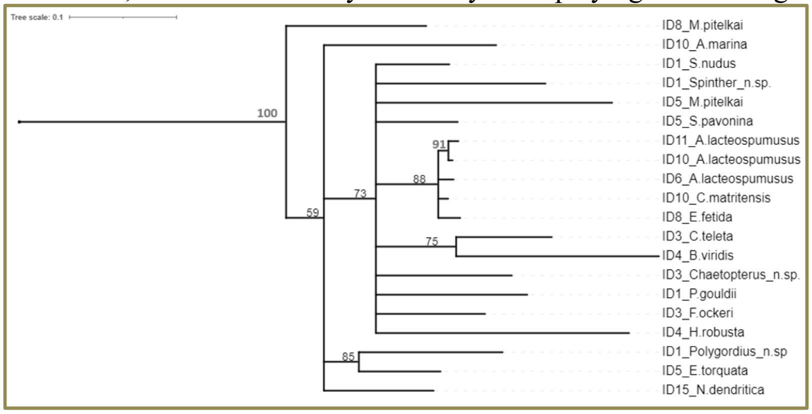

Supplementary Figure 10. Cluster B1, clade recovered by Bayesian Analysis displaying branch lengths and posterior probabilities in percentage.

Cluster B1 – Predicted Secondary Structures

Tool: PSSPRED online service (Yan et al. 2013) https://zhanglab.ccmb.med.umich.edu/PSSpred/

(**Bold black**: ACD domain delimited from the β2-strand to the β9-strand (Poulain et al. 2010); **underlined**: β3-β9 region, used in multiple sequence alignment for phylogenetic study; **H**:helix, **E**:strand, C:coil)

>ID6_A.lacteospumosus

MPISSPDIEKSLVRLDSDSLRSCIDKAHGDRLKLNFDVNEFESESINIKTVGNKIEVHAKKKSKKGDEERSEEFSRVYELPTQNAVDSGNVSSSIYKDGVLTIELPIADAIAG
CCCCCCCCCHHHHCCCCCCCCCCEEEECCCEEEEEEECCCCCCEEEEEEECCEEEEEEEEEEECCCCEEEEEEEEEEECCCCCCCCHHHEEEEECCCCEEEEEEECCCCCCC
99998755314422455567753146529859999980899953189999899999999987422687178999999976899999932168985499728999807877789

>ID10_A.lacteospumosus

MSELIPQGQKSAATSASSSYAFSSTSSSQSSSSSSSKVVKQSASTVKSSSTVSSSSSLSSSATSSSKAVGIKSSGGSYLDRMASDFGIDSGKEVEKLKLKMKEEFSNIHQDMF
QLIPINSPEAHKTVVLLDSDSLKSCIDKAHGDRLKLNFDVNEFESESINIKTVGNKIEVHAKKKSVKGDEERSEEFSRVYELPTQNAVDAGNVTSSIYKDGVLTIELPVADAI
AGTDVLLSQ
CCCCCCCCCCCCCCCCCCCCCCCCCCCCCCCCCCCCCCCCCCCCCCCCCCCCCCCCCCCCCCCCEECCCCCCCCCCCCCCCCCCHCHHHHHHHHHHHHHHHHHHHHHHHHHH
CCCCCCCCCCCCCCCCCCCCCCCCCEEEECCCEEEEEEECCCCCCEEEEEEECCEEEEEEEEEEECCCCEEEEEEEEEEECCCCCCCCHHHEEEEECCCCEEEEEEECCCCC
CCCCCCCCC
951011134445788887754223543357765454333310014566656677522233456675200233588875654356530011125788998999987754233 2
0256777532344333444556522452498499999818999530899998999999999852126862788999999758998999211589855996289998368888
776446899

>ID11_A.lacteospumosus

MGKEMEQLKLKMSEEMSNIHKDIFQLMPISSPDIEKSVVRLDSEALKSCIDKAHGDRLKLHFDVNEFESESINIKTVGNKIEVHAKKKSVKGDEERSEEFSRVYELPTQNAVD
AGNVTSSIYKDGVLTIELPVADAIAGTDVLLSQ
CCCHHHHHHHHHHHHHHHHCCCCCCCCCCCCCCCCCCCCCCCCCCCCCCEEEECCCEEEEEEECCCCCCEEEEEEECCEEEEEEEEEEECCCCEEEEEEEEEEECCCCCCCC
HHHEEEEECCCCEEEEEEECCCCCCCCCCCCC
974235455578889886311000346787754454310133345675236751984999998079995318999989999999997420268638999999998589999 99
43348874499718999817888887654578 9

>ID10_A.marina

MPRLLPKVSEKDCWLDLMGSLHTDSTSTARQNLFHLTPLQPMGSGSEIIKLDNTSILDYIDKDDRDLLKFNFDVSGYQSQTVIVKSDGNKIEVFAKKTSKVGDEERSEEFSRS
YEMPRDGAIDASKITSSFYKDGILSVE
CCCCCCCCCCCCCCCCCCHHHCCHHHHHHHHCHHHCCCCCCCCCCCCCCCCCCCCCCCCECCCCCCEEEEEEECCCCCCCEEEEEEECCEEEEEEEEEEECCCCEEEEEEEEE
EECCCCCCCCHHHEEEEECCCCEEEEE
99755567654543344201100156776401221243345666642011257876420014799549999973899973189999899999999998633796799999999
9768888999322588846997189 97

>ID4_B.viridis

MSEAEGQSKFTSSIVISTGASTVVAPMTTKMTTSKSSMTKTTKIIKSSMTSSSSGTSSSSTSTTTTTETSSTDVDGKSKSTFSTITTKSSSGNFADGLAQRQAITGGIDLEKL
VEEQMSKMRSQTFQLMPSSSPNVSPESALVHLDRNSISQYVDSGNKDLLAFNFDMKQYASESINVKTVGNQIEVHASRTTKSSDGSQTKEEFSRSYAMPTNTALDPAKITSGL
FDDGVLSVQLPLSEAIQ
CCCCCCCCCCCCCEEECCCCCCCCCCCCCCCCCCCCCCCCCCCCCCCCCCCCCCCCCCCCCCCCCCCCCCCCCCCCCCCCCCHHHHCCCCCCCCCCCCCCCCCCCCCCHHHC
CHHHHHHHHHHHHHCCCCCCCCCCCCCCCCCCCCCCCCCCCEECCCCCEEEEEEEECCCCCCCEEEEEECCEEEEEEEEEEECCCCCEEEEEEEEEEECCCCCCCCHHHEEEEE
CCCCEEEEEEECCCCCC
98655666642204422676520255444322465323556544443345676544567655542212254444566665554111100146644466652136666893441
536789987664301467777787400001223356642112689749999982699986179999899999999996222268967999999999779998888321589 86
5997189998178889 9

>ID3_C.teleta

MAAEIIPTTTGVVIKMQAPQAPQMSVSASSNLTNGASTTTSSTKMSMSTSTSSSTKTTSTTKITKKTSYSSKTSSSSTITQGGNSTTSSTFKTIKTSSNSGSFSDDIASMFGI
NADQEMEKMKSIMSDQMSRMKSERFSLQSTTSSTSSSGPDSSLVQFDPKSIMDFAENDNLRFNFDMSNFASETVNVKTVGNKIEVHAQRKTKTADGETSEEYSRSYEMPTSQV
ITPEKVTSSLYKEGVLTISLPVSEAMQGVEL
CCCCCCCCCCCEEEECCCCCCCCCCCCCCCCCCCCCCCCCCCCCCCCCCCCCCCCCCCCCCCCCCCCCCCCCCCCCEEECCCCCCCCCCCCECCCCCCCCCCCCCCCCCCCCC
CCHHHHHHHHHHHHHHHHHHHHHHHCCCCCCCCCCCCCCCCCCCCCCCCCCCCEEEECCCEEEEEEECCCCCCCCEEEEEECCEEEEEEEEEEECCCCEEEEEEEEEEECCCCCC
CCHHHEEEEECCCCEEEEEEECCCCCCCCCC
976555678732441368754431124677767774102578765566655566766543211122322334567502321566566665300111257887676430112 46
630212256666420455434330355666555555676543324567642134229859999982899954079999899999999987532796699899999976899 98
9943338885399728999827877776789

>ID10_C.matritensis

CIDKAHNDRLKLNFDVNEFESESINIKTVGNKIEVHAKKKSKKGDEERSEEFSRVYELPTQNAVDAGNVTSSIYKDGVLTIELPVADAIAGTDVSLGQTSSAVKSSSSVSSSS
TSSSTSSKVVGIKSSGLSYLDRISSDFGIDIGQEMDKLKLKMSEEMSNIHQDMFQLMPINSLDADKTIVRLDSDSLKSCIDK
CCEEECCCEEEEEEEECCCCCCEEEEEEECCEEEEEEEEEECCCCCEEEEEEEEEEECCCCCCCCHHHEEEEECCCCEEEEEEECCCCCCCCEEEECCCCCCCCCCCCCCCCC
CCCCCCCCCCCCCCCCCCCCCCCCCCCCCCCCCCEEEECHHHHHHHHHHHHHCCECCCCCCCCEEEEECCHHHHHHHCC
950315981899999189995418999989999999999862258836899999999837998998211688853997189998368888873465112344444444444443
32112455444445676531111246777865554212441313222322232311013326987326763511356653 09

>ID3_Chaetopterus_n.sp.

LIRLDRKSLMDYIDDVDKDRLKFNFDVNEFSSEEVNVNAVNNRIEIHGKRKVTKDGETSTEEYNRAYELPDNVDPETVTSTFFKDGVLTVELPVPAIEEMK
CCCCCCCCCCCCEEEECCCEEEEEEEECCCCCCEEEEEECCEEEEEEEEEEECCCCEEEEEEEEEEECCCCCCHHHEEEEECCCCEEEEEECCCCCCCCC
98656677776413423984999998169995407999999999999998631269758999999997899999111488885399718999817888768 9

>ID8_E.fetida
MSELIPKGQKSSVTSASSSSFSSSSSSSSSSSSSSKVVKQSTSSVKSSSSTSSTSSKSVGIKSKGGSYLDRISSDFGLDTGKEVEKLKLKMSEEMSNIHQDMFQLIAINSPEA
NKT**VVHLDSDSLSSCIDKANKD**__**SLKLNFDVHEFESESINIQTVGNKIEVHAKKKSKKGDEERSEEFSRVYELPTQNAVDAGNVTSSIYKDGVLTIELP**__VADAIAGTNLSLGQA
GSAVQSAGQRTSSAVQSACERTSSTISSSSISKHIGIKNSGLSYLDRISSDFGIDLGMDQLKLKMNEELANVHQDIFQLLPISSPDIEKSVVRLDSESLKSCIDKAHNDRLKL
NFDMNEFESESINIKTVGNKI
CCCCCCCCCCCCCCCCCCCCCCCCCCCCCCCCCCCCCCHHHCCCCCCCCCCCCCCCCCCCCCCCCCCCCCCCCCCCCCCCCCCCCCCCCCCCHHHHHHHHHHHHHHHHHHHHCCCCCCCCCC
CCCCCCCCCCCCCCCCEEECCCCEEEEEEEECCCCCCEEEEEEECCEEEEEEEEEEECCCCEEEEEEEEEEECCCCCCCCHHHEEEEECCCCEEEEEEECCCCCCCCEEEECCC
CCCCCCCCCCCCCCCCCCCCCCCCCCCCCCCCCCCCCCCCCCCCCEEEEEEECCCCCCCCCCCCCCCCCCHHHHHHHHHHHHHCCCCCCHHHHHHHHHCHHHHHHHHHHHHCEEEEE
ECCCCCCCCCCEEEEECCCCC
9876677776655678887667665443334421220111234445677777665434443258874224544444368886426777799989877654442022357764 3
444323344556642122179738999980799953189999899999999998631279637899999999817998999220699963996289999836888777235512 46
6543223333222334553334455663224554334677741442132146777776643211234677754100110258863046766650158899975343121478 7
4055678875247764377 79

>ID5_E.torquata
MLESHMDEEMTKLRKEMCLAPLERSGGE**GQIVKLDNTSILDYLDKGDKN**__**TYKFNFDVHDMKSESVTVKADGNKIEVHAKKTSKKGDGDEQVEEYSRTYELPGGVDPGKVTSSF**__
__**YKDGILTVALP**__VGELTCK
CCHCHHHHHHHHHHHHHHCCCCCCCCCCCCCCCCCCCCCCCCCCEEECCCCEEEEEEECCCCCCCEEEEEECCEEEEEEEEEEECCCCEEEEEEEEEEEECCCCCCCCEEEEEE
CCCCEEEEEEEECCCCCCC
8510899999999987753365456665432111147776431231589749999980799954079999899999999974203798189999999999689999911048985
399718999826899989

>ID3_F.ockeri
MIRQEVMNLLPLERPGSS**QEIVKLDDKSITQYVDDSSGDN**__**RVRFNFDVNEFEAETVCVKAVGNTIEVKAKRKSIKGDEEKSEEYSRTYEMPTKQEIQPATVTSSFYKDGVLTV**__
__**ELP**__ADAPK
CCCCHHHHCCCCCCCCCCCCHHHCCCCCCCCCEEECCCCEEEEEEECCCCCCCEEEEEEECCEEEEEEEEEEECCCCEEEEEEEEEEECCCCCCCCHHHEEEEECCCCEEEE
EECCCCCC
9863033124333767663123204688765202231797399999818999531899998999999999863225973899999999758988999310699845997189 9
97179999

>ID4_H.robusta
MADQQMQQRSSSELLTSEKEQLEREQSKQQINEEQFNRKEQQITQQLITTQQQHEQKLIEQQQQLTQQQQRQEQKQVSTTTSSTMSPSGAKTVKTSKKTVTSKKTTSKTTKST
KSSTSSSSSSSMTSATPERHQEPISTITSTSSSSSSQSATSRQVVIHSSGETYSQHRAHYSRTGEISQQVKSDLDPSQQIFQILLPTTPENAAAT**VIHLRPENLTSAGNDGGK**
EQTAILNFNMSEFESQSIDVRTDGNKVEVHATKKHVNPDGEERKEEFSRIYELPTGDDLDPATLQTSLYQDGVLTIELPIRVDITGPNGNVTGDGSGLSGGSLTITEVK
CCHHHHHHHHHHHHHHHHHHHHHHHHHHHHHHHHHHHHHHHHHHHHHHHHHHHHHHHHHHHHHHHHHHHHHHHHHHHHHHHHHHHHHHHHHHHHHHHHHHHHHHHHHHHHHHHHHH
HHHHHHHHHHHHHHHHHHHHHHHHHHHHHHCCCCCCCHHHHHHCCCCCCCCCHHHHHHHCCCCCCHHHHHHHHCCCCHHHHHHHHCCCCCCCCCCCCCCCCCCCCCCCCCCCCCCC
CCEEEEEEECCCCCCCEEEEEEECCEEEEEEEEEEECCCCCEEEEEEEEEEECCCCCCCCHHHEEEEECCCCEEEEEECCCCCCCCCCCCCCCCCCCCCCCCCCCCCCCCC
9446778877888899999999888999888887788888888998888766668899999999999999999999877777777766777777777788887655565545544
6666655678776653567665432111011233423454310223455311333321023320122334212574134566523567631146521013333344333467
85289999717999973289999799999999988633688268889999999768987898322489846996189996267888887753224667777876323335 9

>ID5_M.pitelkai
MKREMDERVNKVRAKMFHLVPSKDQTS**LQRLGWEDLDNLVGRDN**__**KLKLNFDVDDFNEESMRVECVNNQITVHGRKKAKYHEKLGDIETDEEWTKTYELPAQVDPKSVTSSFFK**__
__**DGVMTVEMP**__APENPEKHTTTLEMQVGDN
CHCHHHHHHHHHHHHHCCCCCCCCCCCCCCCCCCCCEEEEECCCEEEEEEEECCCCCCCEEEEEECCEEEEEEEEECCCCCCCCCEEEEEEEEEEEECCCCCCHHHEEEEEECC
CCCEEEEEECCCCCCCCEEEEEEEECCC
7016999999985121155777665321234666532689709979999980799954079999999999999861100036896799999999999689999933248773 59
9818999817998876505771450799

>ID8_M.pitelkai
MSQIQKSLSQQTSSSKTVSSSKSVTVKKTSMVSKTSSKSVTVKKTGKSFNDAALDFMPKFAITSGIEDDFEVRVKKMREEMLQLMPSTS**TSLLKLDAPSLKELVGKDG**__**KAHLN**__
__**FDVSQFNSETVHVKTEGNKMEVHAKKVSRDGDDEQTQEFSRTYELPEGVKSDMVESSFFKDGVLTVALP**__LAAIEAAK
CCCHHHHHHCCCCCCCCCCCCCCCCCCCCCCCCCCCCCCEECCCCCCCCCCCCCCCCCCCCCCCCCCHHHHHHHHHHHHHHHHHHCCCCCCCCCCCCCCCCCCCCEEECCCEEEEE
EEECCCCCCCEEEEEECCEEEEEEEEEEECCCCEEEEEEEEEEECCCCCCCCCEEEEECCCCEEEEEEECCCCCCCC
8511222211466510124454322342212356777411125665567777766788878887345665314678876765116787642001256654202305984999 9
9807999762799998999999999865238975999999888589999900048985499708999806889889 9

>ID15_N.dendritica
MSNPETAVVQKKVSSSSQVSSASSYTSSSTVQKTTYSTKTVSQAVKSSKNSSITVRSNTDTSFLDTMGKRLGIDFDKHLMDLESKADDQMQKLKEEFHIMNLQKSGSG**SEVVK**
IDDNSLSQYLDEGKN__**ALKLNLDVHEFKSETVSVKCSGNKIEVHAQKTAKKGEDESNEEYSRTYELPNRVDADKVTSQFFKDGVLTVELP**__MAAIEGAKEVKE
CCCCCCCCCCCCCCCCCCCCCCCCCCCCCCCCCCCCCCCCCCHHHHHHCCCCCCCCCCCCCCCCCCCCCCCCCCCCCCCCCCCCCCCCCCHHHHHHHHHHHHHHHCCCCCCCCCCCCHHHC
CCCCCCCCEEECCCCEEEEEEECCCCCCCEEEEEECCEEEEEEEEEEECCCCEEEEEEEEEEECCCCCCCCEEEEECCCCEEEEEEECCCCCCCCCCCC
9986310012334577655678765555556666641223221135566653335677764201245667766555544300168888678875531355577888730111
34676532134279849999980699944079999899999999986430688178999988858999990004899649971899981788864467899

>ID1_P.gouldii
MSMQITKKMTTKTTVTKTSTTGGGPPQTESHSTRKVSVQSSGGSFLDQFGSAGTTKDLALTQAGSSPDIDAEIEKIKKEFNMRTLMRSDSHE**GNIKLDTKDLVSFMDKDKN**__**KL**__
__**NFNFDVSDFKTETLNVKTVGNKIEVHGEKISKTGGEERKEEYSRSYDLPSSVDAQSCKSSMFKDGVLTVELP**__VTAALKESEDL
CCCCCCCCCCCCCCCEECCCCCCCCCCCCCCCCCCCCCCCCCCCCCCCCCCCCCCCCCCCCCCCCCCCCCHHHHHHHHHHHHHHCCCCCCCCCCCCCCCCCCCCCCCCCEEEEECCCEE
EEEEECCCCCCCEEEEEEECCEEEEEEEEEECCCCCEEEEEEEEEEECCCCCCCCEEEEEECCCCEEEEEEECCCCCCCCCCC
9866665666655310001247888654456443434446887544567889983101246667641456688876432068755688877653212346752035654985 9
999980799965189999899999999998514771589889989968999991104898549971899982798886435 89

>ID1_Polygordius_n.sp
MSSQVVKKVTSSSVTTSQKTSSSYQVQSTSKSSLSSTSASQTVIRSDGGAIDMDAELGKMSLKMDEQMSRMKTEMLKLMPLGPSGAANELVKLDTKTVQEFADDGKKALKFDF
DVNEFKSESVVVKAEGNQVHVHAKKIVRTGDDEQVEEYSKSYELPAGVEPGKVTSSLSKPGVLTVELPCAGLTM
CCCCCCCCCHHHCCCCCCCCCCCCCCCCCCCCCCCCCCCCCCCCCCCCCCCCCCHHHHHHHHHHHHHHHHHHHHHHHHHHCCCCCCCCCCCCCCCCCCCCCEECCCCCCEEEEEE
ECCCCCCCCEEEEEECCEEEEEEEEEEEECCCEEEEEEEEEEECCCCCCCCEEEEEECCCCEEEEEEECCCCCC
987432210110244455666520122356667766766543102688876402113467889999999997555532135778764310102466642010268944999999
818999540899998999999999765417965999999999977999991216999649972899981686789

>ID5_S.pavonina
MSSDKTVSQTSQTNQSSSYSSSSSSSFSSSKTVKTSSTVKSMSVKSSSSSNAISIRSAGSSFSDTRSKELGYDVDTKMKELTAKMDNEMDTVRREFFRMTSLESLESADKVTK
IDSSSLSKYMDDTNKDKLKFNFDVDEFESESISVKAVGNQVEVHAKKKSRKDGQESSEEFTKAYELPTSRDIDPAKVTSSFYKDGVLTVELPLEAAIMEKKE
CCCCCCCCCCCCCCCCCCCCCCCCCCCCCCCCCCCCHHHCCCEEECCCCCCCEEEEECCCCCECCCCHHHHHHCCHHHHHHHHHHHHHHHHHHHHHCCCCCCCCCCCCCCCCC
CCCCCCCCCCCCCCCCEEEEEEECCCCCCCEEEEEECCEEEEEEEEEEECCCCEEEEEEEEEEECCCCCCCCHHHEEEEECCCCEEEEEEECCCCCCCCCC
998754467776678887778887776545554561111000010466776413232575301037413442011222133336777767766553045566555665323 34
567775532112589749999980899953079999899999999975313796699999999997699878992115999649962899980788877668 9

>ID1_S.nudus
MAMQITKKVTTKTTVKTTSTVGGVPVTTGSSSRTVNVSSSKRSFLDDFGSGLGIDLTKSMDEEIERMKREMFNMKALEPAGSSNQVVKLDSNSLMKFMDKDHQDKLRFNFDVH
EFSSESVNVKTVGNTIEVHGTKTVKKGGEEQSESYSRSYELPCPNVDPNKVTSSIFKDGVLTVELPVSEVEK
CCCCCCCCCCCCCCCCCCCCCEEECCCCCCCCCCCCCCCCCCCCCCCCCCCCCCHHHHHHHHHHHHHHHHHCCCCCCCCCCCCCCCCCCCCCCCCCCEEECCCCEEEEEEEEC
CCCCCCEEEEEECCEEEEEEEEEEECCCCEEEEEEEEEEECCCCCCCCCEEEEEECCCCEEEEEECCCCCCC
972345444566544566641121135788521368888865434788899983233366888788742211576456766653211245566653112226983999998 06
999651899998999999999863127864899999999768999991126998639971899981898889

>ID1_Spinther_n.sp.
MSGVTRKVTKTTVTSYSESYSSSSSSHTVRQDGQVVEHQEQHSSSHSSSGPQVQQVQIRTSSDSFLDAETRRKLTTDLDLGCLEPASKNTNTSSVTMEINTNGVAKDTNKSSV
TKETNTSSVTKEIKTSGVTKEIKTSGVTKATDVKQLAEDEMQRLRSEMKLMTLRSEEDSAVRSGSNDPNVVRITREALSEFTDDKDPQRLKFNLDVHEMASESLQVKAIGNKI
EVHGKRYRKSGGNASTAGEESEEFSRTYELPGNLEKEKVTSSFYKDGVLTVELPRDAVKSTA
CCCCCCCCCCCCCCCCCCCCCCCCCCCHHHHHHHHHHHHHHHHHHCCCCCCCCCCCEEECCHHHHCCCCCCEEEEEEEECCCCCCCCCCCCCCCEEEEECCCCEECCCCCCE
ECCCCCCCCCCCCCCCCCCCCCCCCCCCCCCCCCCCCCCCCHHHHCCCCCCCCCCHHHHHHCCCCCCCCCCCCCCCCCCCCEEECCCCCEEEEEEECCCCCCCCEEEEEECCEE
EEEEEEEECCCCCCCCEEEEEEEEEEEEECCCCCCHCHEEEEEECCCEEEEEEECCCCCCCC
99765432211101244445677764034567899999998754203568732120110001221255651575455432455666667777752378831331231686411
011244445321245675323455687554345402121011211212011345135655236788852112335677541424789759999981899943179999899999
9999841012677763058988888688479999900048996089649999826888999 9

Cluster B1 – Multiple sequence alignments

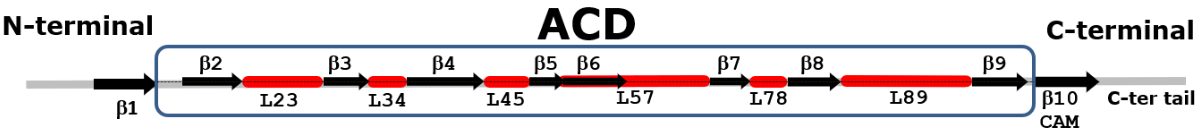

PROMALS3D alignment

PROMALS3D multiple sequence and structure alignment server http://prodata.swmed.edu/promals3d/promals3d.php (Pei et al. 2008)

```
Conservation:                                           76777  5 575   7    79 56767 7              6  77567 9679      5        75 56697777 79
ID1_Spinther_n.sp.         1   -VVRIT-REALSEFTDDKDPQRLKFNLDVHEMASESLQVKAIGNKIEVHGKRYRKSGGNASTAGEESEEFSRTYELPG-N--LEKEKVTSSFYKDGVLTVELP   98
ID1_P.gouldii              1   GNIKL--DTKDLVSFMDKDKNKLNFNFDVSDFKTETLNVKTVGNKIEVHGEKISKTG-----GEERKEEYSRSYDLPS-S--VDAQSCKSSMFKDGVLTVELP   93
ID3_C.teleta               1   SLVQF--DPKSIMDFAEND--NLRFNFDMSNFASETVNVKTVGNKIEVHAQRKTKTA-----DGETSEEYSRSYEMPT-SQVITPEKVTSSLYKEGVLTISLP   93
ID10_A.lacteospumosus      1   -VVLLD-SDSLKSCIDKAHGDRLKLNFDVNEFESESINIKTVGNKIEVHAKKKSVKG-----DEERSEEFSRVYELPT-QNAVDAGNVTSSIYKDGVLTIELP   95
ID11_A.lacteospumosus      1   -VVRLD-SEALKSCIDKAHGDRLKLHFDVNEFESESINIKTVGNKIEVHAKKKSVKG-----DEERSEEFSRVYELPT-QNAVDAGNVTSSIYKDGVLTIELP   95
ID8_E.fetida               1   -VVHLD-SDSLSSCIDKANKDSLKLNFDVHEFESESINIQTVGNKIEVHAKKKSKKG-----DEERSEEFSRVYELPT-QNAVDAGNVTSSIYKDGVLTIELP   95
ID10_C.matritensis         1   -------------CIDKAHNDRLKLNFDVNEFESESINIKTVGNKIEVHAKKKSKKG-----DEERSEEFSRVYELPT-QNAVDAGNVTSSIYKDGVLTIELP   84
ID6_A.lacteospumosus       1   -LVRLD-SDSLRSCIDKAHGDRLKLNFDVNEFESESINIKTVGNKIEVHAKKKSKKG-----DEERSEEFSRVYELPT-QNAVDSGNVSSSIYKDGVLTIELP   95
ID3_F.ockeri               1   -IVKLDDKSITQYVDDSSGDNRVRFNFDVNEFEAETVCVKAVGNTIEVKAKRKSIKG-----DEEKSEEYSRTYEMPTKQE-IQPATVTSSFYKDGVLTVELP   96
ID5_S.pavonina             1   -VTKIDSSSLSKYMDDTNKD-KLKFNFDVDEFESESISVKAVGNQVEVHAKKKSRKD-----GQESSEEFTKAYELPTSRD-IDPAKVTSSFYKDGVLTVELP   95
ID3_Chaetopterus_n.sp.     1   -LIRLD-RKSLMDYIDDVDKDRLKFNFDVNEFSSEEVNVNAVNNRIEIHGKRKVTKD-----GETSTEEYNRAYELPD-N--VDPETVTSTFFKDGVLTVELP   93
ID5_M.pitelkai             1   -LQR---LGWEDLDNLVGRDNKLKLNFDVDDFNEESMRVECVNNQITVHGRKKAKYHEK-LGDIETDEEWTKTYELPA-Q--VDPKSVTSSFFKDGVMTVEMP   95
ID15_N.dendritica          1   -VVKI--DDNSLSQYLDEGKNALKLNLDVHEFKSETVSVKCSGNKIEVHAQKTAKKG-----EDESNEEYSRTYELPN-R--VDADKVTSQFFKDGVLTVELP   92
ID1_S.nudus                1   -VVKLD-SNSLMKFMDKDHQDKLRFNFDVHEFSSESVNVKTVGNTIEVHGTKTVKKG-----GEEQSESYSRSYELPCPN--VDPNKVTSSIFKDGVLTVELP   94
ID4_B.viridis              1   -LVHLD-RNSISQYVDSGNKDLLAFNFDMKQYASESINVKTVGNQIEVHASRTTKSSD----GSQTKEEFSRSYAMPT-NTALDPAKITSGLFDDGVLSVQLP   96
ID1_Polygordius_n.sp       1   -LVKL--DTKTVQEFADDGKKALKFDFDVNEFKSESVVVKAEGNQVHVHAKKIVRTG-----DDEQVEEYSKSYELPA-G--VEPGKVTSSLSKPGVLTVELP   92
ID5_E.torquata             1   -IVKLD-NTSILDYLDKGDKNTYKFNFDVHDMKSESVTVKADGNKIEVHAKKTSKKGD----GDEQVEEYSRTYELPG-G--VDPGKVTSSFYKDGILTVALP   94
ID10_A.marina              1   -IIKLD-NTSILDYIDKDDRDLLKFNFDVSGYQSQTVIVKSDGNKIEVFAKKTSKVGD-----EERSEEFSRSYEMPRDGA-IDASKITSSFYKDGILSVE--   93
ID8_M.pitelkai             1   -TSLLK-LDAPSLKELVGKDGKAHLNFDVSQFNSETVHVKTEGNKMEVHAKKVSRDG-----DDEQTQEFSRTYELPE-G--VKSDMVESSFFKDGVLTVALP   93
ID4_H.robusta_             1   -IHLRP-ENLTSAGNDGGKEQTAILNFNMSEFESQSIDVRTDGNKVEVHATKKHVNPD----GEERKEEFSRIYELPT-GDDLDPATLQTSLYQDGVLTIELP   96
Consensus_aa:                  .lhpl..ps........sppp.lphNFDhpp@pSEolpV+hsGNplEVHtp+.spps.....spEppEE@SRsYEhPs....l-s.ploSSh@KDGVLTlpLP
Consensus_ss:                               eeeee   eeeeeeee    hhheeeeee  eeeeeeee            eeeeeeeeeeee        hhheeeeee   eeeee
```

MEGA 7 alignment (Kumar et al. 2016)

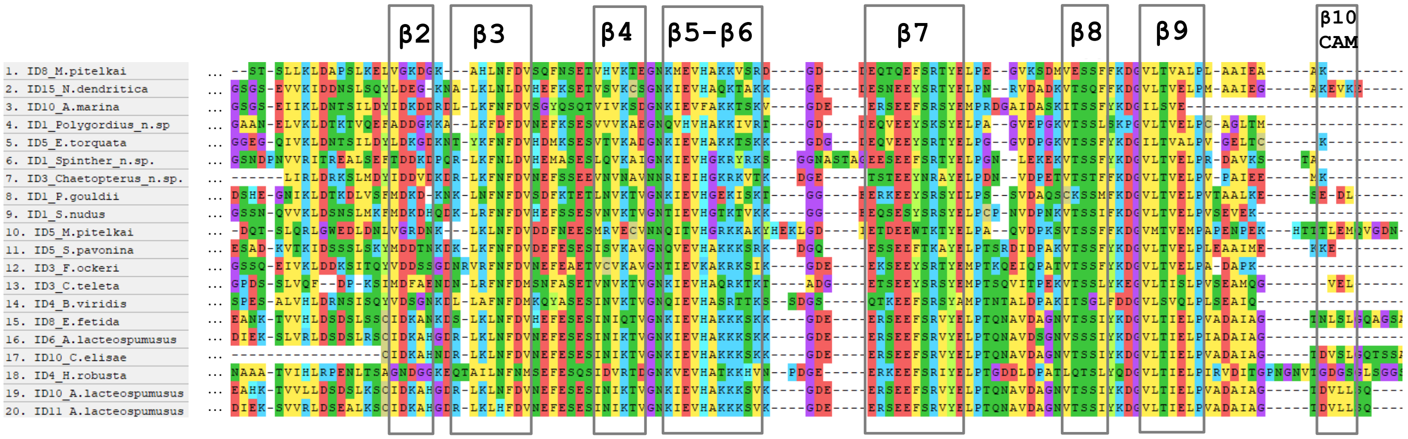

Cluster B2 – Phylogenetic trees

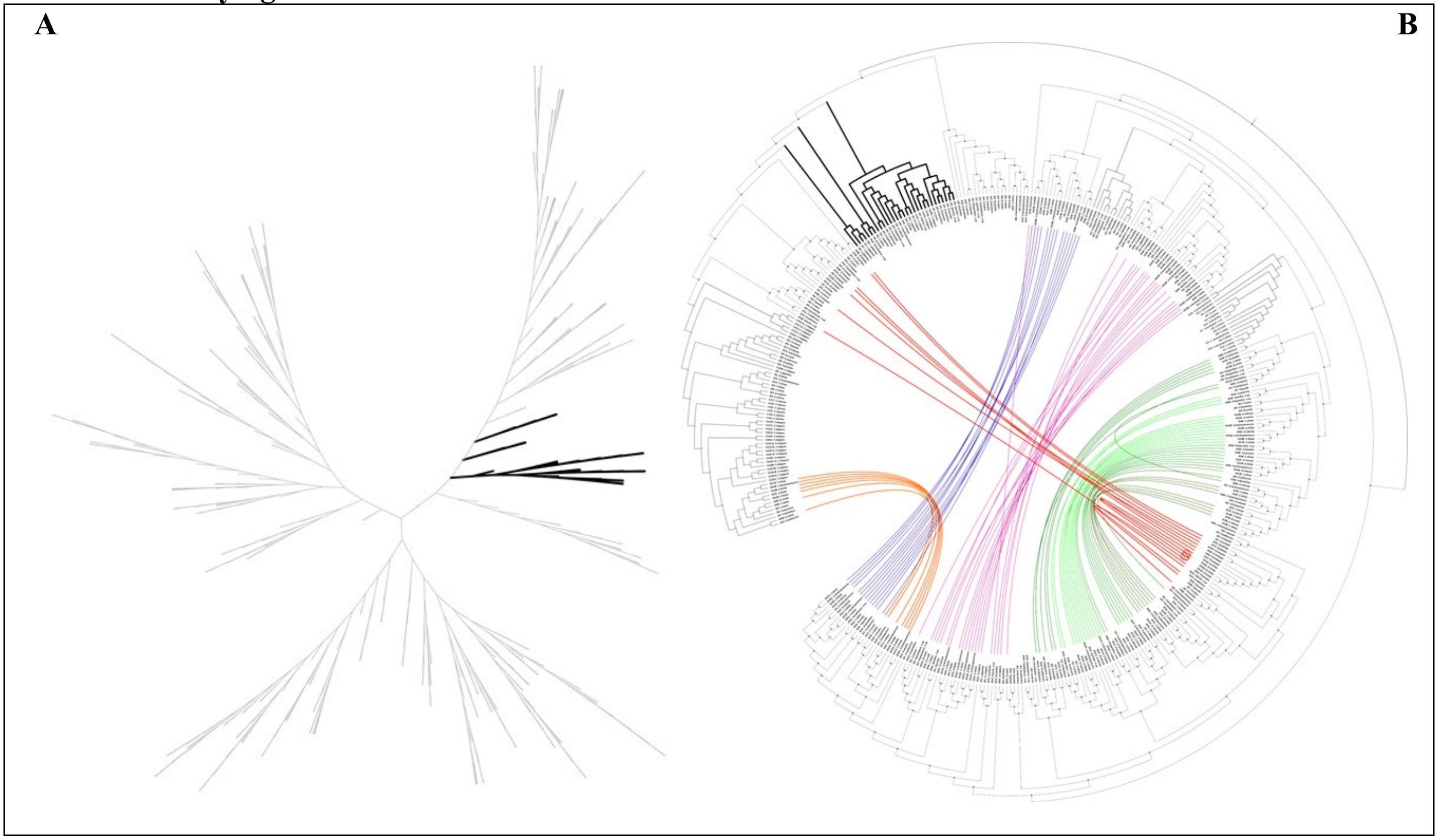

Supplementary Figure 11. A, unrooted ML phylogenetic tree, branch lengths not displayed. Small Hsp from cluster B2 in black. **B**, rerooted ML phylogenetic tree, small Hsp from cluster B2 in black and colored connection lines for the two corresponding ACDs of dimeric sHsps (not covered in this work).

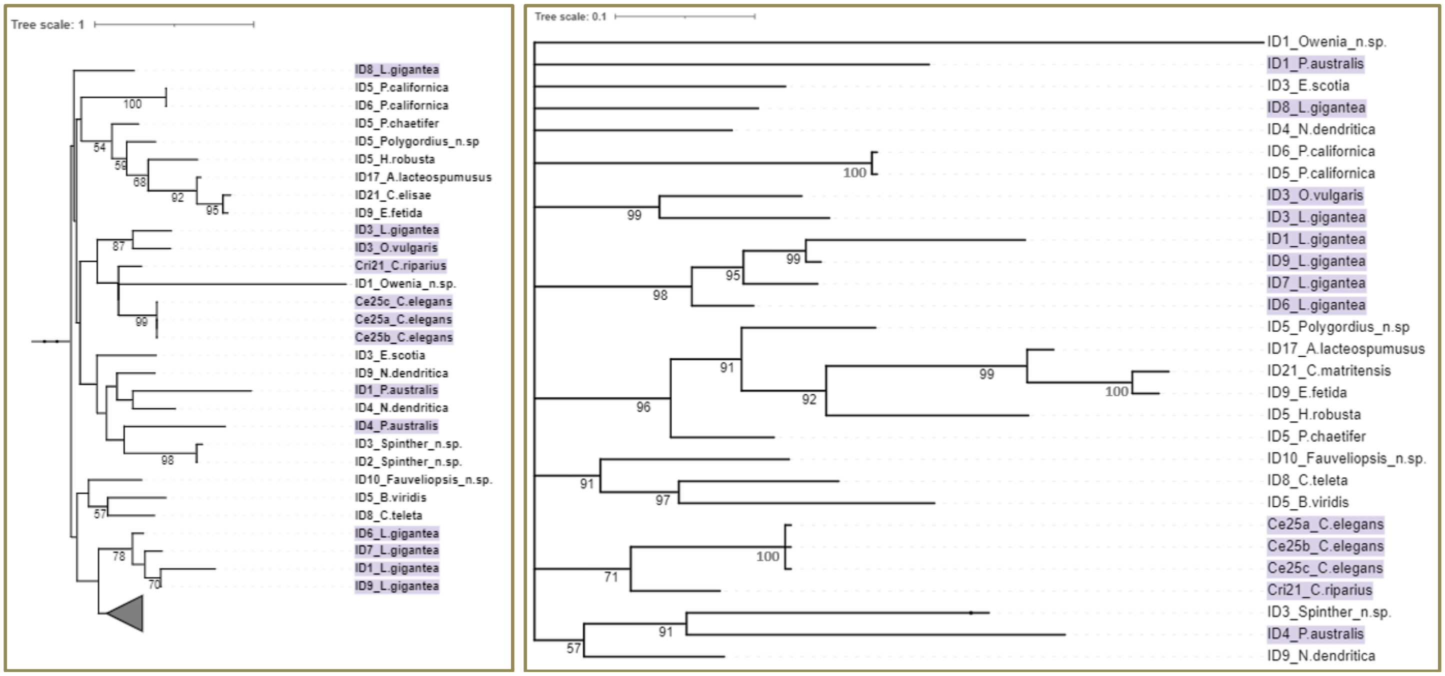

Supplementary Figure 12. Left, clade recovered by ML analysis displaying branch lengths and bootstrap >50. **Right**, clade recovered by Bayesian Analysis displaying branch lengths and posterior probabilities in percentage.

Cluster B2 – Predicted Secondary Structures

Tool: PSSPRED online service (Yan et al. 2013) https://zhanglab.ccmb.med.umich.edu/PSSpred/

(**Bold black**: ACD domain delimited from the β2-strand to the β9-strand (Poulain et al. 2010); **underlined**: β3-β9 region, used in multiple sequence alignment for phylogenetic study; **H**:helix, **E**:strand, C:coil)

>ID17_A.lacteospumosus
MALVMRDFPVMRYEFDFFERIKQQMNLEFKQIEEEIDKMRKQMLHLLPMDP**IDVTVQEIKPAVPIVEEKGETKLKLEFNVKEFRPEDLEVKLLGNNILQVTAHREDKTESGMS**
KICYLRKYSLPEGIDKDHIKPTLTKDGVLTIEAPAPALKPKERMIPIEYKVQ
CCCCCCCCCCCCCCCCCCHHHHHHHHHHHHHHHHHHHHHHHHHHCCCCCCCCCCCCCCCCCCCCCEEECCCCEEEEEEECCCCCCEEEEEEECCEEEEEEEEEEECCCCCEE
EEEEEEEEECCCCCCCCEEEEEECCCCEEEEEEECCCCCCCCEEEEEEEEEC
99866788888888774214788877676256667667652442346777544431013566673353279759999980799954079999899999999775521588268
9888999848999991105899649970899981789888871673799719

>Ce25a[*Caenorhabditis elegans*]
MPTYTRTYSYSKREMSERRIDVNRSNYSVIDNEFGNMRDRFEQEMRRVEEEMKRLRSEFEGYRPNGGPPAAISNQPYNAYSNTSSHHETSNRTGGFGSPLPPPSFHGPSDLMA
HRPTY**DPYLDNLKSPLIKDESDGKTLRLRFDVANYKPEEVTVKTIDNRLLVHAKHEEKTPQRTVFREYNQEFLLPRGTNPEQISSTLSTDGVLTVEAP**LPQLAIQQ
CCCCCCHHHHHHHHHHHHCCCCCCCCCCCCCCCHHHHHHHHHHHHHHHHHHHHHHHHHHCCCCCCCCCCCCCCCCCCCCCCCCCHHHHHHCCCCCCCCCCCCCCCCCCCCCCC
CCCCCCHHHCCCCCCCEEEEECCCEEEEEEECCCCCCCEEEEEECCEEEEEEEEEEECCCCEEEEEEEEEEECCCCCCCCCEEEEECCCCEEEEEEECCCCCCCC
99742102477643232123456778622355402444332455566788777641454214555666555666553332211110122211255445678620157510013
4654211211245763476644985999998079995408999989999999999974137965999999896899999330489963997189998079877689

>Ce25b[*Caenorhabditis elegans*]
MSERRIDVNRSNYSVIDNEFGNMRDRFEQEMRRVEEEMKRLRSEFEGYRPNGGPPAAISNQPYNAYSNTSSHHETSNRTGGFGSPLPPPSFHGPSDLMAHRPTY**DPYLDNLKS**
PLIKDESDGKTLRLRFDVANYKPEEVTVKTIDNRLLVHAKHEEKTPQRTVFREYNQEFLLPRGTNPEQISSTLSTDGVLTVEAPLPQLAIQQ
CCCCCCCCCCCCCCCCCCCCHHHHHHHHHHHHHHHHHHHHHHHHHHCCCCCCCCCCCCCCCCCCCCCCCCCHHHHHCCCCCCCCCCCCCCCCCCCCCCCCCHHHCCCCC
CCEEEEECCCEEEEEEEECCCCCCEEEEEEECCEEEEEEEEEEECCCCEEEEEEEEEEECCCCCCCCCEEEEECCCCEEEEEEECCCCCCCC
9876434345777787540145667776777667888887655323221246656666554433101001012210234545578861025642111345431012113467
6347665498599999807999530899998999999999996303686589999999996899999220489964997189998088655689

>Ce25c[*Caenorhabditis elegans*]
MAHRPTY**DPYLDNLKSPLIKDESDGKTLRLRFDVANYKPEEVTVKTIDNRLLVHAKHEEKTPQRTVFREYNQEFLLPRGTNPEQISSTLSTDGVLTVEAP**LPQLAIQQ
CCCCCCCCHHHCCCCCCCEEEEECCCEEEEEEEECCCCCCEEEEEEECCEEEEEEEEEEECCCCEEEEEEEEEEECCCCCCCCCEEEEECCCCEEEEEEECCCCCCCC
999876441330267874367753985999998079994307999989999999997531268659999999996899999120589854997189998179876689

>ID21_C.matritensis
MALVMRDFPVLRHDFNFFERIKQQMNMEFKQMEEEIEKMRKQMLHLLPM**DPVDVMVQECKPSVPVVEEKGETKLKLEFNVKEFRPEDLEVKLLGNNILQVTAHREDRTDSGMS**
KMRYLRKYTLPEGIDKEHIKPTLTKDGVLTIEAPAPALKPKERMIPIEYKHQ
CCCCCCCCCCCCCCCCCCCHHHHHHHHHHHHHHHHHHHHHHHHHCCCCCCCCCCCCCCCCCCCCCCCEEECCCCEEEEEEECCCCCCCEEEEEEECCEEEEEEEEEEEECCCCEE
EEEEEEEEECCCCCCCCEEEEEECCCCCEEEEEECCCCCCCCEEEEEEEECC
998645786545776656135547775776788776666534215677775421000236766623524896599999806999531799999999999998787641489659
9899999848999991105898549970899981788888870674798239

>Cri21 [*Chironomus riparius*]
MAENKRNIPIQFNRDFSVIDSEFGQIRERFDQEMRRMEDEMNKFRSELMNRESNFFETTSSSTTSNNSLSRA**QSNVSDINSPLIQDDGDNKVLKLRFDVSQYAPEEIVVKTVD**
NKLMVHAKHEEKTDTKSVYREYNREFLLPKNVNPESIRSSLSKDGVLTVDAPLPAIAGPGEKMIPIAHN
CCCCCCCCCCCCCCCCCCCCCCCCHHHHHCCCCCCHHHHHHHHHHHHCCCCCCCCCCCCCCCCCCCCCCCCCCCCCCCCCCCCCCEEEECCCCEEEEEEEECCCCCCCEEEEEEC
CEEEEEEEEEEECCCCEEEEEEEEEEECCCCCCCCEEEEEECCCCEEEEEEECCCCCCCCCEEEEEECC
99877767755689830141001202322126420124455432100013211113454456556543344333321246763022026966999998079995417999989
999999999741268658998999995899999220589964997189998178887778616601319

>ID9_E.fetida
MALVMRNFPVMRYDCDFFERIKKQMNMEFKQIEEEIEKMRHQMLHLVPV**DPVDVMVQEIKPSVPIVEEKGETKLKLEFNVKEFRPEDLEVKLLGNNILQVTAHREDKNEHGMS**
KICYLRKYTLPEGIDKEHIKPTLTKDGVLTIEAPAPALKPKERMIPIEYKVQ
CCCCCCCCCCCCCCCCCCCHHHHHHHHHHHHHHHHHHHHHHHHHHCCCCCCCCCCCCCCCCCCCCCCCEEECCCCEEEEEEEECCCCCCEEEEEEECCEEEEEEEEEEEECCCCEE
EEEEEEEEECCCCCCCCCEEEEECCCCEEEEEEECCCCCCCCCEEEEEEEEC
99865567666677553422443312034467777666543553357888543210013577664133169759999980799954079999899999999656641489179
9989999848999991104899549971899981798887870573799609

>ID3_E.scotia
MALTPFERIFQISRPNHNDSFGMVDFRSRMDREMRDIEMQMNEASSRMQRLISLPDFGQQQSRRGWGDIQMPA**VTDNWRVDSPTFVEENGQRKMKLNFDMGHFTPEEITVKTK**
DNNLIVQAKHEEKSDSGHVFREYNKMYQLPEGVKLDEMKSFLTQNGSLCVEAPVPLPVLLPEANEKTIAIQHQ
CCCCCCCCCCCCCCCCCCCCCCCCCCCCCHHHHHHHHHHHHHHHHHHHHHCCCCCCCCCCCCCCCCCCCCCCCCCCCCCCCCCCEEEEECCCEEEEEEECCCCCCCCEEEEEE
CCEEEEEEEEEEECCCCEEEEEEEEEEECCCCCCCCEEEEEECCCCEEEEEEECCCCCCCCCCCCEEEEEEEC
98777775543356777787776665321113567888776454556553124776443222354222222211123445676314541698499999807999541799998
99999999975403698699999999995899999220599963997089998078754456788637612419

>ID5_H.robusta
MNLPLERMQSIERQMVQLRNLMDKELSQMRKQMYQVLHYEPASEAQALAELNPAAPIVEENGETKLKLDFNVKGFKPEEVQVKIVGDNVLQVRAETGTKTDSGVSHRYYVRHY
ALPDGIKADQIKPTMSKEGVLSIEAPAPHLKPTERNIPIKYIEGK
CCCCCCCCCCHHHHHHHHHHHHHHHHHHHHHHHCCCCCCCCCCCCCCCCCCCCCCCCCEEECCCCEEEEEEEECCCCCCEEEEEEECCCEEEEEEEEEEECCCCEEEEEEEEEE
ECCCCCCHHHEEEEECCCCCEEEEEECCCCCCCCEEEEEEEECCC
9998876531011234477799999988764310323468765542011026766513531697599999817999540799997596899998999750697699999999
58999990014898549970899981788988860773699428 9

>ID4_N.dendritica
QLQFNMRCFKPEELTVKTVDNKLQVYAKHEEETDHGVIFQEYRREFQLPEGMDLEQMKSKLSPDGILTVEGPLPVPALEEAPKEHTIPIQHQTSEEKKAVTK
CEEEECCCCCCCEEEEEEECCEEEEEEEEEECCCCCEEEEEEEEEEECCCCCCCCEEEEEECCCCCEEEEEECCCCCCCCCCCCEEEEEEECCCCCCCCCC
87997289996308999989999999999853147866999999788689999010589853997089998268876556787527877735874323235 9

>ID9_N.dendritica
MSLWPMDMSERRVPILRSRSLLNPSWDVSHWDDFQDFGQFLRSMDRQFKMMDQQLNDMFSHFNRLAPLDSTGPRLERLAITDKDTVACAPQFRSELSMVPAHSPGAAVGSPSQ
MVEGFGFRNPVITKPDGSKHLQLEFDVRHFKPEELNIKTLDNKLVVHAKHQEESDDSQVYREYQRQFLLPEGIELDKMKSVLSPEGVLTVEAPLPSSAVEPPKEHVIPIEHQS
SRTESITE
CCCCCCCCCCCCCCCCCCCCCCCCCCCCCCCCCHHHHHHHHHHHHHHCCCCHHHHHHHCCCCCCCCCCCCCCCCCCCCCCCCCCCCCCCCCCCCCCCCCCCCCCCCCCCCCCCC
CCCCCCCCCCCEEEEECCCEEEEEEEECCCCCCCEEEEEECCEEEEEEEEEEECCCCEEEEEEEEEEECCCCCCCCCEEEEECCCCCEEEEEECCCCCCCCCCEEEEEEECC
CCCCCCCC
9987765444555633344333554345566761221223445554424541112343212323465435687655544444431123520033211111356311245542 1
1233467776346764598499999817999540799998999999999764226975999999999957999991104898459971899981798765567751787886 26
75544579

>ID1_L.gigantea[*Lottia gigantea*]
DEHIRQLRHPLVTRPDGTEALDLKIDLSHYKPEEIHIRTVGHELSVHAKHEDKTDHSSVYQEYSRKFSLPAHVDPEHVQSSLSRDGILRI
CCCCCCCCCCCEEEEECCCEEEEEEECCCCCCCCEEEEEECCEEEEEEEEEEECCCCEEEEEEEEEEECCCCCCCCEEEEEECCCCCEEC
963112578744677469749999980799954079999899999999987403786699999999996899999110489854899422 9

>ID3_L.gigantea[*Lottia gigantea*]
PFVTDLDGNKKLSIRFDVSQFKPEEISVRTMDNKLMIQAKHTEESPGRKVYREFSKQYILPNKIDPVKLESILSQDGVLSIQAPAP
CCCEEEECCCEEEEEEECCCCCCCEEEEEEECCEEEEEEEEEECCCCCEEEEEEEEEEECCCCCCCCEEEEEECCCCCEEEEEECC
984046369739999980799953089999899999999985214674699999999996899999120599963997089996189

>ID6_L.gigantea[*Lottia gigantea*]
MKKLRDPIVKKDDGSEALELQLDVSNFKPEEITVTTSGKQLSVHAKHEEKTGNSSVYQEFNRSFTLPDGVKPDTVQSTLSKDGILSITGPVDRALEGPKKIPIEHKK
CCCCCCCCEEEEECCCEEEEEEEECCCCCCCEEEEEECCEEEEEEEEEEECCCCEEEEEEEEEEECCCCCCCCEEEEEECCCCCEEEEEECCCCCCCCCEEEEEECC
98423774488846985999998169995407999989999999999863069669999999999679999910058985599708999827988888706756863 9

>ID7_L.gigantea[*Lottia gigantea*]
TQMKRLRHPIVKRADGSEALELQLDLSNFKPEEITVKTSGKELAVHAKHEEKSENSSVYQEYCRKFVLPDNVNPELVESTFSKDGILTITGPAALEA
CCCCCCCCCCEEEEECCCEEEEEEECCCCCCCCEEEEEECCEEEEEEEEEEECCCCEEEEEEEEEEECCCCCCCCEEEEEECCCCEEEEEEECCCCC
9853247862688855984999998079995407999989999999999751279759999999999589999000589863997089998079999

>ID8_L.gigantea[*Lottia gigantea*]
ITQDHEGRKFRLQFDVRQFKPEEIMVKTEGQKLEIHAKHEEKGDNKSVYSEYHRQYLLPNELKTDRLVSKLSRDGVLSIEAPLP
CEECCCCCEEEEEEECCCCCCCEEEEEEECCEEEEEEEEEEECCCCEEEEEEEEEEECCCCCCCCEEEEEECCCCEEEEEEECC
812158965999998079995318999989999999999864215975899999999968999991204898549971899972 89

>ID9_L.gigantea[*Lottia gigantea*]
MNRLRCPIVTKPDGSKALELQLDLSNYKPEEINIKTAGKELSVHAKHEEKTENSSVYQEFTRKFTLPEGVNPEKVESTLSKDGILTISGPADNALEGPKAIPIEHK
CCCCCCCCEEEEECCCEEEEEEEECCCCCCCEEEEEECCEEEEEEEEEEEECCCEEEEEEEEEEECCCCCCHHHEEEEECCCCCEEEEEECCCCCCCCCEEEEEEC
9865576258885498599999816999541799998999999999998642497699999999995899999111488854997089998179888887056236 29

>ID3_O.vulgaris[*Octopus vulgaris*]
MSRDLMIPLSTMPWGFFDRQRSLFSEIHKNMEDEFKRFDLELDRIRKDLFKLEPLSIEDFGKTNMLQVENPIVKDSEGNKKLALRFDVSNFKPDEICVKTTDNTLHIQAKHEE
KTPQKQVYREFSRHYTLPKSVDPLKLSSILTKDGVLHIEAPAPEAIEAPSERLIPIQKC
CCCCCCCCCCCCCCCCCCCHHHHHHHHCHHHHHHHHHHCHHHHHHHHHHCCCCCCCCCCCCCCCCCCCCCCEEEEECCCEEEEEEEECCCCCCEEEEEECCEEEEEEEEEE
ECCCCEEEEEEEEEEECCCCCCCCEEEEEECCCCCEEEEEECCCCCCCCCEEEEEEEC
9864543334688765544312112211146668877400334566565324788765432220024687346686298699999816999540799998999999999853
12686599999999958999991005898549970899981788777788627703529

>ID1_Owenia
YVVRQTTTETSRQKVLRLKSAEATPAIVGMQHKDVDQDGIKLFQMHFDVSKYSFDDMELKAVDNTLVVIHKKNERVGARKYKIREQSKQFVLPNGVDIG
CCCCCCCCCCCCCCCCCCCCCCCCCCCCCCCCCCCCCCCCEEEEEEEECCCCCCCEEEEEEECCEEEEEEEEEEEECCCCEEEEEEEEEEECCCCCCCC
91244541221344323466555642135655443236870699999716999732899998998999853566641881689889878867999989 9

>ID5_P.californica
MQMMPMGNPPLAIQGASAFPAIEPMPISHPIIDGGPGNEKMELRFDVREFHPSEISVTTQGNMLAVRARHQEQSESGTHKSSSWKEYNRQYLLPTQVDPAKIKPNLSPEGILT
IESRLTPAIEGKKKVRLAVDAGM
CCCCCCCCCCCCCCCCCCCCCCCCCCCCCCCEEEEECCCEEEEEEEECCCCCCCEEEEEECCEEEEEEEEEEEECCCCEEEEEEEEEEEEEECCCCCCHHHEEEEECCCCCEE
EEEECCCCCCCCCCEEEEEECCC
998888788754444334664211124587435565498599999817999541799998999999999985430378178886227999996899999111488844997189
99836887677750566420489

>ID6_P.californica
MPISHPIIDGGPGNEKMELRFDVREFHPSEISVTTQGNMLAVRARHQEQSESGTHKSSSWKEYNRQYLLPTQVDPAKIKPNLSPEGILTIESRLTPAIEGKKKVRLAVDAGM
CCCCCCCEEEEECCCEEEEEEEECCCCCCCEEEEEECCEEEEEEEEEEECCCCCEEEEEEEEEEEEEECCCCCCHHHEEEEECCCCCEEEEEECCCCCCCCCEEEEEECCCC
998887302231698499999807999540799998999999999985422588268887448999997899999112488734997089999837888777750566411589

>ID5_Polygordius_n.sp
MRQQMLQLVPFDETAIDRNKGMIEVKRPIVESEIPGQGKLKLEFDVKQFKPEEVEVKVMGNNMLQINALHEEKSDTGYSRRQYCRQYLLPEGVHADKIQPSLTQDGVLTIEAP
APAPGPKEKKIPIEYKK
CCCCHHHCCCCCCCCCCCCCCHHHHCCCCCCEEECCCCEEEEEEEECCCCCCEEEEEEECCCEEEEEEEEEECCCCCEEEEEEEEEEECCCCCCCCCEEEEECCCCCEEEEEE
CCCCCCCCEEEEEEECC
986034227643256777631221025764202238984999998079994407999975987999999986414893799888868858999990004898549971899982
68888886167358639

>ID5_P.chaetifer
MALVERLFPVMRPDFNFFDDAFVNMQLQMEEMEKRIEKMRQSMFELQPRDEFSDVMKNFMQVGRPVVEEKGQSKLKLEFDVKQFKPEEVNVKVVGNNILQVHAKHHEKSGESV
VHREYYRQYQLPEGVNVDLLKPTLSKDGVLQIEAPAPALKPRERQLSIEYKSDEEK
CCCCCCCCCCCCCCCCCCCCCCCCCCCCHHHHHHHHHHHHHHHHCCCCCCCCHHHHHHHHCCCCCCEEECCCCEEEEEEEECCCCCCCEEEEEECCEEEEEEEEEEEECCCCE
EEEEEEEEEECCCCCCCCCEEEEECCCCEEEEEEECCCCCCCCEEEEEEECCCCCC
996323234456640012343454225646777766776444313578641012333321257752233169739999981699954079999879499999999986317986
99999999958999991204899649970899981799988861676896267789

>ID1_P.australis[*Phoronis australis*]
TRGFQQEGDQVKLRMDLSAYKPDEIAVKVDNHHLKVHAKHEDEHSFRESRQNVLLPRGMDERALKSSLSPDGVLTFSAPAIAALPESGRSLAIEQEEPMAVESGSKK
CCCEEEECCEEEEEEEECCCCCCEEEEEEECCEEEEEEEEEECCCCCEEEEEEECCCCCCHHHEEEEECCCCCEEEEEECCCCCCCCCCEEEEEEECCCCCCCCCCC
97359839848999981899954089999899999999995335441699999689999911248874499708999835888777881687886046555543479

>ID4_P.australis[*Phoronis australis*]
MSYLSVFDRHVPILRRHYGYFDHLPDVWREERRNWQMSNIDEEIRRIKSKMFMLDPLEGTSSKSLTLGNDRVVDRWGKDGKDLKIEFDVQKFAPDEIEISTKNNMMTVHGKHK
EEKQEGNCYSSIYREFSKQFVLPAGTSAEEMQAELHKDGVLTVQAPVRIPVTHK
CCCCCCCCCCCCCCCCCCCCCCCCCCCCCCCCCCCCCCCCCCHHHHHHHHHHHHHHCCCCCCCCCCCCCCCCCCCCCCCEEEEECCEEEEEEEECCCCCCCEEEEEECCEEEEEEEEE
EEECCCCCEEEEEEEEEEEEEECCCCCCHHHEEEEECCCCEEEEEECCCCCCCCC
997643234667555675434445322322246764005789999987564235767677652001245665302566098799999816999540799998999999999975
333058825899989999999689999111488854996089998089876689

>ID3_Spinther
MSLTPFDRHVPMTRGVDWFFRQPSVGLDNHSTMSPLIDPHITFRDQFRAMEQQMRSMELAMNSTFADMSHRGYPQLTLPHSIEHPFDAPILPTTTDPRTGERHLALKFDMHQF
APEEIMINTKDNLLTVTGKHEKTGAGGNHSYREYKRAILLPENTQLDKLHSSLSRDGVLSVEGPLPALPAPEKGEEVSSRAAHTPMAITHQ
CCCCCCCCCCCCCCCCCCCCCCCCCCCCCCCCCCCCCCCCCCCCCCHHHHHHHHHHHHHHHHHHHHCCCCCCCCCCCCCCCCCCCCCCCCCCCCCCCEEEECCCEEEEEEEECCC
CCCCEEEEEECCEEEEEEEEEEEECCCCEEEEEEEEEEECCCCCCCCEEEEEECCCCEEEEEEECCCCCCCCCCEEEEEEECCCCCCCCCC
99888755787656766654465300145678775434423342011232566657677653332233567765444664222221123346542037609859999981699
95517999989999999998553137974999999989958999991205899649960899981799876556525778971777542379

>ID10_Fauveliopsis_n.sp.
FDVRQFKPEEITIKTQDDTLSIHGKHEDKSDTHHVYNEYKRVVTLPDGVVPTTLKSALSPEGVLRIEAPIAAPLEAPKETTIPIQHETTPKSIKQ
CCCCCCCCCEEEEEEECCEEEEEEEEEECCCCCEEEEEEEEEEECCCCCCCCEEEEEECCCCEEEEEEECCCCCCCCCCEEEEEEECCCCHHHCC
999999943189999899999999998521478658987898985799999120589854997189998178887667862788886266224319

>ID5_B.viridis
MSDPTSTSPGIESPETPVPISLGGASGAWSQPPRHDLASSLMSMQRQMAEMENQMERMTEYFFQKPANSEQQRPMSAWMEDDFFGGPRHSMLPRRRLPGSMLRDAHARWFSED
PGFSHDLPAALPRNPSQGRMSLQTDQFPSEKQLVCPINTPGSSLANIFKDSSSLSSDPDFFGPVIVNDGGKRKIQLRFDVHQYKPEEIEVKTLGSRVTVHALHKEETDHNRVY
CEFQRVYTLPDGVDAHSLESSFSREGILTIDAPLPSAIPEEPLNTAIKIIHEKK
CCCCCCCCCCCCCCCCCCCCCCCCCCCCCCCHHHHHHHHHHHHHHHHHHHHHHHHHCCCCCCCCCCCCCCCCCCCCHHHCCCCCCCCCCCCCCCCCCCCCCCCCCCHHHCCCCCCC
CCCCCCCHHHCCCCCCCCCCCCCCCCCCCCCCCCCCCCCCCCCHHHHHHCCHHHCCCCCCCCCCCCEEECCCCCEEEEEEEECCCCCCCEEEEEECCEEEEEEEEEECCCCCEEE
EEEEEEEECCCCCCCCCEEEEECCCCEEEEEEECCCCCCCCCCCEEEEEEECCC
999888876555776554444345445556422345679999999999999986520100011356665333553001013333577654554455677410100210234566
532334122123355446554445678876555552126764145553122322445567775123216896499999926999760899998999999999975326894799
999999977999992305998549971899981788765457624774787189

>ID8_C.teleta
MSSDELVSTAFEKDVPVQRMTGGFFDPFFLGGDSSSDSTSDFGSSMMRMKRQMADMERQMERMFEQFARLEPISARSTRSATGRYPSWWDDDGHDASLLGWRRGPRGVWEGDL
ALPAATSVSPMSPHEEPSRLMLRQSSHDHQDAVAMPKPAGGVAREFGAPVVVDHAGARKLRMRFDVRQFKPEEIQVKTHDNKVMVKAVHKEESDTNQVFCEYQRMFTMPPEVK
PDTLTSALSPDGVLTLEAPMPCAIEEVPQETHIPITHE
CCHHHHHHHHHHHCCCCCCCCCCCCCCEECCCCCCCCCCCCCCCCHHHHHHHHHHHHHHHHHHHHHHHHHCCCCCCCCCCCCCCCCCCCCCCCCCCCCCCCCCCCCCCCCCCCCCHHH
CCCCCCCCCCCCCCCCHHHHHHHHCCCCCCCCCCCCCCCCCCCCCCCCCCCCEEEECCCCEEEEEEEECCCCCCCEEEEEECCEEEEEEEEEEECCCCEEEEEEEEEEECCCCCC
CCCEEEEECCCCEEEEEEECCCCCCCCCCCEEEEECCC
96357776665305653101476545301267643466432331457899999999999999999997345665567766667778877766787410024778778882230
467778876667664546666532111012323366753320111378732442069749999982699975179999899999999997533797499999999995899999
11048873599708999817887644577425510139

Cluster B2 – Multiple sequence alignments

PROMALS3D alignment

PROMALS3D multiple sequence and structure alignment server http://prodata.swmed.edu/promals3d/promals3d.php (Pei et al. 2008)

```
Conservation:                                      5     5 565   6 857  6   5    5                    5 5    89        5 65 5959   68
ID8_C.teleta                  1  --GGVAREFGAPVVVDHA--GARKLRMRFDVR-QFKPEEIQVKTHDNKVMVKAVHKEESDT----NQVFCEYQRMFTMPPEVKPDTLTSALSPDGVLTLEAP   93
ID5_B.viridis                 1  --LSSDPDFFGPVIVNDG--GKRKIQLRFDVH-QYKPEEIEVKTLGSRVTVHALHKEETDH----NRVYCEFQRVYTLPDGVDAHSLESSFSREGILTIDAP   93
ID17_A.lacteospumosus         1  IDVTVQEIKPAVPIVEEK--GETKLKLEFNVK-EFRPEDLEVKLLGNNILQVTAHREDKTES---GMSKICYLRKYSLPEGIDKDHIKPTLTKDGVLTIEAP   96
ID5_P.chaetifer               1  SDVMKNFMQVGRPVVEEK--GQSKLKLEFDVK-QFKPEEVNVKVVGNNILQVHAKHHEKSGE---SVVHREYYRQYQLPEGVNVDLLKPTLSKDGVLQIEAP   96
ID5_H.robusta                 1  EAQALAELNPAAPIVEEN--GETKLKLDFNVK-GFKPEEVQVKIVGDNVLQVRAETGTKTDS---GVSHRYYVRHYALPDGIKADQIKPTMSKEGVLSIEAP   96
ID9_E.fetida                  1  VDVMVQEIKPSVPIVEEK--GETKLKLEFNVK-EFRPEDLEVKLLGNNILQVTAHREDKNEH---GMSKICYLRKYTLPEGIDKEHIKPTLTKDGVLTIEAP   96
ID21_C.matritensis            1  VDVMVQECKPSVPVVEEK--GETKLKLEFNVK-EFRPEDLEVKLLGNNILQVTAHREDRTDS---GMSKMRYLRKYTLPEGIDKEHIKPTLTKDGVLTIEAP   96
ID5_Polygordius_n.sp          1  IDRNKGMIEVKRPIVESEIPGQGKLKLEFDVK-QFKPEEVEVKVMGNNMLQINALHEEKSDT---GYSRRQYCRQYLLPEGVHADKIQPSLTQDGVLTIEAP   98
ID8_L.gigantea__Lottia_gi     1  -------------ITQDH--EGRKFRLQFDVR-QFKPEEIMVKTEGQKLEIHAKHEEKGDN----KSVYSEYHRQYLLPNELKTDRLVSKLSRDGVLSIEAP   82
ID9_L.gigantea__Lottia_gi     1  -----MNRLRCPIVTKPD--GSKALELQLDLS-NYKPEEINIKTAGKELSVHAKHEEKTEN----SSVYQEFTRKFTLPEGVNPEKVESTLSKDGILTISGP   90
ID6_L.gigantea__Lottia_gi     1  -----MKKLRDPIVKKDD--GSEALELQLDVS-NFKPEEITVTTSGKQLSVHAKHEEKTGN----SSVYQEFNRSFTLPDGVKPDTVQSTLSKDGILSITGP   90
ID7_L.gigantea__Lottia_gi     1  ---TQMKRLRHPIVKRAD--GSEALELQLDLS-NFKPEEITVKTSGKELAVHAKHEEKSEN----SSVYQEYCRKFVLPDNVNPELVESTFSKDGILTITGP   92
ID1_L.gigantea__Lottia_gi     1  --DEHIRQLRHPLVTRPD--GTEALDLKIDLS-HYKPEEIHIRTVGHELSVHAKHEDKTDH----SSVYQEYSRKFSLPAHVDPEHVQSSLSRDGILRI---   90
ID10_Fauveliopsis_n.sp.       1  ----------------------------FDVR-QFKPEEITIKTQDDTLSIHGKHEDKSDT----HHVYNEYKRVVTLPDGVVPTTLKSALSPEGVLRIEAP   69
ID1_P.australis__Phoronis     1  ------------TRGFQQ--EGDQVKLRMDLS-AYKPDEIAVKVDNHHLKVHAKHEDEH--------SFRESRQNVLLPRGMDERALKSSLSPDGVLTFSAP   79
ID5_P.californica             1  --AIEPMPISHPIIDGGP--GNEKMELRFDVR-EFHPSEISVTTQGNMLAVRARHQEQSESGTHKSSSWKEYNRQYLLPTQVDPAKIKPNLSPEGILTIESR   97
ID9_N.dendritica              1  --MVEGFGFRNPVITKPD--GSKHLQLEFDVR-HFKPEELNIKTLDNKLVVHAKHQEESDD----SQVYREYQRQFLLPEGIELDKMKSVLSPEGVLTVEAP   93
ID4_N.dendritica              1  -------------------------QLQFNMR-CFKPEELTVKTVDNKLQVYAKHEEETDH----GVIFQEYRREFQLPEGMDLEQMKSKLSPDGILTVEGP   72
Cri21__C.riparius__G4m_2      1  --QSNVSDINSPLIQDDG--DNKVLKLRFDVS-QYAPEEIVVKTVDNKLMVHAKHEEKTDT----KSVYREYNREFLLPKNVNPESIRSSLSKDGVLTVDAP   93
Ce25abc__Caenorhabditis_e     1  --DPYLDNLKSPLIKDES--DGKTLRLRFDVA-NYKPEEVTVKTIDNRLLVHAKHEEKTPQ----RTVFREYNQEFLLPRGTNPEQISSTLSTDGVLTVEAP   93
ID3_Spinther                  1  --HPFDAPILPTTTDPRT--GERHLALKFDMH-QFAPEEIMINTKDNLLTVTGKHEKTGAGG---NHSYREYKRAILLPENTQLDKLHSSLSRDGVLSVEGP   94
ID4_P.australis__Phoronis     1  --KSLTLGNDRVVDRWGK--DGKDLKIEFDVQ-KFAPDEIEISTKNNMMTVHGKHKEEKQEGNCYSSIYREFSKQFVLPAGTSAEEMQAELHKDGVLTVQAP   97
ID3_O.vulgaris__Octopus_v     1  --KTNMLQVENPIVKDSE--GNKKLALRFDVS-NFKPDEICVKTTDNTLHIQAKHEEKTPQ----KQVYREFSRHYTLPKSVDPLKLSSILTKDGVLHIEAP   93
ID3_L.gigantea__Lottia_gi     1  -----------PFVTDLD--GNKKLSIRFDVS-QFKPEEISVRTMDNKLMIQAKHTEESPG----RKVYREFSKQYILPNKIDPVKLESILSQDGVLSIQAP   84
ID3_E.scotia                  1  --VTDNWRVDSPTFVEEN--GQRKMKLNFDMG-HFTPEEITVKTKDNNLIVQAKHEEKSDS----GHVFREYNKMYQLPEGVKLDEMKSFLTQNGSLCVEAP   93
ID1_Owenia                    1  --EATPAIVGMQHKDVDQ-DGIKLFQMHFDVS-KYSFDDMELKAVDNTLVVIHKKNERVGAR---KYKIREQSKQFVLPNGVDIG----------------   78
ID3B_P.australis_G1_ACD_      1  --RSQTKRASFTKESSPP--SGRKFVMEFSIGKEVQPGDINIHTQDGVVHVTADWQEERAVG--QPVKQKTFHKSVPLPVGVDPESVTAHFLDGGKLVLEAP   96
Consensus_aa:                    ...........s..s.....s.p.hphcFslp.p@pP--lpl+h.ss.l..hs.+p-cps........bpp@.+ph.LPpshp.pplps.ho.-GlLslptP
Consensus_ss:                                 eeeee     eeeeeeee     hhheeeeee  eeeeeeeee          eeeeeeeeeee      hhheeeee    eeeee
```

MEGA 7 alignment (Kumar et al. 2016)

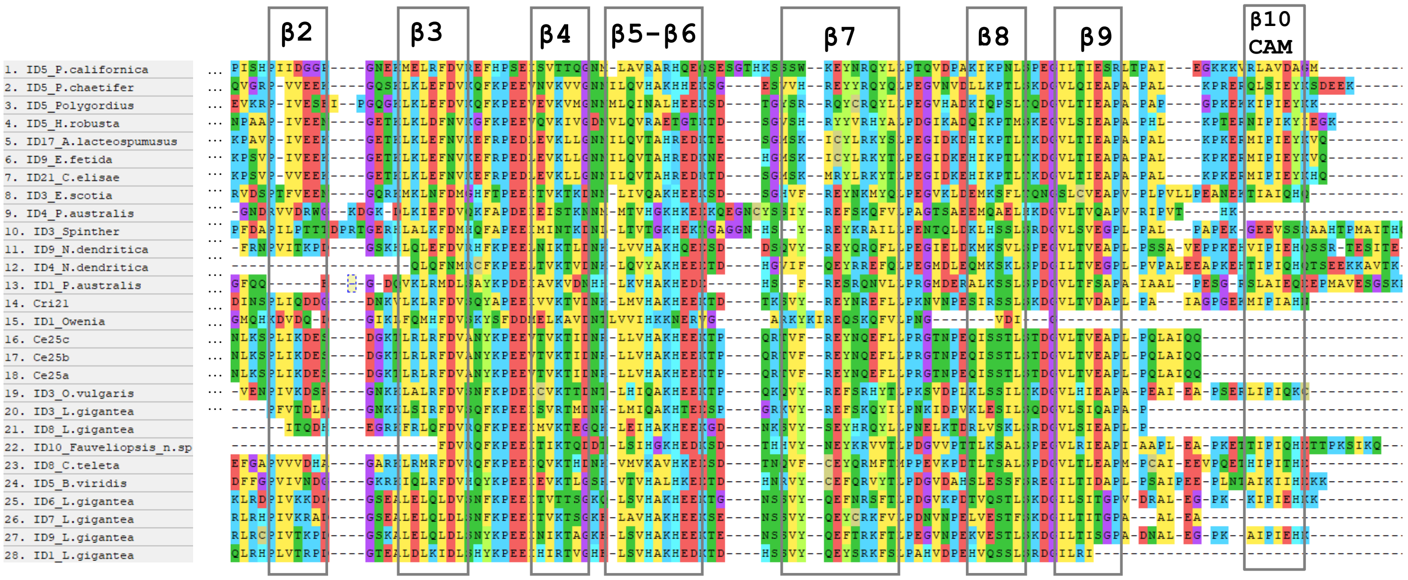

Cluster B3 – Phylogenetic trees

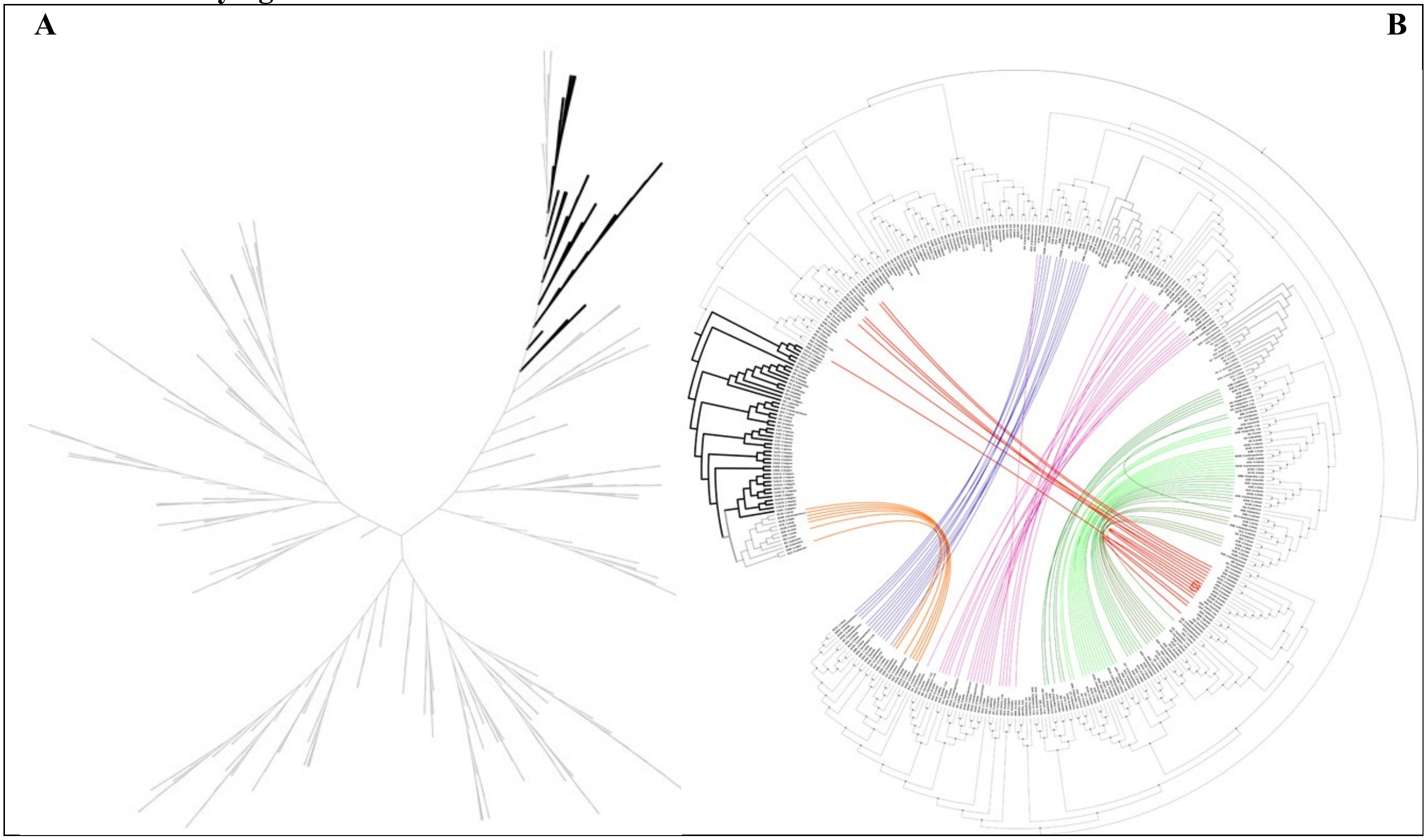

Supplementary Figure 13. A, unrooted ML phylogenetic tree, branch lengths not displayed. Small Hsp from cluster B3 in black. **B**, rerooted ML phylogenetic tree, small Hsp from cluster B3 in black and colored connection lines for the two corresponding ACDs of dimeric sHsps (not covered in this work).

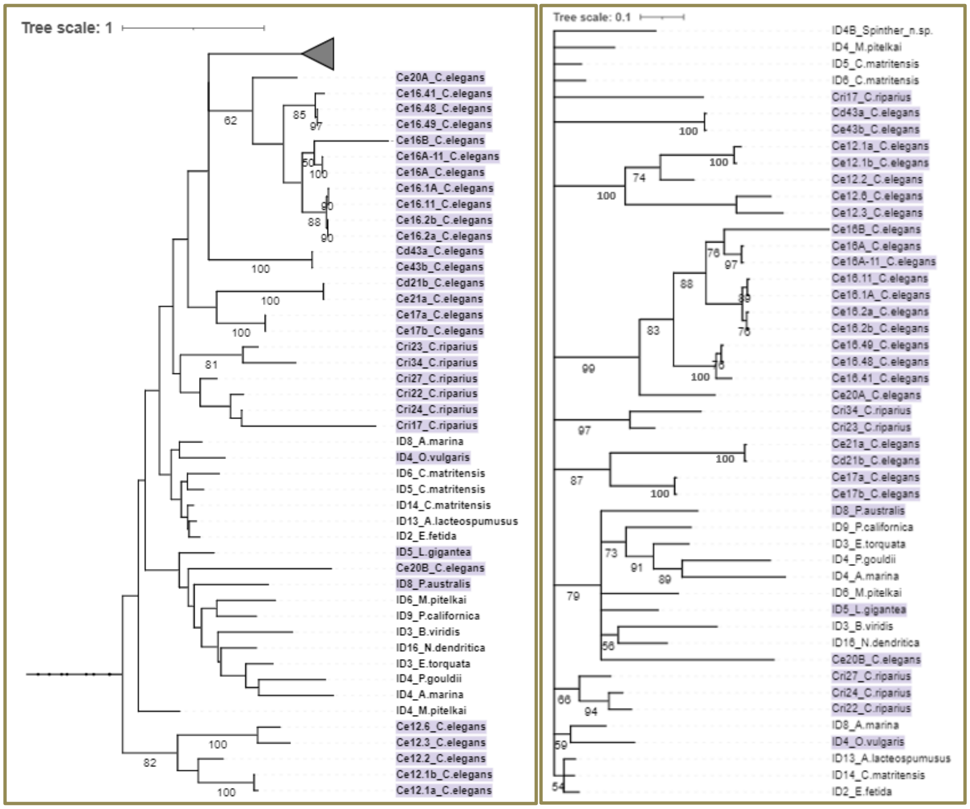

Supplementary Figure 14. Cluster B3. **Left,** clade recovered by ML analysis displaying branch lengths and bootstrap >50. **Right**, clade recovered by Bayesian Analysis displaying branch lengths and posterior probabilities in percentage.

Cluster B3 – Predicted Secondary Structures

Tool: PSSPRED online service (Yan et al. 2013) https://zhanglab.ccmb.med.umich.edu/PSSpred/
(**Bold black**: ACD domain delimited from the β2-strand to the β9-strand (Poulain et al. 2010); **underlined**: β3-β9 region, used in multiple sequence alignment for phylogenetic study; **H**:helix, **E**:strand, C:coil)

>ID13_A.lacteospumosus
MAGRLVAFDRPMFRRQLSGPWDMWSDPWSSDIGLPSRVLDQHFGLGLLDEDLMPPQMWRGLMVRPRR**QPAQQASGMSEVQNTDKDFRVNLDVSHFAPEELSIKTVDNRVIIHG**
KHEEKQDEHGFIQREFRRTYVLPTDVDPNTVKSSLTADGVLSIKAPKKTQALPTERVIPIERMDVEPVSSPESK
CCCCCCCCCCCCCCCCCCCCCCCCCCCHHCHCCCHHCHHHHHCCCCCCCCCCCCCCCCCCCCCCCCCCCCCCCCCCEEEEECCCEEEEEEECCCCCCCCEEEEEECCEEEEEE
EEEEECCCCCEEEEEEEEEEECCCCCCCCCEEEEECCCCEEEEEECCCCCCCCCCEEEEEEECCCCCCCCCCCC
98776544588767888887432445201001210101133248999852136776678654562001112468743668729928999973899954179999899999999
9743136882799999999995899999010489854997089998079887777527878970675544441259

>ID4_A.marina
MARVLQQTWRVLRPGISRLCSTKPPSNGVSRRQYSWGLGPGRIHPFGRHRFFADDIVNDVKRQVKWNLRNMENLMDNFLPARCFPESV**NVASRDMKIYQTDKLFEVRLNVSQF**
SPEELTVRQSGSRLIITGSHKQESEGQSLSREFTREITLPEHVEQDSLESVFTGSGQLILRGKVKDSPPENPDTVIKIQRTDPKPDGS
CCCCCCCCCCCCCCCCCCCCCCCCCCCCCCCCCCCCCCCCCCCCCCCCCCCCCCCCCCCHHHHHHHHHHHHHHHHHHHCCCCCCCCCCCCCCCCEEEEEECCEEEEEEEECCC
CCCEEEEEEECCEEEEEEEEEEEECCCEEEEEEEEEEECCCCCCCCEEEEEECCCCCEEEEEECCCCCCCCCCEEEEEEECCCCCCCC
985212551102322456667765335555554444678765455546654211122311212355566888876420565556521235665425586099499999980799
95407999989999999999874069759999999999848999990104898549970899983688877678626867861676 5689

>ID8_A.marina
TSGASEVAMTDKDFKVNLDVSHFKPGEITIKTVDNRVVVHGKHEERPDEHGFIQREFKRSYPLPEDVKAELVTSSLSADGI
CCCCCEEEECCCEEEEEEECCCCCCCEEEEEEECCEEEEEEEEEEECCCCCEEEEEEEEEEECCCCCCHHHEEEEECCCCC
998605787198489999818999632899998999999999976641699569999999999958999991123897279999

>ID3_B.viridis
MFRSVIPRANSCLRHVNSSAYRGFCSRPRRLTLTMMPTRGWFGDEVMKLMEGHSRRMDRMMEDMSRPWLKAFDKWPSLNFE**YGDTKNIQVTHNKNHFEVKLDVENYEPDDLEV**
KVVDRQLTISGKCEQKSDKVGLVSQEFTRVLQIPEDVDSESLETLLTSDGYLVVSGKVTSTEPESRRIEIKRPLNNSTDTK
CCCCCCCCCCCCCCCCCCCCCCCCCCCCCCCCCCCCCCCCCCCCCHHHHHHHHHHHHHHHHHHHCCCCCCCCCCCCCCCCCCCCCCCEEEEECCCEEEEEEEECCCCCCCEEE
EEECCEEEEEEEEEEECCCCCEEEEEEEEEEECCCCCCCCEEEEEECCCCEEEEEEECCCCCCCCEEEEEEEECCCCCCCC
98665567764323444433332224455442012566778874113341678878888875411433343343211000235765336787099599999816999541799
998999999999854216896589999999996899999010589864997189997078888775267468751787 7789

>Ce20B_C.elegans [*Caenorhabditis elegans*]
MHHVDFDDDDLDSYLEGSVWSEKPSASEIFSSKPGDVISVFPRSSHSNMIYGYPINYKETVFPTHRGPPDQSYDSYYTKITESRPRSP**GPVAGAGEITNTSHGFTIEIDVFHF**
MPEEIKVVLTDDTLSISGERFESTGDGHTLRRSFSRKYSIPDDVHLDTIRSHLTNSGVLIINGSRKGWRETSISSYHPTTQRNVARSVTSIV
CCCCCCCCCCCCCCCCCCCCCCCCCCCCCCCCCCCHHHHHCCCCCCCCCCCCCCCCCCCCCCCCCCCCCHHHHHHHCCCCHHHHCCCCCCCCCCEEEEECCCEEEEEEEECCC
CCCEEEEEEECCEEEEEEEEEEECCCCCEEEEEEEEEEECCCCCCCCEEEEEECCCCEEEEEEECCCCCCCCCCEEEEEEECCCCCCCCCCC
98678987654211356531235456532354643023103000233211347876554556411254134444431663211012334456633568719828999981599
976189999899999999997421368965999999888589999900048996399718999817998766765144367526776677889

>ID5_C.matritensis
MAGRMVSFDYPFGSRRRSLIPWDLWHDPWHSDLELPRRLFEQHFGLGLWDDDLFPPEIWKGLVFRPKRQPT**QQASGVSEVANTDKKFQVNLDVSHFRPEELSIKTSGNRVIIH**
GKHEEKQDEHGFIQREFRRTYVLPNDVDPETVKSSLSGDGILAVVAPKKAIEKSNERDVPIERMETEPSPPQAS
CCCCCCCCCCCCCCCCCCCCCCCCCCCCCCCCCHHHHHHHHHHCCCCCCCCCCCCCCCCCCCCCCCCCCCCCCCCCCEEEEECCCEEEEEEECCCCCCCCEEEEEECCEEEEE
EEEEEECCCCCEEEEEEEEEEECCCCCCCCEEEEEECCCCEEEEEEECCCCCCCCCEEEEEEECCCCCCCCCCC
98765651124677764334555567752120101333333214899984224788778876567531100135774356762982899996489995417999989999999
9974203798279999999999589999912069996499708999807888777853787886168764434 79

>ID6_C.matritensis
MAGHLIAFEYPSMRRMPGFGFYDLASDPWDLEPSWPSLFCPTTVDDIVLRHLHPAHSALHGRRRKLRKSMMT**GRKGASSQEVRQQKEEEDFSVRLDVSHFQPEELTVKTTGNR**
VIVHARHEEKQDEHGYIEREFTRTYLLPEDVDPDTVKSCLSGDGVLSIEAPKKRRLEASTERVIPIEHMEVETTSEDQGQK
CCCCCCCCCCCCCCCCCCCCCCCCCCCCCCCCCCCCCCCCCCCCCCCCCCCHHHHHHCCCHHHHHHHHHHCCCCCCCCCCCEEEEECCCEEEEEEEECCCCCCCEEEEEECCE
EEEEEEEEEECCCCCEEEEEEEEEEECCCCCCCCEEEEEECCCCCEEEEEECCCCCCCCCCEEEEEEECCCCCCCCCCCCC
99876554463010134544357788877766565445654442001241245430110111012211003455777652355652984899998169994417999989999
99999974203689669999999999589999911159985399708999817988777786278888615666454 20149

>ID14_C.matritensis
MAGRLIAFDRPMFRRQMSGPWDLWSDPWSSDLALPNRLLDQHFGLGLLDEDLFPPQLWKGLVVRPRRQPA**QQASGMSEVQNTDAAFHVNLDVSHFAPEELTIKTADNRVMIHG**
KHEEKQDEHGFIQREFKRTYVLPNDVDPNTVKSSLSADGVLSIKAPKKTQALPQERVIPIERMDAESTPSPASK
CCCCCCCCCCCCCCCCCCCCCCCCCCCHHHHCHHHHHHHHHCCCCCCCCCCCCCCCCCCCCCCCCCCCCCCCCCCCEEEEECCCEEEEEEEECCCCCCCEEEEEECCEEEEEE
EEEEECCCCCEEEEEEEEEEECCCCCCCCEEEEEECCCCEEEEEECCCCCCCCCCEEEEEEECCCCCCCCCCCC
98766545688656888878543445211011012221111158999831126776678754453222222468743557549839999981699964179999899999999
9731026897699999999994899999010589854997089998189887777538878971665556523 69

>ID2_E.fetida
MAGRLVAFDNPMFRRQMSGPWDLWSNPWNFDVALPSRLLDQHFGLGLLEEDLFPPQVWRGVVVRPRRQPA**QQASGMSEVQNTDKDFHVNLDVSHFSPEEISIKTVDNRVIIQG**
KHQEKQDEHGFIERQFKRTYVLPNDVDPNTVKSSLSADGVLSIKAPKKAQEVPTERVIPIERMDAEPAQSPESK
CCCCCCCCCCCCCCCCCCCCCCCCCCCCCCCCCCCCCCCCCCCCCCCCCCCCCCCCCCCCCCCCCCCCCCCCCCCCEEEEECCCEEEEEEECCCCCCCCEEEEEECCEEEEEE
EEEEECCCCEEEEEEEEEEEECCCCCCCCEEEEEECCCCCEEEEECCCCCCCCCCEEEEEEECCCCCCCCCCCC
98875434688766888888632367544443210122200158999831136776688743453112212468742557619928999973899964179999899999999
9853037971899999999995899999010589855997189998079877777628878863776422 20149

>ID3_E.torquata
MFRQVVPRVMRNIAEMRPLTKRYYCGRSRWVPVRRRFFDDFWPSPLSRVMDRQMNQMWKTMDDMSRFWEPYEYGPAAARG**SRPSKDVQVKYTDQVFEVRLDVNQFAPEELNIK**
VGGERLTITGKQQERPDEHGVISREFMRQFAIPENVETESFESQLTTDGFLVIKGKVKGAEDVTERVINITREEPAPAKEPSKEEGSPNPAT
CCCCCCCCCCCCCCCCCCCCCCCCCCCCCCCCCHHHCCCCCCCCCHHHHHHHHHHHCCCCCCHHHHHHCCCCCCCCCCCCCCCCCCEEEEECCCEEEEEEEECCCCCCCEEEE
EECCEEEEEEEEEEECCCCCEEEEEEEEEEECCCCCCCCEEEEEECCCCCEEEEEECCCCCCCCCEEEEEEECCCCCCCCCHHHHHHHCCCC
97434763247753246664335766556762011012446777302145554400213200012432321246421124678864146762983899999259997508999
989999999999854216894699999988858999992315999549971899982688877787178777527753332102321001379

>ID5_L.gigantea[*Lottia gigantea*]
LNKKEFEVNVDVHHFESENIHVKLSNNRLTISGKHESKQDEHGYVSREFSREFTVPENVDAETMTSSITEEGVLVIRAKVKPEENPKDKTVNIEIERDNTSSGSTEDKK
CCCCEEEEEEEECCCCCCEEEEEEECCEEEEEEEEEEECCCCCEEEEEEEEEEECCCCCCHHHEEEEECCCCCEEEEEECCCCCCCCCEEEEEEECCCCCCCCCCCCCC
999708999991699943079999899999999998420268946899999999958999990005898439970899982688866775378578615787655512239

>ID4_M.pitelkai
MAIRPIDIERPFRRMRSWIDDPWADMKDPHRMFDQFFGQSLSNDDFVGPPVLFRGHYMRPRRLDKILEKYGNLSELHTTKDPEVFK**ALKQTGLAEVKCDDSQFSVMLDVTQFS**
PEEVEVKTNDNKLTIHAKHEEKEDEHGFISREFTRTYLLPKNVDPLTLTTKLTSDGVLNIAAPKKNALPPKERILPIKME
CCCCCCCCCCCCCCCCCCCCCCCCCCCCCHHHHHHCCCCCCCHHHCCCCCCCCCCCCCCCCHHHHHHHHCCCCCCCCCCCCCHHHHCCCCCCCEEEEECCCEEEEEEECCCCC
CCCEEEEEECCEEEEEEEEECCCCCCCEEEEEEEEEEECCCCCCCCEEEEEECCCCEEEEEEECCCCCCCCCEEECEEEC
97421356787444675323565556773011010247999913327786445874224311224554303532334455411220124677447887298599999828999
540799998999999999873024798079999998996799999231699964997189998079887777615503639

>ID6_M.pitelkai
MEKEMSRLFDDLQHSNRFRPSFFRPWGGRREQP**ASINQDVEVKYNEKEFSIKLDVQDYSEGELKVKVVEDRVVVSGKVQEKQDEHGFVSQEFSRQFLIPENVDPETIESSICA**
EGVLTIQGQVTGAENIEEREIPINKEPPKETKND
CHHHHHHHHHHHCCCCCCCCCCCCCCCCCCCCCCCCCCCEEEEEECCEEEEEEEECCCCCCCEEEEEECCEEEEEEEEEEECCCCCEEEEEEEEEEECCCCCCCCEEEEEECC
CCCEEEEEECCCCCCCCCEEEEEEECCCCCCCCC
35689998875337754542002554332112224577514787099599999816999541799998999999999742115883799999999828999991115899629
9708999826887777872786764377421279

>ID16_N.dendritica
MFRTLVPRLARCAYETVQPGSGRIQHHAVPIRSYCSRWDRRVPMYFRRRNLLEDFFDRSHNDLAWPARMVDRWMKHNYRTMEDMMNPRFFDKFFNRYDVVR**TGAAREIEVKYN**
DKHFEVKLDVNNFSPEEVNVKLQGDRLIVGAKHEEKRDDYGFVSRELSRQFSVPEDVDAESLESVLTNDGYLVIHGKVKGAEAKPERILNVQREAPKEDQSKDPERSD
CCCCCCCHHHCCCCCCCCCCCCCCCCCCCCCCCCCCCCCCCCCCCCCCCCCHHHHCCCCCCCCCCCCCHCHHHHCCCCCHHHHHCCCCCCCCCCCCCCCCCCCCCCCEEEEEE
CCEEEEEEEECCCCCCEEEEEEECCEEEEEEEEEEECCCCCEEEEEEEEEEECCCCCCCCEEEEEECCCCCEEEEEECCCCCCCCCEEEEEEECCCCCCCCCCHHHCC
98644642333567766666765445777655554666556774347863113312776554446400002330665221233225411132346532223456764256870
984999998169996349999989999999999975227970799999988858999991215999649970899982688776787067567505632123202215 9

>ID9_P.californica
MFRAVIPRVCNASRQALRPVGARSYYKRHHWVPMTREGRSLIDDFFGDRFPRWALKVADHQMRNMERRMEDMVGPWYVRHGSR**RVGSKQVEVKYDDKVYEVKVDVHEFSPEEL**
NVKIVEDRLVISGKLEDRPDDHGFISREMKREFIIPENVEVETIESHLTDEGFLKICGKVKGGDEAASERVINITREPPKEGELPKEGESPKEGDTK
CCCCCCCCCCCHHHCCCCCCCCCCCCCCCCCCCCCCCCCCCCHHHCCCCCCCCCCCCCHHHHHHHHHHHHHCCCCCCCCCCCCCCCCCCEEEEECCCEEEEEEECCCCCCCCE
EEEEECCEEEEEEEEEEECCCCCEEEEEEEEEEECCCCCCCCCEEEEECCCCEEEEEEECCCCCCCCCCEEEEEEECCCCCCCCCHHHHHHHHHHCC
98643234772110024666678865677888888875211222158988852135323444344544321036632221000246764256860985999998379997508
99998999999999732036881699999999984899999110589964997189998179886667870687886266432332012334443319

>ID4_P.gouldii
MVIRSVIPRVQQITSRALIPCRQYCSRRGPRLVPMWGPWRDHWPGTSLVGRFFNDPPSMFAQQFKGIEKIMDRFMSPRWWDGDFPRH**RCRGREMEVKYDRKVFEVRLDVSLFT**
PEDIKVTVNESDRILVISAKHKEKPDDHGCISREFNRQILIPENVEMEEFQSLLTNDGHLVVRAKVEGAEEIKERVIDIQKEEPKEDEKTKEKD
CCCCCCCCCCCCCCCCCCCCCCCCCCCCCCCCCCCCCCCCCCCCCCCHHHHHHCCCHHHHHHHCCCCHHHHHHHHCCCCCCCCCCCCCCCCCCEEEEEECCEEEEEEEECCCC
CCCEEEEEEECCCEEEEEEEEEEECCCCEEEEEEEEEEEECCCCCCCCEEEEEECCCCCEEEEEECC CCCCCCCEEEEEEECCCCCHHHHHHHC
98655554310134432246520115778644555565312478743135651764113332113313344554166567775332114787425787199599999816999
5408999997399899999997320368838999999989848999991216998539970899982688876686178667437641223454 19

>ID8_P.australis[*Phoronis australis*]
MSYRRVFQDLFRRSLCSARQYQLRRGFRDTQCLQNEGGHGRGRWLYPRWGPGGSWFSGNRAFPFGFMREAAKVMEEMQRDLDRAFPARHGYGPGWWGPRHGVI**SAESALPEIK**
FTDGEFRVRVDVQHYTPEEIGVKIADNTIKISGKHETKHDDYGMVAREFTREFSIPEEIDQESLECQISEDGFLTVMGSVKRSEQPKPRTLKIEREKGSEIKNDE
CCCCCCCCCCHHHHCCCCCCCCCCCCCCCCCCCCCCCCCCCCCCCCCCCCCCCCCCCHHHHHHHHHHHHHHHCCCCCCCCCCCCCCCCCCCCCCCCCCEEE
EECCEEEEEEEECCCCCCCEEEEEECCEEEEEEEECCCCCCCCEEEEEEEEEEECCCCCCCCEEEEEECCCCEEEEEEECCCCCCCCCEEEEEEECCCCCCCCCC
98766666411321134111111124345765434577777655567778875568876677755344267888899876564225677888743355311123457642168
70995999998079994417999989999999997411126785589999999984899999111589854997089998268876667548867863753233479

>ID4_O.vulgaris[*Octopus vulgaris*]
MASPHYPYSRSLTPLYNRMPSWSIWEDPWTDMPPFSRLSDQNFGISPMMDDFFSHWQNWRRSNMHSGFHHSMPVAQQH**SAVSTGFSEVSNDEKEFKVRLDVSHFNPDEITLKV**
IDNRIVIHAKHEEKQDEHGYIQREFKRQYLLPRECDAANVTSMLASDGVLTIRASKLALNSGERVIPITQETSSANL
CCCCCCCCCCCCCCEECCCCCCCCCCCCCCCCCCCHCHHHCCCCCCCCCCCCCCCCCCCCCCCCCCCCCCCCCCCCCCCCCCCCCCEEEEECCCEEEEEEECCCCCCCEEEEEE
ECCEEEEEEEEEEECCCCCEEEEEEEEEEECCCCCCCCCEEEEECCCCEEEEEEECCCCCCCCCEEEEEECCCCCCC
99888887542220002788865445774447741001111258887720022222112566455654566642001223356642568629918999973899975189999
89999999998531279827999999999589999911048985499708999826899888715778970676559

>Cri17[*Chironomus riparius*]
MDCPRRSRDRTLSPLRSPSLQRHLMDNIDIP**YPEQFLTNHRPKSFKQLLIGKDGFFRTAVDIHDFTKEEVEVKTIGHTIMITAAHDEKEDEFGHIERRFSRKYVLPLDLDIEK**
ISSWISKGVLYIKVPPTMNPVEHRIIDIKLIEDKE
CCCCCCCCCCCCCCCCCCHHHHHHHHHHCCCCCCCCCCCCCCCCCCCEEEECCCEEEEEEECCCCCCCCEEEEEECCEEEEEEEEECCCCCCCEEEEEEEEEEECCCCCCCCE
EEEEEECCEEEEEECCCCCCCCCCEEEEEECCCCC
99988888643454357144446542310435553223456666531244159859999981799943079999899999999960224789648998999985899999110
489961984899972899888870685797377 79

>Cri22[*Chironomus riparius*]
MSMCPFGFSPFEMDGLEPRFGQGIAHSDFFSDRLVPRAGMHSLLTGYKRPWAINHSAKSMIKDQKNLHFGKDGFQASVDVHHFQPSEITVKTIENQIVVEGRHEERDDGHGSI
QRHFVRKYVLPKEYDMNSVHSTLSSDGVLTIKAPPPPAISGTSEKHVTITHTNIPAHLNIKEHPKSPQIQHDNGKSPAVNKP
CCCCCCCCCCCCCCCCCCCCCCCCCCCCCCCCCCCCCCHHHHHHCCCCCCCCCCCCCCCCCCCCCCEEECCCEEEEEEECCCCCCCCEEEEEECCEEEEEEEEEECCCCCCEE
EEEEEEEEECCCCCCCCEEEEEECCCCCEEEEECCCCCCCCCCCEEEEEEECCCCCCCCCCCCCCCHHHHHHHHHCCCCCCC
997666778764101345678999912324653244320122111124652233311113467531266198289999648999761799998999999999973102689659
9999878867999991105898549970899980798767778717878971654322321122212333321111100179

>Cri23[*Chironomus riparius*]
MRGLPEFLNIIDEIQQALNEPAKPTFAQQAYPMVFFEPHPITFRTFTTPIIRPSNHFCPLLIKKASQNDLSTKSKEQEVKDFSITFDFSSFKPEEISVKVKDRDVIIEAKHEE
REDEHGFISRQLTRKFILPDEYDLDTISTYLNADGKMTIKALKLRPEIEVKERIIPIKRLSESVDGQEDKEALDVKKTKDVEE
CCCCCHHHHHHHHHHHHHCCCCCCCCCCCCCCCCCCCCCCCCCCCCCCCCCCCCCCCCHHHHHCHCCCCCCCCEEEECCCEEEEEEEECCCCCCCEEEEEECCEEEEEEEEEE
CCCCCCEEEEEEEEEEECCCCCCCCCEEEEECCCCCEEEEEECCCCCCCCCCEEEEEEECCCCCCCCCCHHHHHHHHHHHHHH
99874244588999876416556654456776555787545655343610122134211332000025887421343398289999927999441799998999999999742
05788369999999899589999911048985599718999816887767787178887526643233212333445555540

>Cri24[*Chironomus riparius*]
MALNPFQAFDIFEDPFFHHGMEPRFGMGIMPHDFSSHRILPRQGNHHLMTGYRRPWQLAREAVGELAKDQKNLHFGKDGFQACVDVHHFAPSEITVKTVDHTVTIEGKHEERE
DGHGSVQRHFVRKYVLPKEYDMNNVHSTLSSDGVLTIKAPPPAIEGASERHIPITHTNAPAHLSVKHNKKHIDDQPKEDGEASNGN
CCCCCCCCCCCCCCCCCCCCCCCCCCCCCCCCCCCCCCCCCCCCCCCCCCCCCCCHHHHHHHHCCCCCCCCCEEECCCEEEEEEECCCCCCCCEEEEEECCEEEEEEEEECCC
CCCCEEEEEEEEEEECCCCCCHHHEEEEECCCCCEEEEECCCCCCCCCCEEEEEEECCCCCCCCCCHHHHHHHHHHHHHHHCCCCC
99875535776456774333344568898811036775565676654445345613555421202478875316509838999981799976189999899999999971024
78855999899888489999921158862599718999716888777871787786267532121101222334445543201369

>Cri27[*Chironomus riparius*]
MSLVPTLWRDIDDFYANPWRLQHDLIPSWRRPMSFFDDFGFGFHPHDLNEMLRIPREFRQMEQRSRELQRQMMGDINEVIPTTGKDGFQVCMDVQQFKPSEITVKTTDNSVVI
EGKHEERQDEHGQISRHFIRRYTLPKGYDPKSISSELSSDGVLTIKAPPPQAIENNERVISIQHTGPARLNVKENKPIEDKK
CCCCCCHHHHHHHHHHCCCCCCCCCCCCCCCCCCCCCCCCCCCCCCCCCCCCCCCCCHCHHHHHHHHHHHHCCCCCCCCEEECCCCEEEEEEEECCCCCCCEEEEEECCEEEE
EEEEEECCCCCEEEEEEEEEEEECCCCCCCCEEEEEECCCCCEEEEEECCCCCCCCCEEEEEEECCCCCCCCCCCCCHHHCC
99766212122234316511222100123577663113588998121265202542100123235565431147887631220598389999935999761899998999999
9998420479849999999788489999912058984499818999806888777870786675045543443221033259

>Cri34[*Chironomus riparius*]
MALLPVLLDMVDDIYENLDNPENTMGDIPLLFVPHGRRRGRDCPRRMEQSDGECPRNKEHSDVEGHGHRHCHGGEKHGKHGHGHGEGHKHAHSHGEGHKHAHGHGQELGLGQE
EKRDESHGRRHCRRKERRGAEEGHCRFAELRKGLFGMRGWENCPRFNGKGCPRFAGRGGCPCKKTLAEDFKVTFDVKSFNPEEISVKVKGREIFVDGKHDERADEQGFVSRQF
TRRFIVPDEFDTDTIATYLDIEGRMTIVASKPKPPVDDSTERIIPIERVAKVQDEVVDLSESSDTDKMDEDTNDSKKN
CCHHHHHHHHHHHHHHHHCCCCCCCCCCCEEEECCCCCCCCCCCCCCCCCCCCCCCCCCCCCCCCCCCCCCCCCCCCCCCCCCCCCCCCCCCCCCCCCCCCCCCCCCCCCCCC
CCCCCCCCCCCCCCCCCCCCCCCCCHHHHHHHHHCCCCCCCCCCCCCCCCCCCCCCCCCCCCEEECCCEEEEEEEECCCCCCCEEEEEECCEEEEEEEECCCCCCCCEEEEEE
EEEEECCCCCCCCEEEEEECCCCCEEEEEECCCCCCCCCCCEEEEEEECCCCCCCCCCCCCCHHHHHHHHHHHCCCCC
96156889987999986147644456641465237766566666543335787776555666667777665446666677766666565543345555566667653124565 4
44444444443211001333234310355443320456654565566666654457888640354498079999927999541899998999999998511147896378778
888857999991206999569970899981699987668861787887045200111111001233223332000149

>Ce12.1a[*Caenorhabditis elegans*]
MHTIPITTDSAASWDWPLQHNDGVVKVTNTSEKFEVGLDAGFFGPNDIDVKVNGIEIIIHLRHDLLQNRPTEYGIVNREVHRTYKLPEDVDPSTVRSHLNSSGVLTITANKL
CCCCCCCCCCCCCCCCCCCCCCCCEEEEECCCEEEEEEECCCCCCCEEEEEEECCEEEEEEEEEECCCCCCCCEEEEEEEEEEEECCCCCCCCEEEEEECCCCCEEEEEECC
9988888776665532010489844899739828999980699975089999899999999982001036896499999999968999991105898449971899 98449

>Ce12.1b[*Caenorhabditis elegans*]
MHTIPITTDSAASWDWPLQHNDGVVKVTNTSEKFEVGLDAGFFGPNDIDVKVNGIEIIIHLRHDNRPTEYGIVNREVHRTYKLPEDVDPSTVRSHLNSSGVLTITANKL
CCCCCCCCCCCCCCCCCCCCCCCCEEEEECCCEEEEEEEECCCCCCCEEEEEECCEEEEEEEEEEECCCCCEEEEEEEEEEECCCCCCCCEEEEEECCCCCEEEEEECC
9998888776654433100479844899649928999980699975089999899999999985422689738999999996899999220599854997189998449

>Ce12.2[*Caenorhabditis elegans*]
MSAIEVTADAASTWDWPLQHNDGVVKVHNTKEKFEVGLDVQFFTPKEIEVKVSGQELLIHCRHETRSDNHGTVAREINRAYKLPDDVDVSTVKSHLATRGVLTITASKKA
CCCCCCCCCCCCCCCCHHHCCCCCEEEEECCCEEEEEEEECCCCCCEEEEEEECCEEEEEEEEEEECCCCCEEEEEEEEEEECCCCCCCCEEEEEECCCCEEEEEEECCC
99888766776666531215898458997499189999816999750899998999999999754205882799999999968999991205999649971899972699

>Ce12.3[*Caenorhabditis elegans*]
MSVAIDHDQTAKWDWPFQKGDGVVKVLDYEDHFEVGLEAHNFLPNEIDVKNIGEFLEIHMAHTTKDDKFGSITRSITRCYRLPKGTDPATIKSKLDGSGILHISGNKKK
CCCCCCCCCCCCCCCCCCCCCCCEEEEECCCEEEEEEEECCCCCCCEEEEEECCEEEEEEEEEEECCCCCEEEEEEEEEEECCCCCCCCCEEEEECCCCEEEEEECCCC
9987555655543220104898627897398289999806999651899998999999998644226997489989999958999991204898379961899970799

>Ce12.6[*Caenorhabditis elegans*]
MMSVPVMADEGTKWDWPLQKGDGVVNVLDDDDHFEVGLEAHNFLPKEIEVKNIGELLEIHMEHNVKKDSFGDVSRNITRCYKLPKNVDMKTIKSNLDSHGILHIEARKMH
CCCCCCCCCCCCCCCCCCCCCCCCEEEEECCCEEEEEEEECCCCCCEEEEEEECCEEEEEEEEEEECCCCCEEEEEEEEEEECCCCCCCCEEEEEECCCCCEEEEEECCC
99988777776554431014887458997399289999816999650899998999999999863312689748999989968999992316998559971899985289

>Ce16.11[*Caenorhabditis elegans*]
MSLYHYFRPAQRSVFGDLMRDMAQMERQFTPVCRGSPSESSEIVNNDQKFAINLNVSQFKPEDLKINLDGHTLSIQGEQELKTEHGYSKKSFSRVILLPEDVDVGAVASNLSE
DGKLSIEAPKKEAIQGRSIPIQQAPVEQKTSE
CCCCCCCCCCCCHHHHHHHHHHHHHHCCCCCCCCCCCCCCEEEEEECCEEEEEEEECCCCCCCEEEEEECCEEEEEEEEEEEECCCEEEEEEEEEEECCCCCCHHHEEEEECC
CCCEEEEEECCCCCCCEEEEEEECCCCCCCCC
99886666421002336664543322011100035677643689829969999980699954179999899999999999751278377799999977999991125888449
98189998178888861687764476301159

>Ce16.1A[*Caenorhabditis elegans*]
MSLYHYFRPAQRSVFGDLMRDMAQMERQFTPVCRGSPSESSEIVNNDQKFAINLNVSQFKPEDLKINLDGHTLSIQGEQELKTEHGYSKKSFSRVILLPEDVDVGAVASNLSE
DGKLSIEAPKKEAIQGRSIPIQQAPVEQKTSE
CCCCCCCCCCCCHHHHHHHHHHHHHHCCCCCCCCCCCCCCEEEEEECCEEEEEEEECCCCCCCEEEEEECCEEEEEEEEEEEECCCEEEEEEEEEEECCCCCCHHHEEEEEECC
CCCEEEEEECCCCCCCEEEEEEECCCCCCCCC
998866664210023366645433220111000356776436898299699999806999541799998999999999999751278377799999977999991125888449
98189998178888861687764476301159

>Ce16.48[*Caenorhabditis elegans*]
MLMLRSPFSDSNVLDHFLDEITGSVQFPYWRNADHNSFNFSDNIGEIVNDESKFSVQLDVSHFKPEDLKIELDGRELKIEGIQEKKSEHGYSKRSFSKMILLPEDVDLTSVKS
AISNEGKLQIEAPKKTNSSRSIPINFVAKH
CCCCCCCCCCCCHHHHHHHHHHCCCCCCCCCCCCCCCCCCCCCCCCEEEEECCCEEEEEEEECCCCCCCEEEEEECCEEEEEEEEEEEECCCEEEEEEEEEEECCCCCCHHHEEE
EECCCCCEEEEEECCCCCCEEEEEEECCCC
99877888874223567666423356876677642100246774256870994999998169995407999989989999999987349848999998983899999111488
854998089998279998705756751379

>Ce16.2a[*Caenorhabditis elegans*]
MSLYHYFRPAQRSVFGDLMRDMALMERQFAPVCRISPSESSEIVNNDQKFAINLNVSQFKPEDLKINLDGRTLSIQGEQELKTDHGYSKKSFSRVILLPEDVDVGAVASNLSE
DGKLSIEAPKKEAVQGRSIPIQQAIVEEKSAE
CCCCCCCCCCHHHHHHHHHHHHHCCCCCCCCCCCCCCCCCEEEEEECCEEEEEEEECCCCCCCEEEEEECCEEEEEEEEEEEECCCEEEEEEEEEEECCCCCCHHHEEEEEECC
CCCEEEEEECCCCCCCEEEEEEECCCCCCCCC
998633343102344455400211222233310236776426898299699999806999541799998999999999999751488478799999977999991124888449
97089998178888861687775267533689

>Ce16.2b[*Caenorhabditis elegans*]
MRDMALMERQFAPVCRISPSESSEIVNNDQKFAINLNVSQFKPEDLKINLDGRTLSIQGEQELKTDHGYSKKSFSRVILLPEDVDVGAVASNLSEDGKLSIEAPKKEAVQGRS
IPIQQAIVEEKSAE
CCCCCCCCCHHHHHHCCCCCCCEEEEEECCEEEEEEEECCCCCCCEEEEEECCEEEEEEEEEEEECCCCEEEEEEEEEEECCCCCCHHHEEEEEECCCCEEEEEEECCCCCCCEE
EEEEECCCCCCCCC
986022112011232047887537998399699999806999540799998999999999998640588478799999977999991125888449970899981789988616
87785267422589

>Ce16.41[*Caenorhabditis elegans*]
MLMLRSPYSDSNALDHFLDELTGSVQFPYWRNADHNSFNFSDNIGEIVNDESKFSVQLDVSHFKPENLKIKLDGRELKIEGIQETKSEHGYLKRSFSKMILLPEDADLPSVKS
AISNEGKLQIEAPKKTNSSRSIPINFVAKH
CCCCCCCCCCCCHHHHHHHHHHCCCCCCCCCCCCCCCCCCCCCCCCEEEEECCCEEEEEEEECCCCCCEEEEEEECCEEEEEEEEEEEECCCCEEEEEEEEEEECCCCCCHHHEEE
EECCCCCEEEEEECCCCCCEEEEEEECCCC
9988788887412257776641334588767763100025787426787098499999806999540899998999999999986411465589999999995899999111589
854997089998179998705757861379

>Ce16A[*Caenorhabditis elegans*]
MSVSPFFNPKRDSQLGEMMRDMGGMQRRLMPISGTFNPMTDDSEIMNSNDKFAVNLNVSNFKPEELKVNLEGRQLSIQGEHDVENEHGASRKSFSRMILLPEDVDITSVATNL
SNDGKLCIEAPKLEGVCGRSVPVKEASMDHHHIE
CCCCCCCCCCCHHHHHHHHHHHHHHHHHHCCCCCCCCCCCCCEEEEEECCEEEEEEEECCCCCCCEEEEEECCEEEEEEEEEEECCCCEEEEEEEEEEECCCCCCCCEEEEEE
CCCCCEEEEEECCCCCCCEEEEEEECCCCCCCCC
99753447520124445654544333320366544345566347898299599999816999541799998999999999975225882788999999689999900058886
3997089998368898861687886377422689

>Ce16A-11[*Caenorhabditis elegans*]
MNSNDKFAVNLNVSNFKPEELKVNLEGRQLSIQGEHDVENEHGASRKSFSRMILLPEDVDITSVATNLSNDGKLCIEAPKLEGVCGRSVPVKEASMDHHHIE
CCCCCEEEEEEEECCCCCCCEEEEEECCEEEEEEEEEEECCCCEEEEEEEEEEECCCCCCCCEEEEEECCCCCEEEEEECCCCCCCCEEEEEECCCCCCCCC
9889818999981699954079999899999999998630476589999999996899999010589854997089998369998870576674477532589

>Ce16B[*Caenorhabditis elegans*]
MSVNPFFDPRTLGEMVFGDNGRLDREYVPISENNDDLSDCRNEIVDTHEKFSVNLNVPDVKPEELKINLEGRKLSIKAEHQEIENDNISTTQTYSKSIVLPEDVDVTHLSSNL
SEDGKLLIEVPKVEAKKTNFFGFLSKFRCMPESQ
CCCCCCCCCCCCHHHHHCCCCCCCCCCCCCCCCCCCCCCCCEEEEECCCEEEEEEEECCCCCCCEEEEEECCEEEEEEEEEEEECCCCEEEEEEEEEEECCCCCCHHHEEEEE
CCCCEEEEEEECCCCCCCCCEEEECCEEEECCCC
999877785420345422112221222554345432246540368729859999981699954079999899999999998654237937999999999689999922337874
5997189998368887655225412114530479

>Ce17a[*Caenorhabditis elegans*]
MDRRFPPFSPFFNHGRFFDDVDFDRHMIRPYWADQTMLTGHRVGDAIDVVNNDQEYNVSVDVSQFEPEELKVNIVDNQLIIEGKHNEKTDKYGQVERHFVRKYNLPTGVRPEQ
IKSELSNNGVLTVKYEKNQEQQPKSIPITIVPKRN
CCCCCCCCHHHCCCHHHHHHCCCCCCCCCCCCCCCCCCCCCCCCCCCEEEEEECCEEEEEEEECCCCCCCEEEEEECCEEEEEEEEEECCCCCEEEEEEEEEEEECCCCCCHHH
EEEEECCCCCEEEEEECCCCCCCEEEEEEECCCCC
9988873111047402343214665212555677532222245663489981996999998069995407999989999999999864037843899999999994899999223
37874499818999817988876168889713579

>Ce17b[*Caenorhabditis elegans*]
MDRRFPPFSPFFNHGRRFFDDVDFDRHMIRPYWADQTMLTGHRVGDAIDVVNNDQEYNVSVDVSQFEPEELKVNIVDNQLIIEGKHNEKTDKYGQVERHFVRKYNLPTGVRPE
QIKSELSNNGVLTVKYEKNQEQQPKSIPITIVPKRN
CCCCCCHHHHHHHHHHHHHHCCCCCCCCCCCCCCCCCCCCCCCCCCCCEEEEEECCEEEEEEEECCCCCCCEEEEEECCEEEEEEEEEEECCCCEEEEEEEEEEEECCCCCCHH
HEEEEECCCCCEEEEEECCCCCCCEEEEEEECCCCC
9863111347764656554113577633366567642111224566347898199699999806999541799998999999999986403784289999999999589999922
337874399718999817988876168788723469

>Ce20A[*Caenorhabditis elegans*]
MSSLCPYTGRPTGLFRDFEDMMPYWAQRHSMLNNFNNIVPQQLNEVENTAQKFCVKLDVAAFKPEELKVNLEGHVLTIEGHHEVKTEHGFSKRSFTRQFTLPKDVDLAHIHTV
INKEGQMTIDAPKTGSNTTVRALPIHTSAGHAVTQKPSSTTTTGKH
CCCCCCCCCCCCCCHHHHHHHHHHCCCCCCCCCCHHHCCCCCCEEEEECCCEEEEEEEECCCCCCCEEEEEECCEEEEEEEEEEEECCCEEEEEEEEEEECCCCCCHHHEEEE
ECCCCCEEEEEECCCCCCCCCEEEEEECCCCCCCCCCCCCHHHHCC
99878888998641125666543204776410001210256642689719949999981699954179999899999999999875059759999999999589999911248888
4499718999826898888705765643675434432111122059

>Ce21a[*Caenorhabditis elegans*]
MSKTYSYTRVTTRSNGLPSKSPIEELPVYSPIYSSTFRTAVGSSSNSINTAGLDGLRTPMRSSGDDRARSSNNMATFNTTSSYNRTYEKKVIEEGSPNVRVTTQTHTAIPGMP
DLGNIHSSMLSNFPNLAISSPSVVGSQNGNLTSIRVTNTSFHAILDVSKYDADSLKVTVVDNNIIVEGSHGEKEDTYGTIESTFKRRFPLPKAVAPESVQSQLTADGHLTIDA
KAPEPKQEGARPIQIKVINTSAEQQKQ
CCCCCCCCEEEECCCCCCCCCCCCCCCCCCCCCCCCCCCCCCCCCCCCCCCCCCCCHHHHHCCCHHHCCCCCCCCCCCCCCCCCCCHHHHHHCCCCCCCCCCCCCCCCCCCCC
HHHHHHHHHHCCCCCCCCCCCCCCCCCCCCCEEEEECCCEEEEEEEECCCCCCEEEEEEECCEEEEEEEECCCCCCCCEEEEEEEEEEECCCCCCHHHEEEEECCCCEEEEEE
ECCCCCCCCCEEEEEEECCCCHHHCCC
98766530244224666677863125655676666544322443224451111110112211210100221100125777765431122110146776666666666546774
23333324430144334666432344678841578739918999991799933179999999999999732324789838988887884899999111488726997089998
168887777526756862773022159

>Ce21b[*Caenorhabditis elegans*]
MATFNTTSSYNRTYEKKVIEEGSPNVRVTTQTHTAIPGMPDLGNIHSSMLSNFPNLAISSPSVVGSQNGNLTSIRVTNTSFHAILDVSKYDADSLKVTVVDNNIIVEGSHGEK
EDTYGTIESTFKRRFPLPKAVAPESVQSQLTADGHLTIDAKAPEPKQEGARPIQIKVINTSAEQQKQ
CCCCCCCCCCCCCCCHHHHHHCCCCCCCCCCCCCCCCCCCHHHHHHHHHHCCCCCCCCCCCCCCCCCCCCCEEEEECCCEEEEEEEECCCCCCEEEEEEECCEEEEEEEEEEE
CCCCCEEEEEEEEEEECCCCCCCCEEEEEECCCCCEEEEEECCCCCCCCCEEEEEEECCCCCCCCCC
99898888665665202343146766777644455567741444543553125433356531012457884157872991899998179993307999989999999975321
2688269999998884899999010589844997089998168887678527878871774022159

>Ce43a[*Caenorhabditis elegans*]
MTLATRHADFTRDKLWKENDLWLDDFRDWPLDWPKPRDFFHRFSRDVDSWWKDWPTDWPRMDAVMPRFSSQLDRMDRNWRSDPYWMNLYPRWAEPIFKEGIDVNSNVVNDDRR
FAVDMDCYQFRPEEIQVKTLDDTLMIEGRHEDIRDKDNFTKMYFVRKYQLPRDVDFNSIQSSIDAKGRLQVEAGKFNNMALQGRERMIPIEGAGHHSPRFENGTLRSQRGPNS
PIHVQTEHDGRSVSSRSGSRLNDSPGSRDVYSSHSYSYHRSDSRNRLSPNDVNITRNDNRTYSPVTPRITTSGS
YNTAGNANLHEERSSSRAQSHRSESRNGGYRVESPVSTTTGILRNGNSDSPNSTQREYRSIQILRKTY
CCCCCCCCCCCCCCCCCCCCCCCCCCCCCCCCCCCCHHHHHHCCCCHHHHHHHCCCCCCHHHHHHHHHHHHHHHHHHHCCCCCCCCCCCCCCHHHHHCCCCCCCCCEEEECCE
EEEEEEECCCCCCEEEEEEECCEEEEEEEEECCCCCCCEEEEEEEEEEECCCCCCCCEEEEEECCCCCEEEEECCCCCCCCCCCCEEEEEEECCCCCCCCCCCCCCCCCCCCC
CCCCCCCCCCCCEEEECCCCCCCCCCCCCCCCCCCCCCEEEEECCCCCCCCCCCCCCCCCCCCCCCCCCCCCCCCCCCCHHHHCCCHHHHHCCCCCCCCCCCCCCCCCCCCCC
CCCCCCCCCCCCCCCCCCCCCEEECCCCC
98655644553343334578776754357877765303342123430244421112342245543132214555431112564323456530122220246776412660984
89999807999530899998999999999611147995588778888857999994405999549970899981798876667752775553365433223431000135777
64345201484046624654456666554444466420112321567887667752212344676533334554432110110110111100122333566662135776421
11125787545667710111001122259

>Ce43b[*Caenorhabditis elegans*]
MTLATRHADFTRDKLWKENDLWLDDFRDWPLDWPKPRDFFHRFSRDVDSWWKDWPTDWPRMDAVMPRFSSQLDRMDRNWRSDPYWMNLYPRWAEPIFKEGIDVNSNVVNDDRR
FAVDMDCYQFRPEEIQVKTLDDTLMIEGRHEDIRDKDNFTKMYFVRKYQLPRDVDFNSIQSSIDAKGRLQVEAGKFNNMALQGRERMIPIEGAGHHSPRFENGTLRSQRGPNS
PIHVQTEHDGRSVSSRSGSRLNDSPGSRDVYSSHSYSYHRSDSRNRLSPNDVNITRNDNRTYSPVTPRITTSERTVTPEQRSPGRKAFETIRNNFERGSYNTAGNANLHEERS
SSRAQSHRSESRNGGYRVESPVSTTTGILRNGNSDSPNSTQREYRSIQILRKTY
CCCCCCCCCCCCCCCCCCCCCCCCCCCCCCCCCCCCHHHHHHCCCCHHHHHHHCCCCCCHHHHHHHHHHHHHHHHHHHHCCCCCCCCCCCCCHHHHHCCCCCCCCCEEECCCE
EEEEEEECCCCCCEEEEEEECCEEEEEEEEECCCCCCCEEEEEEEEEEECCCCCCCCEEEEEECCCCCEEEEECCCCCCCCCCCCEEEEEEECCCCCCCCCCCCCCCCCCCCC
CCCCCCCCCCCCEEEECCCCCCCCCCCCCCCCCCCCHHHHHHHCCCCCCCCCCCCCCCCCCCCCCCCCCCCCCCCCCCCCCCHHHHHHHHHHHHHCCCCCCCCCCCECCCCCC
CHHHHCCCCCCCCCCCCCCCCCCCCHHHHHCCCCCCCCCCHHHCCCCCCCCCCC
98654533443244556778775532237777652145532222023444212123422455431321144555422114643224566311112202467764215519844
89999817999431899998999999999610147996578778889967999994416999649971899972899876667752775553366432212343111246787
41123234675235524654456666544433454011112102456788888520110013676653224567776554420233344445531244456665410134665
114321341012578755688654201442278888778723324321021259

Cluster B3 – Multiple sequence alignments

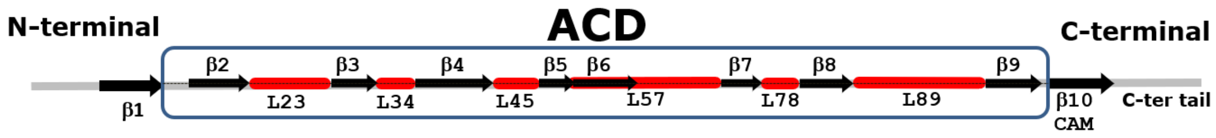

PROMALS3D alignment

PROMALS3D multiple sequence and structure alignment server http://prodata.swmed.edu/promals3d/promals3d.php (Pei et al. 2008)

```
Conservation:                                     8 5 65   6 6 56 6  5    5 7 5        5  5   5   7  69         6 6  9 8 6 5
Ce43a+b                     1  EPIFKEGIDVNSNVVNDDRRFAVDMDCY-QFRPEEIQVKTLD--DTLMIEGRHEDIR---DKDNFTKMYFVRKYQLPRDVDFNSIQSSIDAKGRLQVEAG  94
Ce17a+b                     1  TMLTGHRVGDAIDVVNNDQEYNVSVDVS-QFEPEELKVNIVD--NQLIIEGKHNEKT---DKYGQVERHFVRKYNLPTGVRPEQIKSELSNNGVLTVKYE  94
Ce20A                       1  NNFNNIVPQQLNEVENTAQKFCVKLDVA-AFKPEELKVNLEG--HVLTIEGHHEVKT----EHGFSKRSFTRQFTLPKDVDLAHIHTVINKEGQMTIDAP  93
Ce16B                       1  SENNDDLSDCRNEIVDTHEKFSVNLNVP-DVKPEELKINLEG--RKLSIKAEHQEIE---NDNISTTQTYSKSIVLPEDVDVTHLSSNLSEDGKLLIEVP  94
Ce16.48                     1  DHNSFNFSDNIGEIVNDESKFSVQLDVS-HFKPEDLKIELDG--RELKIEGIQEKKS----EHGYSKRSFSKMILLPEDVDLTSVKSAISNEGKLQIEAP  93
Ce16A+11                    1  ISGTFNPMTDDSEIMNSNDKFAVNLNVS-NFKPEELKVNLEG--RQLSIQGEHDVEN----EHGASRKSFSRMILLPEDVDITSVATNLSNDGKLCIEAP  93
Ce16.11+1A                  1  TPVCRGSPSESSEIVNNDQKFAINLNVS-QFKPEDLKINLDG--HTLSIQGEQELKT----EHGYSKKSFSRVILLPEDVDVGAVASNLSEDGKLSIEAP  93
Ce16.2a+b                   1  APVCRISPSESSEIVNNDQKFAINLNVS-QFKPEDLKINLDG--RTLSIQGEQELKT----DHGYSKKSFSRVILLPEDVDVGAVASNLSEDGKLSIEAP  93
Ce16.41                     1  DHNSFNFSDNIGEIVNDESKFSVQLDVS-HFKPENLKIKLDG--RELKIEGIQETKS----EHGYLKRSFSKMILLPEDADLPSVKSAISNEGKLQIEAP  93
Ce20B_C.elegans             1  RPRSPGPVAGAGEITNTSHGFTIEIDVF-HFMPEEIKVVLTD--DTLSISGERFEST---GDGHTLRRSFSRKYSIPDDVHLDTIRSHLTNSGVLIINGS  94
Ce21a+b                     1  PSVVGSQNGNLTSIRVTNTSFHAILDVS-KYDADSLKVTVVD--NNIIVEGSHGEKE---DTYGTIESTFKRRFPLPKAVAPESVQSQLTADGHLTIDAK  94
Cri34                       1  GCPRFAGRGGCPCKKTLAEDFKVTFDVK-SFNPEEISVKVKG--REIFVDGKHDERA---DEQGFVSRQFTRRFIVPDEFDTDTIATYLDIEGRMTIVAS  94
Cri23                       1  IKKASQNDLSTKSKEQEVKDFSITFDFS-SFKPEEISVKVKD--RDVIIEAKHEERE---DEHGFISRQLTRKFILPDEYDLDTISTYLNADGKMTIKAL  94
Cri27                       1  LQRQMMGDINEVIPTTGKDGFQVCMDVQ-QFKPSEITVKTTD--NSVVIEGKHEERQ---DEHGQISRHFIRRYTLPKGYDPKSISSELSSDGVLTIKAP  94
Cri24                       1  REAVGELAKDQKNLHFGKDGFQACVDVH-HFAPSEITVKTVD--HTVTIEGKHEERE---DGHGSVQRHFVRKYVLPKEYDMNNVHSTLSSDGVLTIKAP  94
Cri22                       1  NHSAKSMIKDQKNLHFGKDGFQASVDVH-HFQPSEITVKTIE--NQIVVEGRHEERD---DGHGSIQRHFVRKYVLPKEYDMNSVHSTLSSDGVLTIKAP  94
ID13_A.lacteospumosus       1  RRQPAQQASGMSEVQNTDKDFRVNLDVS-HFAPEELSIKTVD--NRVIIHGKHEEKQ---DEHGFIQREFRRTYVLPTDVDPNTVKSSLTADGVLSIKAP  94
ID4_O.vulgaris              1  AQQHSAVSTGFSEVSNDEKEFKVRLDVS-HFNPDEITLKVID--NRIVIHAKHEEKQ---DEHGYIQREFKRQYLLPRECDAANVTSMLASDGVLTIRAS  94
ID2_E.fetida                1  RRQPAQQASGMSEVQNTDKDFHVNLDVS-HFSPEEISIKTVD--NRVIIQGKHQEKQ---DEHGFIERQFKRTYVLPNDVDPNTVKSSLSADGVLSIKAP  94
ID14_C.matritensis          1  RRQPAQQASGMSEVQNTDAAFHVNLDVS-HFAPEELTIKTAD--NRVMIHGKHEEKQ---DEHGFIQREFKRTYVLPNDVDPNTVKSSLSADGVLSIKAP  94
ID5_C.matritensis           1  KRQPTQQASGVSEVANTDKKFQVNLDVS-HFRPEELSIKTSG--NRVIIHGKHEEKQ---DEHGFIQREFRRTYVLPNDVDPETVKSSLSGDGILAVVAP  94
ID6_C.matritensis           1  MTGRKGASSQEVRQQKEEEDFSVRLDVS-HFQPEELTVKTTG--NRVIVHARHEEKQ---DEHGYIEREFTRTYLLPEDVDPDTVKSCLSGDGVLSIEAP  94
ID8_A.marina                1  -------TSGASEVAMTDKDFKVNLDVS-HFKPGEITIKTVD--NRVVVHGKHEERP---DEHGFIQREFKRSYPLPEDVKAELVTSSLSADGI------  81
ID4_M.pitelkai              1  EVFKALKQTGLAEVKCDDSQFSVMLDVT-QFSPEEVEVKTND--NKLTIHAKHEEKE---DEHGFISREFTRTYLLPKNVDPLTLTTKLTSDGVLNIAAP  94
ID8_P.australis             1  RHGVISAESALPEIKFTDGEFRVRVDVQ-HYTPEEIGVKIAD--NTIKISGKHETKH---DDYGMVAREFTREFSIPEEIDQESLECQISEDGFLTVMGS  94
ID5_L.gigantea              1  ---------------LNKKEFEVNVDVH-HFESENIHVKLSN--NRLTISGKHESKQ---DEHGYVSREFSREFTVPENVDAETMTSSITEEGVLVIRAK  79
ID4_P.gouldii               1  DFPRHRCRGREMEVKYDRKVFEVRLDVS-LFTPEDIKVTVNESDRILVISAKHKEKP---DDHGCISREFNRQILIPENVEMEEFQSLLTNDGHLVVRAK  96
ID9_P.californica           1  RHGSRRVGSKQVEVKYDDKVYEVKVDVH-EFSPEELNVKIVE--DRLVISGKLEDRP---DDHGFISREMKREFIIPENVEVETIESHLTDEGFLKICGK  94
ID6_M.pitelkai              1  RREQPASINQDVEVKYNEKEFSIKLDVQ-DYSEGELKVKVVE--DRVVVSGKVQEKQ---DEHGFVSQEFSRQFLIPENVDPETIESSICAEGVLTIQGQ  94
ID16_N.dendritica           1  YDVVRTGAAREIEVKYNDKHFEVKLDVN-NFSPEEVNVKLQG--DRLIVGAKHEEKR---DDYGFVSRELSRQFSVPEDVDAESLESVLTNDGYLVIHGK  94
ID3_E.torquata              1  AAARGSRPSKDVQVKYTDQVFEVRLDVN-QFAPEELNIKVGG--ERLTITGKQQERP---DEHGVISREFMRQFAIPENVETESFESQLTTDGFLVIKGK  94
ID3_B.viridis               1  SLNFEYGDTKNIQVTHNKNHFEVKLDVE-NYEPDDLEVKVVD--RQLTISGKCEQKS---DKVGLVSQEFTRVLQIPEDVDSESLETLLTSDGYLVVSGK  94
ID4_A.marina                1  FPESVNVASRDMKIYQTDKLFEVRLNVS-QFSPEELTVRQSG--SRLIITGSHKQES----EGQSLSREFTREITLPEHVEQDSLESVFTGSGQLILRGK  93
Cri17                       1  FLTNHRPKSFKQLLIGKDGFFRTAVDIH-DFTKEEVEVKTIG--HTIMITAAHDEKE---DEFGHIERRFSRKYVLPLDLDIEKISSWIS-KGVLYIKVP  93
Ce12.6                      1  WDWPLQKGDGVVNVLDDDDHFEVGLEAH-NFLPKEIEVKNIG--ELLEIHMEHNVKK---DSFGDVSRNITRCYKLPKNVDMKTIKSNLDSHGILHIEAR  94
Ce12.3                      1  WDWPFQKGDGVVKVLDYEDHFEVGLEAH-NFLPNEIDVKNIG--EFLEIHMAHTTKD---DKFGSITRSITRCYRLPKGTDPATIKSKLDGSGILHISGN  94
Ce12.2                      1  WDWPLQHNDGVVKVHNTKEKFEVGLDVQ-FFTPKEIEVKVSG--QELLIHCRHETRS---DNHGTVAREINRAYKLPDDVDVSTVKSHLATRGVLTITAS  94
Ce12.1a+b                   1  WDWPLQHNDGVVKVTNTSEKFEVGLDAG-FFGPNDIDVKVNG--IEIIIHLRHDLLQNRPTEYGIVNREVHRTYKLPEDVDPSTVRSHLNSSGVLTITAN  97
Consensus_aa:                  ........s...pl..spp.Fpl.l-l..p@pPp-lplph.s..ppl.lptchppcp...sp.s.hp+p@pR.h.lPcsl-.pslpo.lsspG.L.lpt.
Consensus_ss:                            eeeee   eeeeeeee    hhheeeeee   eeeeeeee         eeeeeeeeeee      hhheeeee   eeeeee
```

MEGA 7 alignment (Kumar et al. 2016)

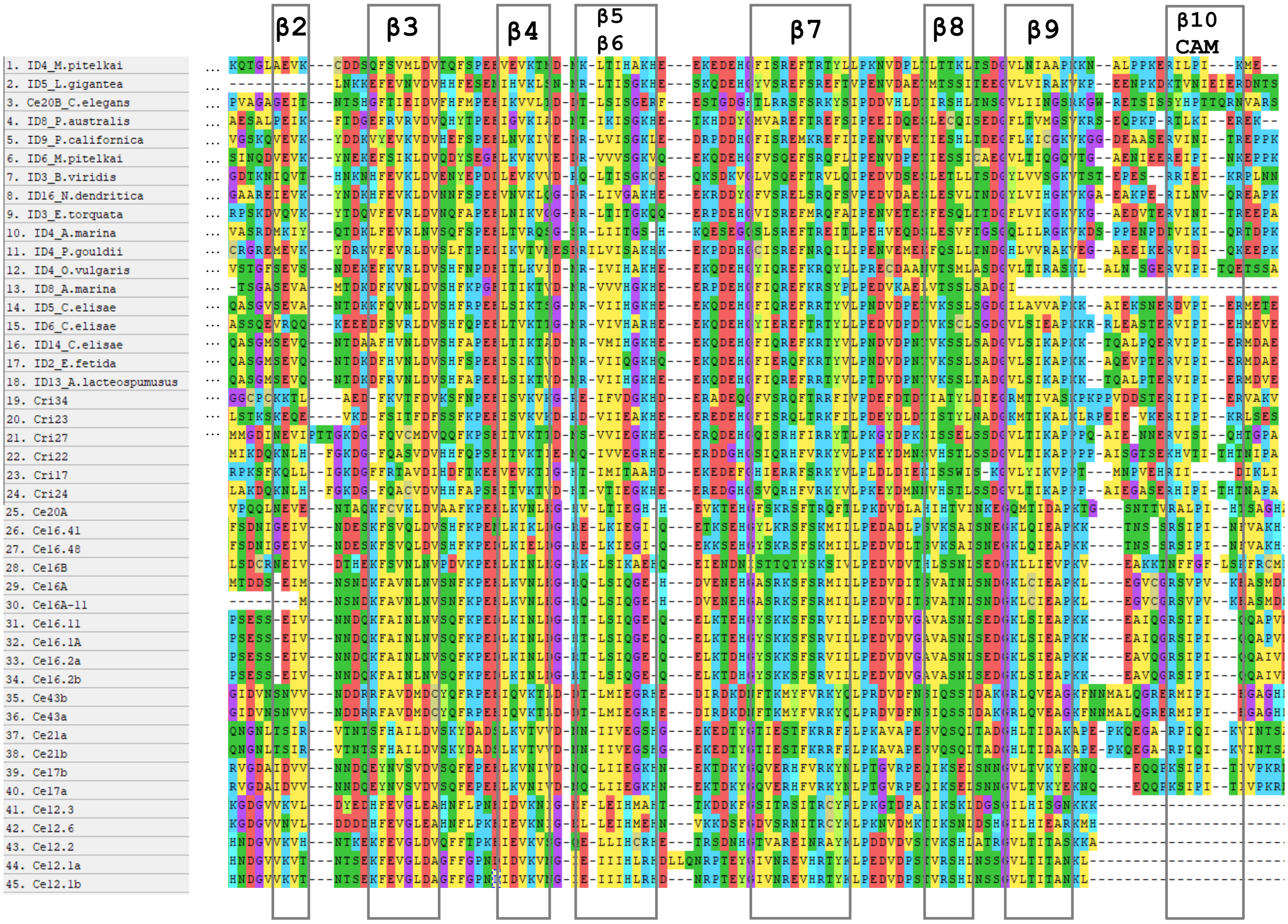

3 Violin plots of theoretical pI, length, molecular weight, and grand average of hydropathicity (GRAVY) of annelid monomeric sHsps and their respective ACDs (between β3 and β9) for each cluster. Includes Supplementary Figures 15 and 16.

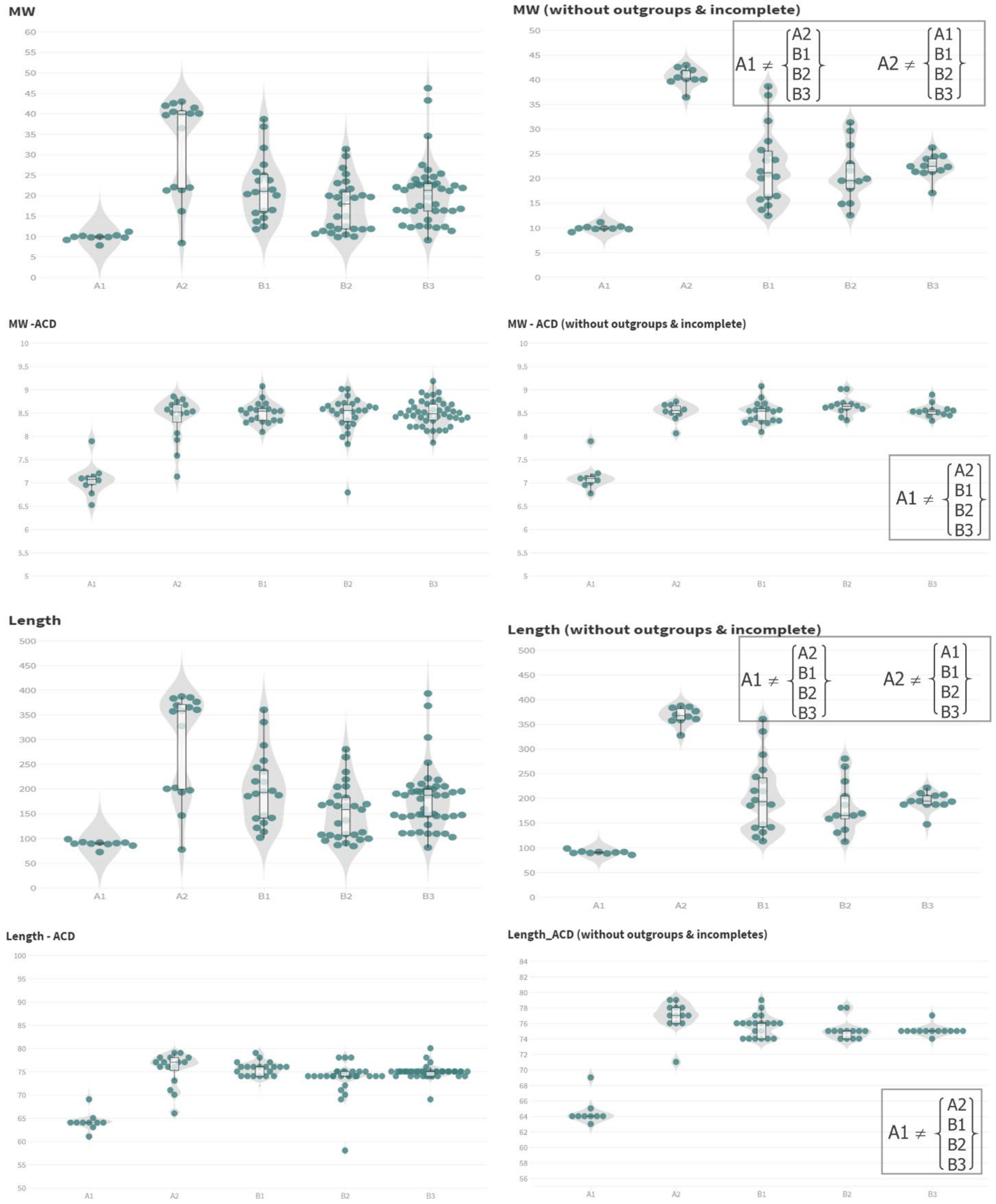

Supplementary Figure 15. Violin plots (https://flourish.studio/) of theoretical length, molecular weight, pI and grand average of hydropathicity (GRAVY) of annelid monomeric sHsps and their respective ACDs (between β3 and β9) for each cluster. Significant differences between clusters are highlighted (boxes in right) according to ANOVA analysis (normal distributions) or Kruskal-Wallis analysis (non-normal distributions) ($p \leq 0.05$).

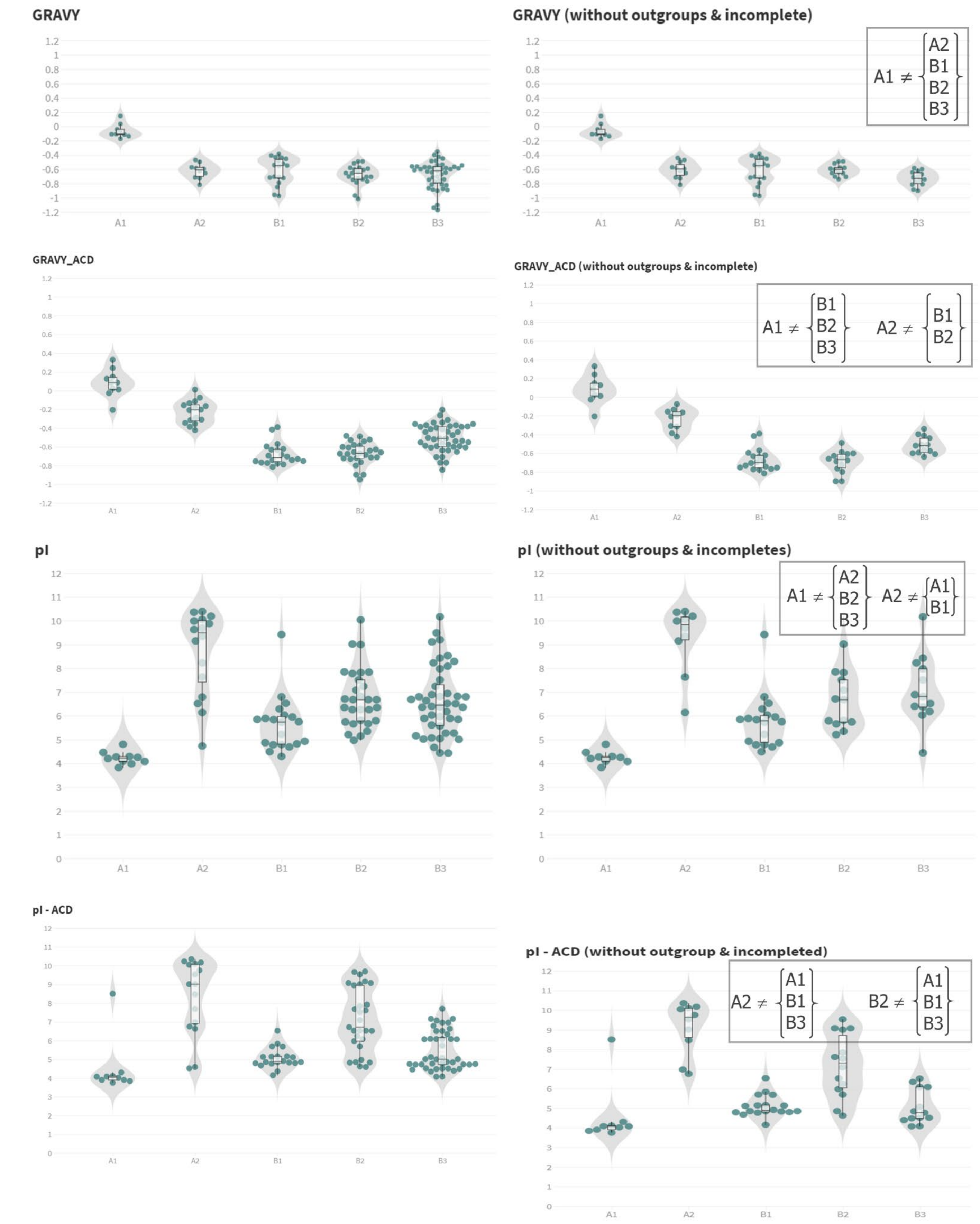

Supplementary Figure 16. Violin plots (https://flourish.studio/) of theoretical pI and grand average of hydropathicity (GRAVY) of annelid monomeric sHsps and their respective ACDs (between β3 and β9) for each cluster. Significant differences between clusters are highlighted (boxes in right) according to ANOVA analysis (normal distributions) or Kruskal-Wallis analysis (non-normal distributions) ($p \leq 0.05$).

4 Logo presentation for Clusters A and B. Logos for clusters B2 and B3 calculated including outgroups are also shown. Includes Supplementary Figures 17 to 19.

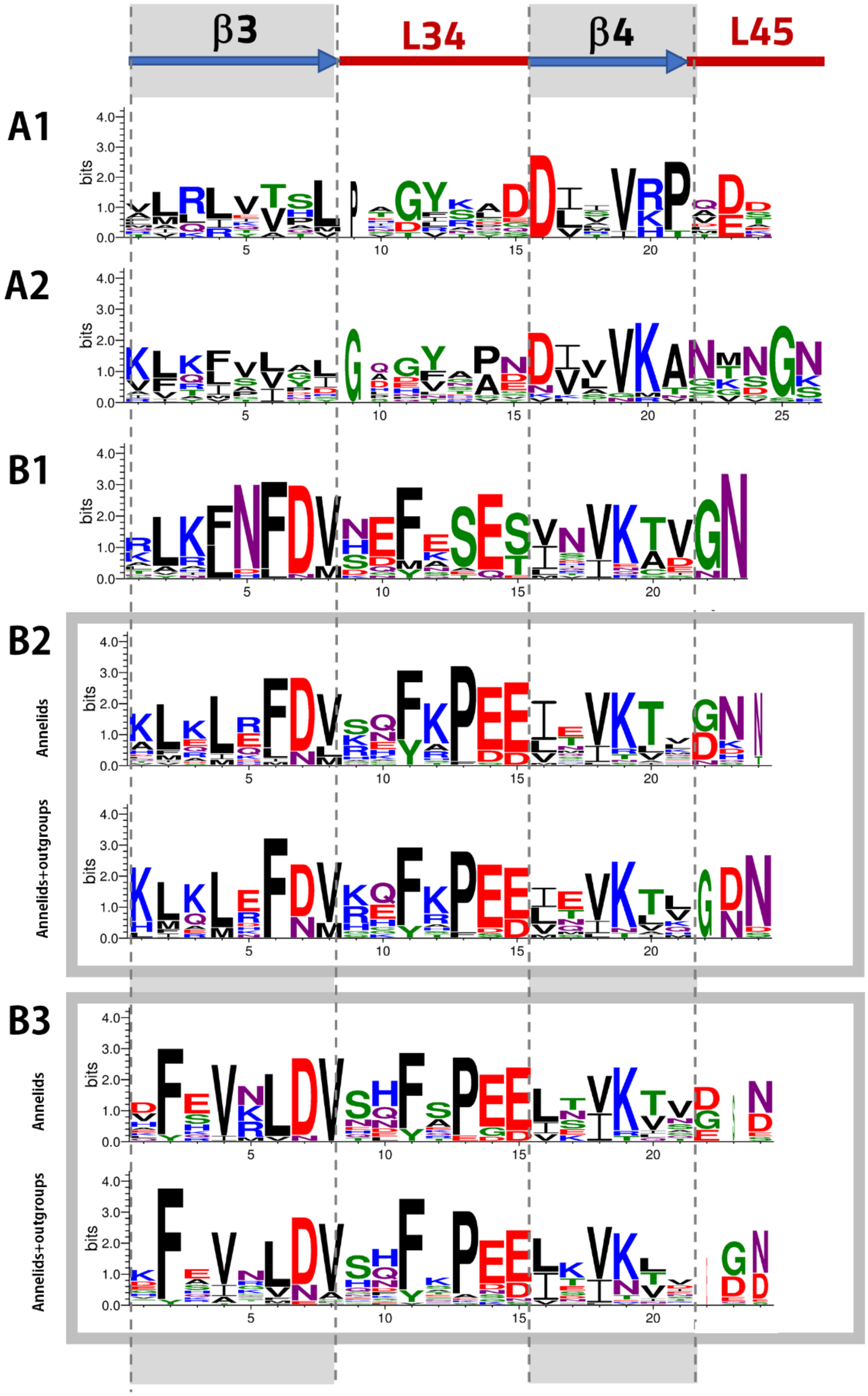

Supplementary Figure 17. Logo presentation using WebLogo3 program (Crooks et al. 2004) for Clusters A and B (**β3 to L45 region**). The height of each letter is proportional to its frequency. Amino acids are colored according to their chemical properties: acidic (D,E) in red, basic (K,R,H) in blue, polar (G,S,T,Y,C) in green and (N,Q) in purple, and hydrophobic (A,V,L,I,P,W,F,M) in black. Logos for Clusters B2 and B3 are calculated with and without the outgroup sequences.

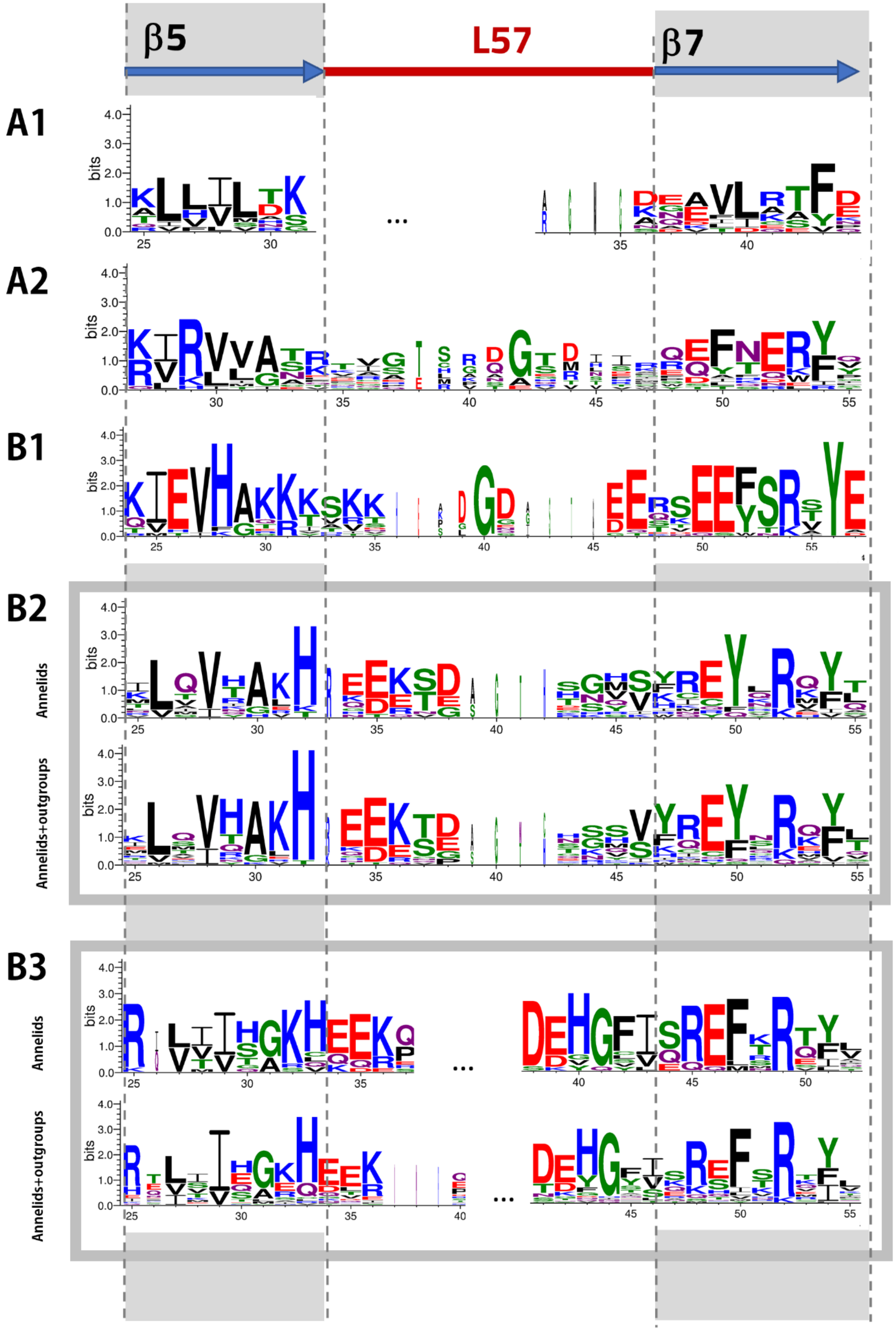

Supplementary Figure 18. Logo presentation using WebLogo3 program (Crooks et al. 2004) for Clusters A and B (**β5 to β7 region**). The height of each letter is proportional to its frequency. Amino acids are colored according to their chemical properties: acidic (D,E) in red, basic (K,R,H) in blue, polar (G,S,T,Y,C) in green and (N,Q) in purple, and hydrophobic (A,V,L,I,P,W,F,M) in black. Logos for Clusters B2 and B3 are calculated with and without the outgroup sequences.

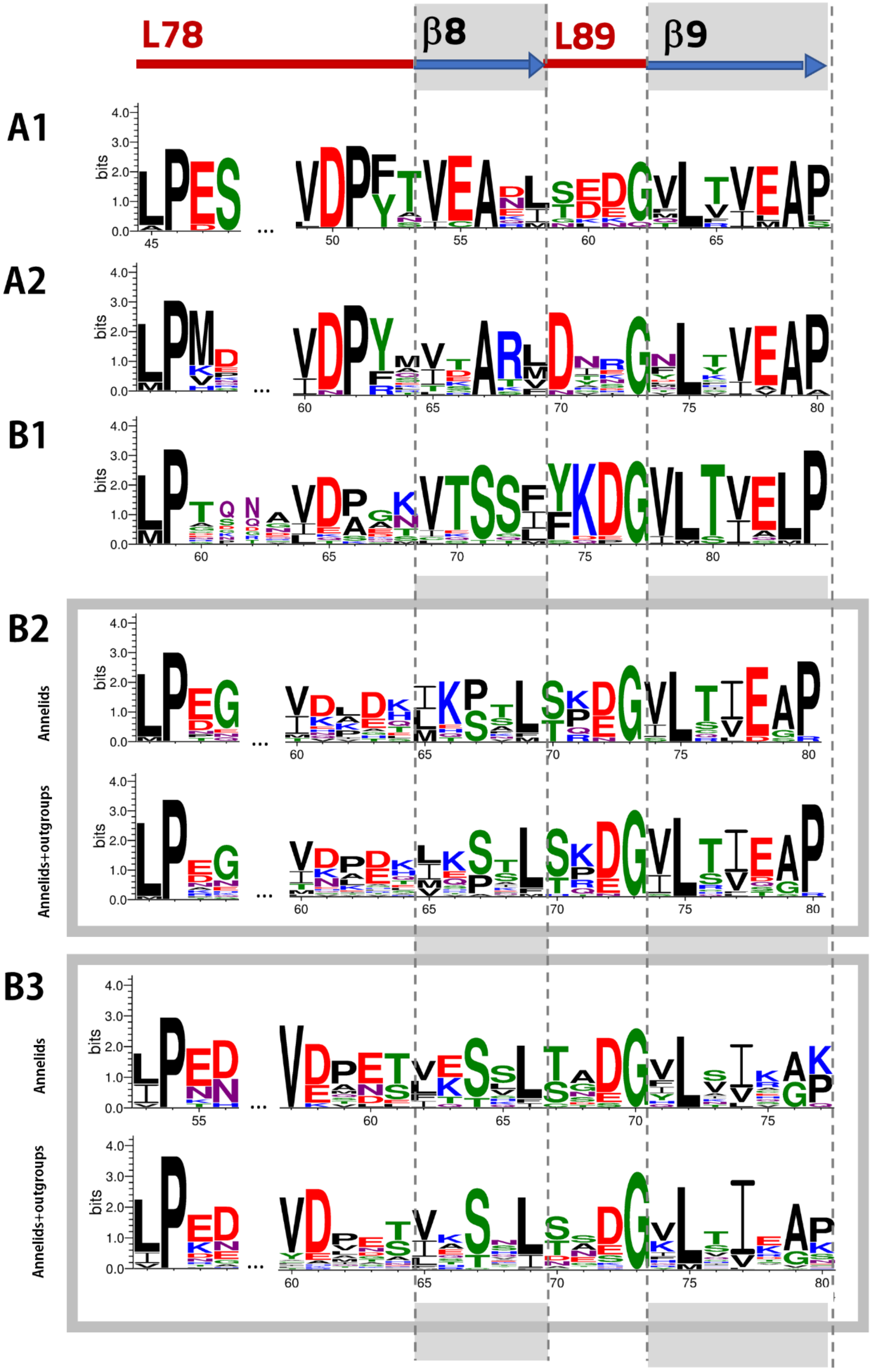

Supplementary Figure 19. Logo presentation using WebLogo3 program (Crooks et al. 2004) for Clusters A and B (**L79 to β9 region**). The height of each letter is proportional to its frequency. Amino acids are colored according to their chemical properties: acidic (D,E) in red, basic (K,R,H) in blue, polar (G,S,T,Y,C) in green and (N,Q) in purple, and hydrophobic (A,V,L,I,P,W,F,M) in black. Logos for Clusters B2 and B3 are calculated with and without the outgroup sequences.

5 References

Crooks GE, Hon G, Chandonia J-M, Brenner SE. 2004. WebLogo: A Sequence Logo Generator. *Genome Res.* 14:1188–1190.

Kumar S, Stecher G, Tamura K. 2016. MEGA7: Molecular Evolutionary Genetics Analysis Version 7.0 for Bigger Datasets. *Mol. Biol. Evol.* 33:1870–1874.

Pei J, Kim B-H, Grishin NV. 2008. PROMALS3D: a tool for multiple protein sequence and structure alignments. *Nucleic Acids Res.* 36:2295–2300.

Poulain P, Gelly J-C, Flatters D. 2010. Detection and Architecture of Small Heat Shock Protein Monomers. *PloS One* 5:e9990.

Yan R, Xu D, Yang J, Walker S, Zhang Y. 2013. A comparative assessment and analysis of 20 representative sequence alignment methods for protein structure prediction. *Sci. Rep.* 3:2619.
