# Supplementary material for: Understanding Diversity, Evolution, and Structure of Small Heat Shock Proteins in Annelida Through in Silico Analyses
Source: Front Physiol. 2022 Apr 13;13:817272. doi: 10.3389/fphys.2022.817272 (PMC9075518; doi:10.3389/fphys.2022.817272)
Supplement: Supplementary file 1 [file DataSheet1.ZIP › SM_MFuente&MNovo/Tables_S1-S3.pdf]

Para obtener la mejor experiencia, abra esta cartera PDF en Acrobat X o Adobe Reader X, o en alguna versión posterior.

¡Consiga Adobe Reader ahora!
